# Umbilical cord extracts improve diabetic abnormalities in bone marrow-derived mesenchymal stem cells and increase their therapeutic effects on diabetic nephropathy

**DOI:** 10.1038/s41598-017-08921-y

**Published:** 2017-08-16

**Authors:** Kanna Nagaishi, Yuka Mizue, Takako Chikenji, Miho Otani, Masako Nakano, Yusaku Saijo, Hikaru Tsuchida, Shinichi Ishioka, Akira Nishikawa, Tsuyoshi Saito, Mineko Fujimiya

**Affiliations:** 1 Second Department of Anatomy, Sapporo Medical University, Sapporo, Japan; 2 Department of Diabetic Cellular Therapeutics, Sapporo Medical University, Sapporo, Japan; 3 Department of Obstetrics and Gynecology, Sapporo Medical University, Sapporo, Japan; 4 Department of Gynecology and Obstetrics, NTT Sapporo Hospital, Sapporo, Japan

## Abstract

Bone marrow-derived mesenchymal stem cells (BM-MSC) has been applied as the most valuable source of autologous cell transplantation for various diseases including diabetic complications. However, hyperglycemia may cause abnormalities in intrinsic BM-MSC which might lose sufficient therapeutic effects in diabetic patients. We demonstrated the functional abnormalities in BM-MSC derived from both type 1 and type 2 diabetes models *in vitro*, which resulted in loss of therapeutic effects *in vivo* in diabetic nephropathy (DN). Then, we developed a novel method to improve abnormalities in BM-MSC using human umbilical cord extracts, namely Wharton’s jelly extract supernatant (WJs). WJs is a cocktail of growth factors, extracellular matrixes and exosomes, which ameliorates proliferative capacity, motility, mitochondrial degeneration, endoplasmic reticular functions and exosome secretions in both type 1 and type 2 diabetes-derived BM-MSC (DM-MSC). Exosomes contained in WJs were a key factor for this activation, which exerted similar effects to complete WJs. DM-MSC activated by WJs ameliorated renal injury in both type 1 and type 2 DN. In this study, we developed a novel activating method using WJs to significantly increase the therapeutic effect of BM-MSC, which may allow effective autologous cell transplantation.

## Introduction

Mesenchymal stem cells (MSCs) are considered as the most attractive cell source for regenerative medicine. MSCs have been highlighted because of their multi-potentialities, such as self-renewal ability, pluripotency, low antigenicity, less toxic, ease of culture and expansion *in vitro* to obtain sufficient cells for treatment. MSCs are obtained from bone marrow, skeletal muscle, synovium, dental pulp, adipose tissue, umbilical cord (UC) tissues, UC blood and placenta^1, 2^. Among these MSCs, because bone marrow-derived MSCs (BM-MSC) have been the most well-studied for their safety and efficacy in systemic administration, their use has already been attempted in clinical trials, including in graft-versus-host disease (GVHD)^3, 4^, autoimmune diseases^5–9^ and chronic inflammatory disease^10–14^, as therapeutic applications for tissue repair, regeneration and immune regulation.

Notably, the number of people in the world with diabetes is more than 380 million people. We have previously reported the powerful effects of BM-MSC and its paracrine effects of trophic factors and exosomes for diabetic nephropathy (DN)^15^. The efficacy of BM-MSC for DN^16–18^ and diabetic retinopathy^19–21^ has also been reported from another groups. Systemic high glucose levels induce abnormal metabolites in cells which elicit inflammatory responses in parenchymal cells and stromal cells of various organs, promoting fibrosis and irreversible damage. Indeed, DN is an intractable disease consisting of the degeneration of and chronic inflammatory changes in glomeruli, renal tubules and stromal cells. Therefore, BM-MSC is focused upon as a novel therapeutic modality for diabetic complications because of their strong immune regulatory functions and tissue regenerative actions.

Autologous transplantation of BM-MSC has great benefits because of the low risk of rejection, exogenous infection and the low ethical hurdle for the source of MSCs. However, hyperglycemia inflicts abnormal properties on bone marrow stem cells. Impairment of BM-MSC has been reported in T1D mice and high glucose exposed human MSCs. Gu *et al*. reported that overexpression of the cell aging factor p21 induced growth inhibition and apoptosis of BM-MSC in NOD mice^22^. Similarly, Khan *et al*. reported that hyperglycemia affected the function of MSCs adversely via oxidative stress and senescence in streptozotocin (STZ)-induce diabetic mice^23^. These reports suggest that BM-MSC derived from diabetes (DM-MSC) is not appropriate for cell therapies because of their abnormal functionality. However, the therapeutic effect of abnormal DM-MSC for diabetic complications has not yet been clarified. Especially, little is known about abnormalities of DM-MSC in type 2 diabetes (T2D) despite patients with T2D accounting for a large proportion. Therefore, we first aimed to clarify the abnormalities of DM-MSC derived from both T1D and T2D models and investigate the therapeutic effects in DN *in vivo*.

To perform the autologous transplantation with abnormal DM-MSC, cells are required to be modified into functional cells to ensure sufficient number of cells within an appropriate period and obtaining certain therapeutic effects. In this study, we developed a novel activator, human UC extracts, which we named Wharton’s jelly extract supernatant (WJs). UC is composed of embryonic tissues, including umbilical vein, umbilical artery, Wharton’s jelly (WJ) and amniotic membrane. UC blood, WJ and amnion are the source of fetal appendage-derived MSCs, which are reported to be better for cell proliferation and expansion than BM-MSC^24^. WJ and perivascular tissues contain a variety of biologically active substances including growth factors, cytokines, extracellular matrixes and micro-vesicles, which are components of the niche for fetal appendage-derived MSCs and provide the physiological environment to preserve MSCs properties. Therefore, we hypothesized that these components might activate abnormal DM-MSC and induce functional improvement. Wharton’s jelly extracts has been highlighted as a biomaterial to coat cell culture plates or a xeno-free substitute alternative to FBS^25, 26^. However, it has not been clarified whether fetal appendage extractions are valid for the functional improvement of abnormal BM-MSC derived from a disease model, especially for diabetes. Thus, we next aimed to investigate the efficacy of WJs to improve cell functions of DM-MSC *in vitro*. Furthermore, we performed an experiment to prove the therapeutic effects of activated DM-MSC on a DN model *in vivo*.

We developed this new activator and activating method to enhance the therapeutic effect of autologous transplantation. This method will enable the development of autologous cell therapies using BM-MSC, not only for diabetic patients but also for a variety of diseases in which autologous BM-MSC becomes abnormal.

## Results

### Morphology and proliferative ability were abnormal in BM-MSC derived from STZ-induced diabetic rats (STZ-MSC) and Otsuka Long-Evans Tokushima Fatty (OLETF) diabetic rats (OLETF-MSC)

**Figure 1:**
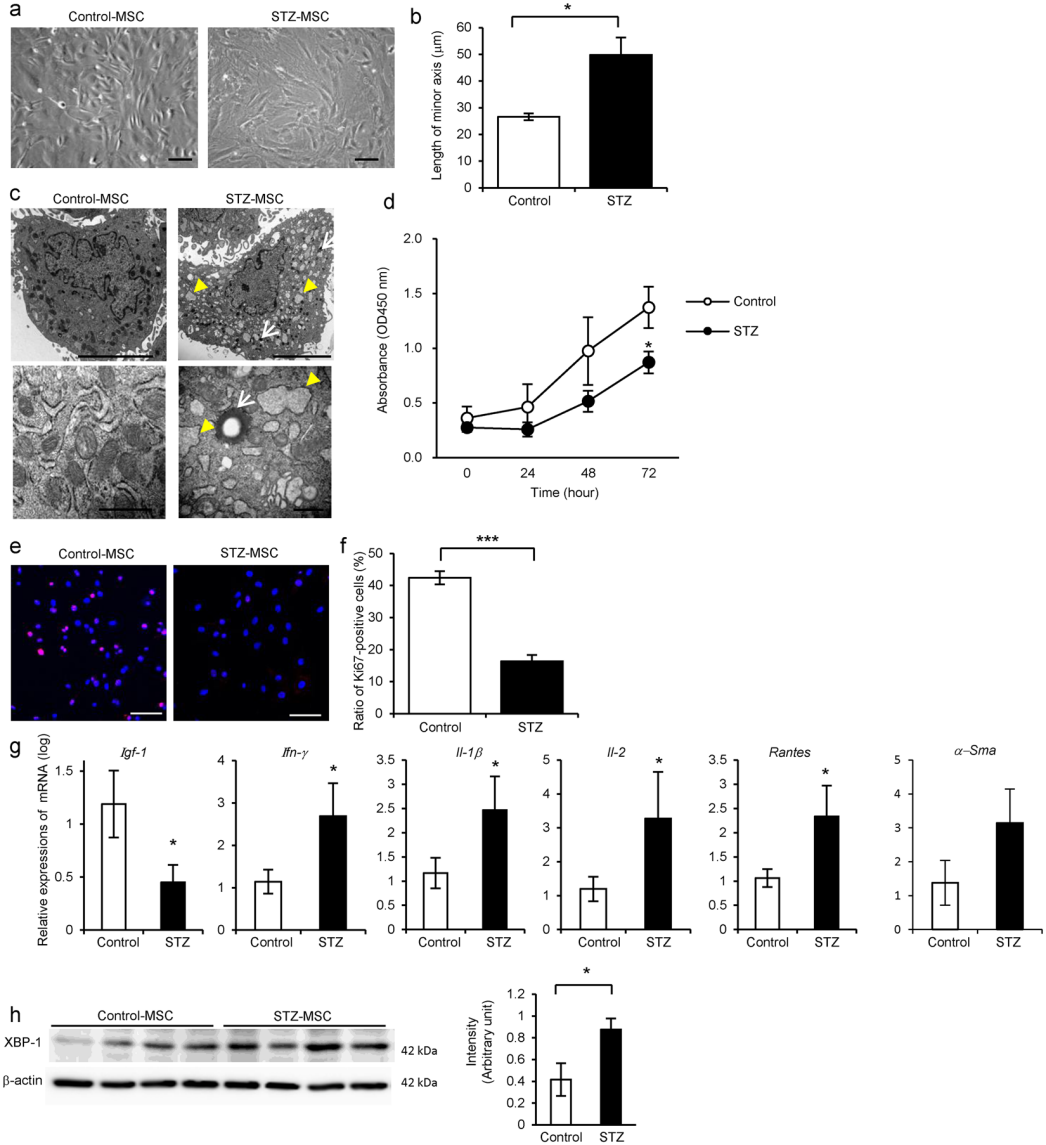
Abnormalities of bone marrow-derived mesenchymal stem cells (BM-MSC) isolated from streptozotocin (STZ)-induced diabetic rats (STZ-MSC) compared with control rats (Control-MSC). (**a**) Phase contrast images of Control-MSC and STZ-MSC (passage 3). Bar: 100 µm. (**b**) Quantitative analysis of morphological findings of BM-MSC. The lengths of the minor axis in phase contrast images were measured in all cells in five panels of each BM-MSC isolated from individual animals. Values are means ± SE of Control-MSC (n = 3) and STZ-MSC (n = 4). *P < 0.05. (**c**) Transmission electron microscopy (TEM) images of BM-MSC. Upper panels show the entire cell at low magnification, whereas lower panels show cell organelles at high magnification. White arrows show mitochondria degenerations. Yellow arrow heads show abnormal dilation of the endoplasmic reticulum (ER). Bar: 5 µm in upper panels and 500 nm in lower panels. (**d**) MTT proliferation assays of Control-MSC and STZ-MSC. Absorbance at 450 nm was measured 0, 24, 48 and 72 hours after the addition of MTT. Values are means ± SE of the control (n = 5) and STZ (n = 5). *P < 0.05. (**e**) Immunofluorescence staining of BM-MSC with anti-Ki-67 antibody (red). DAPI was used for counterstaining nuclei (blue). Bar: 100 µm. (**f**) The average ratio of Ki-67-positive cells to the total cell count. The number of Ki-67-positive cells and nuclei were counted in five panels of each BM-MSC isolated from individual animals. Values are means ± SE of the control (n = 4) and STZ (n = 5). ***P < 0.001. (**g**) Relative expressions of mRNA in BM-MSC. Values are means ± SE of the control (n = 4) and STZ (n = 3). *P < 0.05. (**h**) Western blot analysis of Control-MSC (n = 4) and STZ-MSC (n = 4) using anti-XBP-1 and β-actin antibodies. Relative amounts of protein are normalized to an internal control, β-actin. The cropped images of immunoblots displayed in the figure and the full-length blots were shown in Supplementary Fig. S11. Data are expressed as mean ± SE values *P < 0.05.

**Figure 2:**
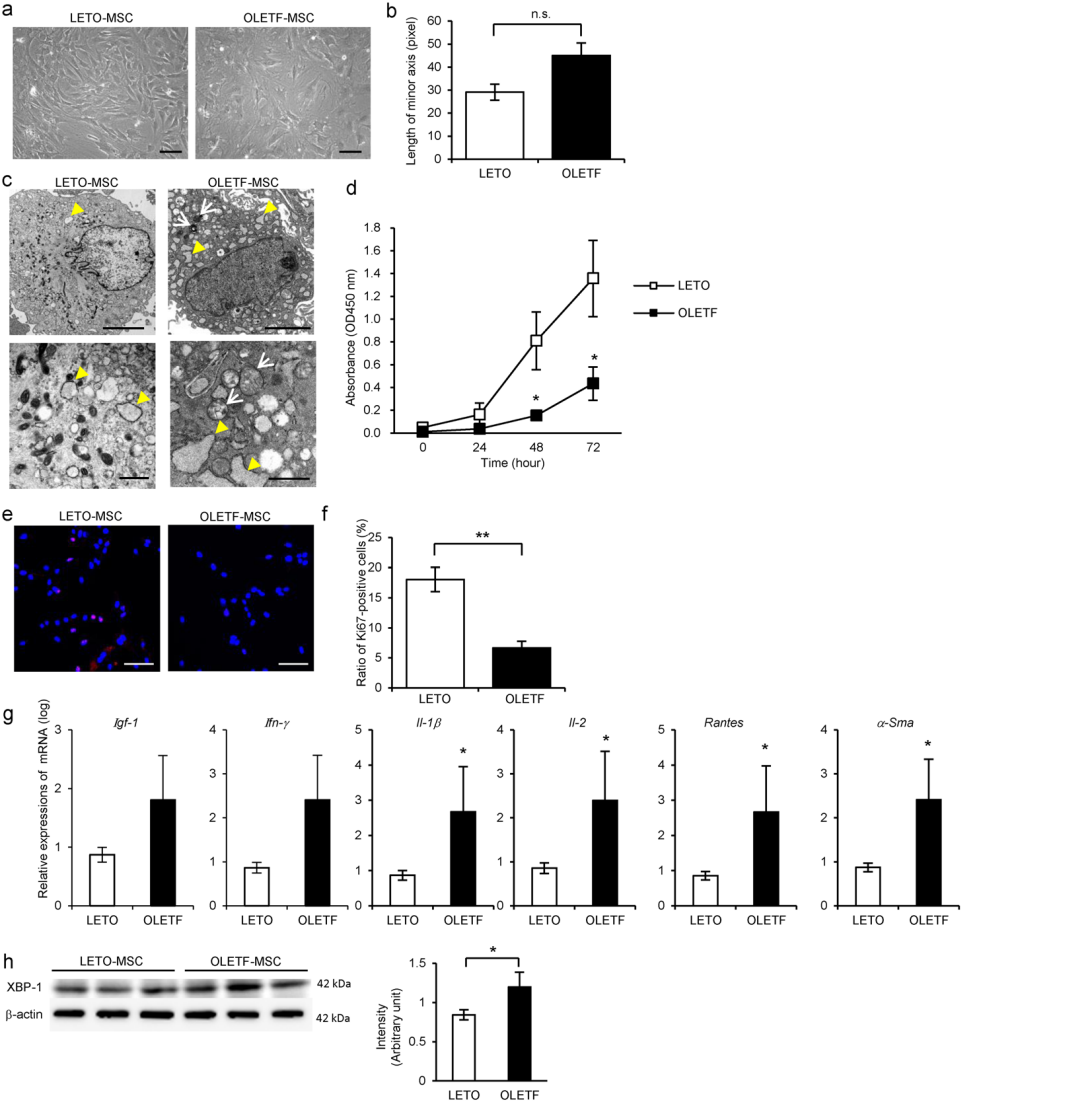
Abnormalities of BM-MSC isolated from Otsuka Long-Evans Tokushima Fatty (OLETF) diabetic rats (OLETF-MSC) compared with Long-Evans Tokushima Otsuka (LETO) rats (LETO-MSC). (**a**) Phase contrast images of LETO-MSC and OLETF-MSC (passage 3). Bar: 100 µm. (**b**) Quantitative analysis morphological findings of BM-MSC. The lengths of minor axis in phase contrast images were measured in all cells in five panels of each BM-MSC isolated from individual animals. Values are means ± SE of the LETO-MSC (n = 3) and OLETF-MSC (n = 4). *P* = 0.0771 (not significant). (**c**) TEM images of BM-MSC. Upper panels show the entire cell at low magnification; lower panels show cell organelles at high magnification. White arrows show mitochondria degenerations. Yellow arrowheads show abnormal dilation of ER. Bar: 5 µm in upper panels and 500 nm in lower panels. (**d**) MTT proliferation assays of LETO-MSC and OLETF-MSC. Absorbance at 450 nm was measured 0, 24, 48 and 72 hours after MTT addition. Values are means ± SE of the LETO (n = 4) and OLETF (n = 4). *P < 0.05. (**e**) Immunofluorescence staining of BM-MSC with anti-Ki-67 antibody (red). DAPI was used for counterstaining nuclei (blue). Bar: 100 µm. (**f**) The average ratio of Ki-67-positive cells to the total cell count. The number of Ki-67-positive cells and nuclei was counted in five panels of each BM-MSC isolated from individual animals. Values are means ± SE of the LETO (n = 4) and OLETF (n = 4). **P < 0.01. (**g**) Relative expressions of mRNA in BM-MSC. Values are means ± SE of the LETO (n = 4) and OLETF (n = 3). *P < 0.05. (**h**) Western blot analysis of LETO-MSC (n = 3) and OLETF-MSC (n = 3) using anti-XBP-1 and β-actin antibodies. Relative amounts of protein are normalized to an internal control, β-actin. The cropped images of immunoblots displayed in the figure and the full-length blots were shown in Supplementary Fig. S12. Data are expressed as mean ± SE values *P < 0.05.

Morphological findings of STZ-MSC and OLETF-MSC were abnormal, with short blunt edge cell projections, enlarged individual cell area, flat shape with increased actin fibers in the cytoplasm compared with Control-MSC and Long-Evans Tokushima Otsuka (LETO)-MSC respectively (Figs 1a and 2a). We adopted the length of the minor axis through the cell nuclei to quantify cell morphology instead of measuring the cell area directly. The average length of the minor axis of cells was significantly longer in STZ-MSC than that of Control-MSC (*P* = 0.0324, Fig. 1b). A similar trend was observed in OLETF-MSC compared with LETO-MSC. The average length of the minor axis of cells was longer in OLETF-MSC than that of LETO-MSC (*P* = 0.0771, Fig. 2b). Ultrastructural abnormalities were observed in the cytoplasm of both STZ-MSC and OLETF-MSC by transmission electron microscopy (TEM). Large numbers of degenerated mitochondria and marked expansion of endoplasmic reticulum (ER) were prominent (Figs 1c and 2c). Although ER expansion was observed partially in LETO-MSC, the abnormality was more severe in OLETF-MSC.

Proliferation of STZ-MSC and OLETF-MSC was significantly reduced compared with Control-MSC and LETO-MSC, respectively. Cell growth indicated by the MTT proliferation assay was reduced markedly in STZ-MSC (*P* *=* 0.0467 at 72 hours, Fig. 1d) and OLETF-MSC (*P* = 0.0435 at 48 hours, *P* = 0.0447 at 72 hours, Fig. 2d). Proliferative ability, as indicated by the ratio of Ki-67 positive cells to total cells, was significantly decreased in STZ-MSC (Fig. 1e; *P* < 0.001, Fig. 1f) and OLETF-MSC (Fig. 2e; *P* = 0.0026, Fig. 2f).

### Gene expression of growth factor, actin stress fiber and cytotoxic cytokines/chemokines were abnormal in STZ-MSC and OLETF-MSC

The relative mRNA expression of insulin-like growth factor-1 (*Igf-1*) was downregulated, whereas interferon-gamma (*Ifn-γ*), interleukin-1 beta (*Il-1β*), interleukin-2 (*Il-2*) and regulated on activation normal T cell expressed and secreted (*Rantes*) were upregulated in STZ-MSC compared with Control-MSC (*P* < 0.05, Fig. 1g). In contrast, relative mRNA expressions of alpha smooth muscle actin (*α-Sma*), which represents the stress fiber of MSCs, *Il-1β*, *Il-2* and *Rantes* were upregulated in OLETF-MSC compared with LETO-MSC (*P* < 0.05, Fig. 2g).

### ER stress was increased in STZ-MSC and OLETF-MSC

Expression of XBP-1 (splicing) was increased in STZ-MSC and OLETF-MSC compared with Control-MSC and LETO-MSC respectively (*P* < 0.05, Figs 1h and 2h). These results reflect the response to marked ER stress as previously shown by TEM.

### DM-MSC did not ameliorate renal injury in STZ-induced diabetic mice

**Figure 3:**
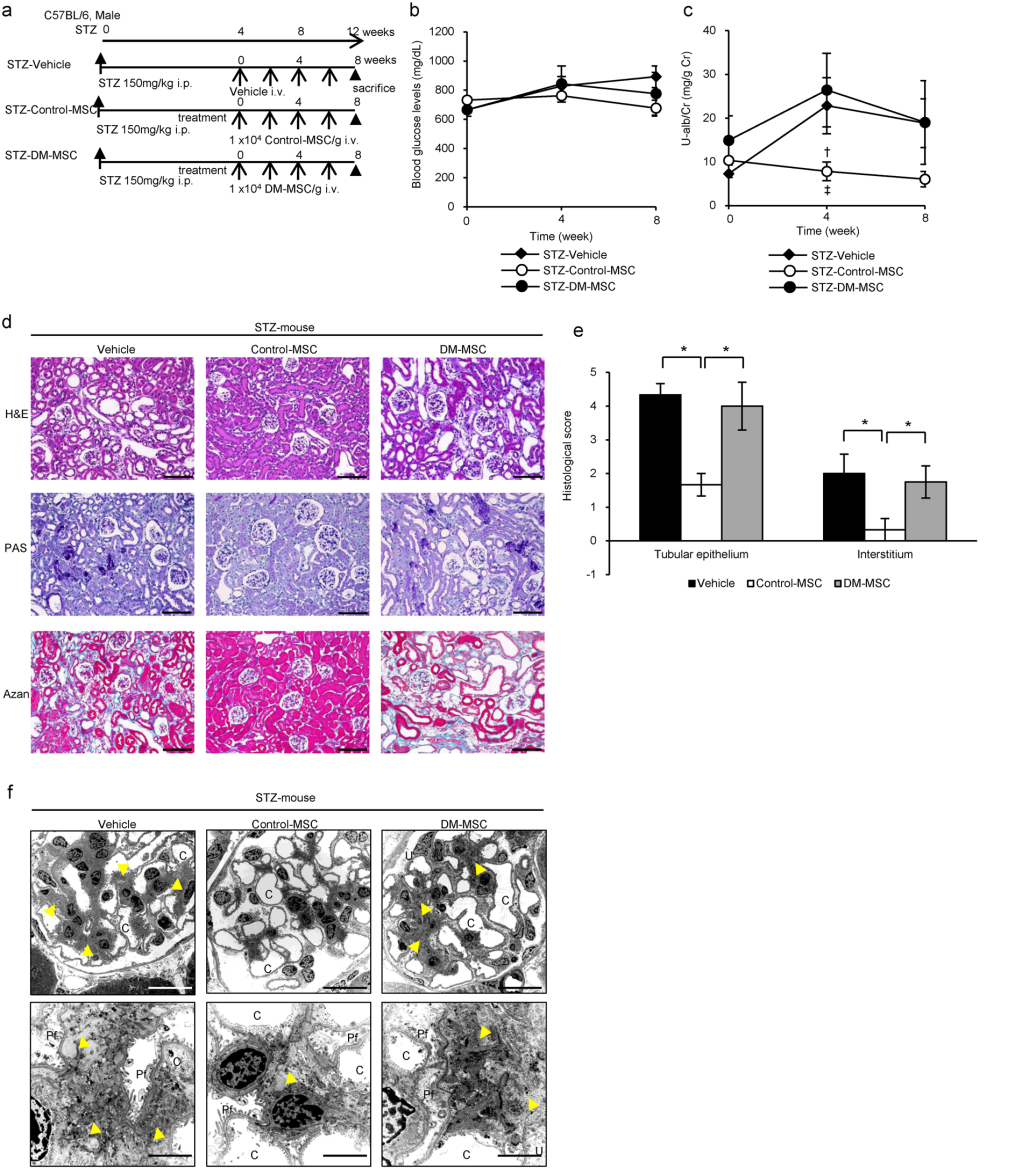
Therapeutic effect of BM-MSC isolated from diabetic rats (DM-MSC) for diabetic nephropathy in STZ-induced diabetic mice. (**a**) Experimental protocol for BM-MSC therapies in streptozotocin (STZ)-induced diabetic mice. (**b**) Changes in blood glucose levels after initial BM-MSC administration. Values are expressed as mean ± SE of 3–5 animals. (**c**) Changes of urine albumin/creatinine ratio (U-alb/Cr) after initial BM-MSC administration. Values are expressed as mean ± SE of 3–5 animals. ^†^ *P* *<* 0.05 STZ-Control-MSC vs. STZ-Vehicle; ^‡^ *P* *<* 0.05 STZ-Control-MSC vs. STZ-DM-MSC. (**d**) Histological findings of the renal cortex in H&E-, PAS, and Azan staining kidney sections at 8 weeks after the initial administration of BM-MSC in STZ-induced diabetic mice. Bar: 100 µm. (**e**) Quantification of tubular epithelial damage and interstitial change in STZ-Vehicle, STZ-Control-MSC and STZ-DM-MSC mice. Data shown are representative of five panels per animal at ×100 magnification. Data are expressed as mean ± SE of 3–4 animals. *P *<* 0.05. (**f**) Ultrastructural TEM analysis of the renal glomerulus in STZ-induced diabetic mice 8 weeks after initial administration of BM-MSC. C, glomerular capillary; Pf, podocyte foot process; Yellow arrowhead, mesangial matrixes. Bar: 20 µm in upper panels, 2 µm in lower panels.

The experimental protocol for BM-MSC therapy in STZ-diabetic mice is shown (Fig. 3a). First, we compared blood glucose levels and urinary albumin-creatinine ratio (U-alb/Cr) in mice administered a single high dose (150 mg/kg) of STZ and mice administered five low doses (40 mg/kg) of STZ (Fig. 3b,c; Supplementary Fig. S3a,b). Because there was no difference in U-alb/Cr between the two groups, we used the single high-dose administration model in this study. We have previously established a protocol to treat STZ-induced diabetic mice with multiple intravenous administration of rat BM-MSC, therefore we first demonstrated the diabetes-associated abnormality of rat BM-MSC using this model. Blood glucose levels did not decrease in any of the groups (Fig. 3b), and pancreatic islet tissues size and insulin expression levels did not improve (Supplementary Fig. S3c). We initiated the treatment when albuminuria was well developed. The value of U-alb/Cr was 7.28 ± 0.33 mg/g Cr (means ± SE) in STZ-Vehicle mice (n = 5), whereas it was 0.80 ± 0.29 mg/g Cr in control mice (n = 5). Administration of Control-MSC inhibited the progression of renal injury, whereas DM-MSC derived from STZ rats exacerbated renal injury, as indicated by U-alb/Cr levels. This was consistent with results for STZ-induced diabetic mice treated with vehicle (STZ-Vehicle mice) (Fig. 3c).

Histological findings of the kidney were observed. Abnormal dilatation of the renal tubules, collective apoptosis in the tubular units, massive accumulation of inflammatory cells and fibrotic changes in the interstitial area of the renal cortex were observed in STZ-Vehicle mice with hematoxylin and eosin (H&E), periodic acid-Schiff (PAS), and Azan staining (Fig. 3d, left panels). Acute tubular necrosis (ATN), previously reported responses to STZ toxicity, was not observed by histology in early phase STZ mice, that is 5 weeks after STZ administration (Supplementary Fig. S4e, middle panels). Administration of Control-MSC suppressed histological damages in the kidney, whereas DM-MSC could not improve the damage but rather exacerbated it (Fig. 3d). The quantitative value from the histological scoring system is shown in Fig. 3e. Ultrastructural abnormalities were observed by TEM in the glomerulus in STZ-diabetic mice. An expansion of glomerular mesangial matrix and mild swelling of podocyte foot processes were observed in STZ-Vehicle mice (Fig. 3f). Administration of Control-MSC suppressed the expansion of glomerular mesangial matrix, whereas DM-MSC did not suppress this expansion (Fig. 3f). There was no obvious change in the podocyte foot processes between STZ-Control-MSC mice and STZ-DM-MSC mice.

### Characteristics of WJs

**Figure 4:**
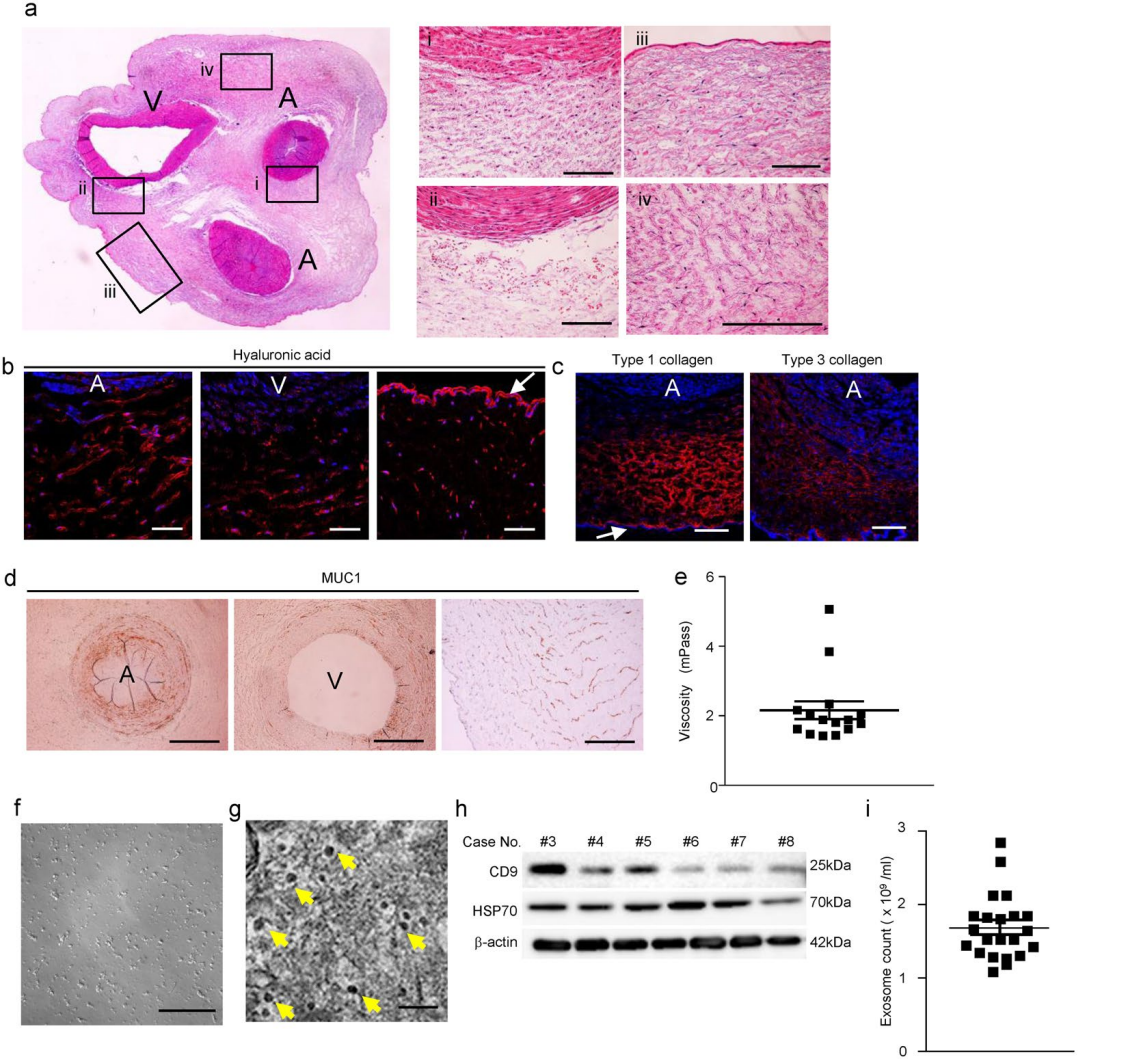
Contents of Wharton’s jelly extract supernatant (WJs). (**a**) Histological findings of cross-section of umbilical cord (UC) with H&E staining. A, umbilical artery; V, umbilical vein. Right panels show magnified images of the wall of the umbilical artery (i), umbilical vein (ii), amnion epithelium and sub-amniotic tissue (iii), and Wharton’s jelly and mesenchymal tissue of UC (iv). Bar: 100 µm. (**b**) Immunofluorescence staining of hyaluronic acid (red) in UC tissues. DAPI was used for counterstaining of nuclei (blue). Left panel shows the wall of umbilical artery and peri-arterial tissues. Middle panel shows the wall of umbilical vein and peri-venous tissues. Right panel shows the amniotic membrane and sub-amniotic tissues. White arrow; amniotic membrane. Bar: 200 µm. (**c**) Immunofluorescence staining of type 1 collagen (left panel) and type 3 collagen (right panel) (red) of peri-arterial tissues in UC. DAPI was used for counterstaining nuclei (blue). Bar: 200 µm. (**d**) Immunohistochemical staining of Mucin 1 (MUC1) with DAB method in UC tissues. Left panel shows umbilical artery. Middle panel shows umbilical vein. Right panel shows the Wharton’s jelly and mesenchymal tissue of UC. Bar: 500 µm. (**e**) The viscosity of WJs extracted from UC tissues. Values are expressed as mean ± SE of 15 fetuses. (**f**) Phase contrast images of WJs after 96 hours of culture at 37 °C by the addition of FBS. Bar: 100 µm. (**g**) TEM images of pellets of WJs. Yellow arrow shows 30 – 50 µm of the size of microvesicles, which are recognized as exosomes, in the pellets. Bar: 100 nm. (**h**) Western blot analysis of WJs-derived exosomes obtained from six fetuses using anti-CD9, anti-HSP70 and β-actin antibodies. The cropped images of immunoblots displayed in the figure and the full-length blots were shown in Supplementary Fig. S13. (**i**) The number of exosomes in each lot of WJs were quantified by CD9 ELISA. Values are expressed as mean ± SE of 22 fetuses.

Histological findings of UC were observed initially. UC consists with two umbilical arteries, one umbilical vein, amniotic membrane and Wharton’s jelly as a stromal component (Fig. 4a–i, ii, iii and iv). Large numbers of cellular components with hematoxylin-positive nuclei and loose connective tissue were present in the stroma (Fig. 4a–iv). Hyaluronic acid, which is the most common component of extracellular matrix in UC^27^, existed abundantly in the amniotic membrane and stromal cells in UC (Fig. 4b). Type 1 collagen was present in the stromal tissue and amniotic membrane, whereas Type 3 collagen was expressed in the stromal tissue and the wall of UC vessel (Fig. 4c). Mucin 1 (MUC1), which is one of the components responsible for the high viscosity, was expressed in the stromal tissue and the wall of UC vessel (Fig. 4d).

Then, WJs were obtained from whole UC by the procedure described in the Methods. The average viscosity of WJs obtained from UC of 15 donors was 2.16 ± 0.26 mPass (Fig. 4e). Large variation was not observed except for in two samples. To confirm that WJs did not include viable cells, WJs were monitored for 96 hours by culturing with 10% FBS at 37 °C and 5% CO_2_. No proliferative cells were detected by phase contrast images (Fig. 4f). Abundant 30–50-µm microvesicles were observed in the pellets of WJs by TEM, which were recognized as exosomes (Fig. 4g). Cluster of differentiation (CD) 9 and heat shock protein (HSP) 70, specific markers for exosomes, were detected in the pellets of microvesicles isolated from WJs (Fig. 4h). Average number of exosomes contained in WJs obtained from UC of 22 donors was 1.68 ± 0.09 × 10^9^ exosomes/ml (Fig. 4i). Large variations were not observed except for in two samples.

Other components which might be nutrients for cells were measured. The content of growth factors, extracellular matrixes and L-Glutamate in WJs were as follows. Insulin-like growth factor-1 (IGF-1), 93.496 ± 1.960 pg/ml (n = 16); Epidermal growth factor (EGF), 13.478 ± 1.547 pg/ml (n = 16); platelet-derived growth factor-AB (PDGF-AB), 7.668 ± 1.118 pg/ml (n = 16); basic fibroblast growth factor (b-FGF), 6.728 ± 0.435 mg/ml (n = 16); hyaluronic acid, 161.610 ± 8.009 ng/ml (n = 8); L-Glutamate, 76.205 ± 5.013 mg/ml (n = 20). Data are expressed as mean ± SE.

### WJs improved morphology, proliferative ability and cell mobilization of DM-MSC

**Figure 5:**
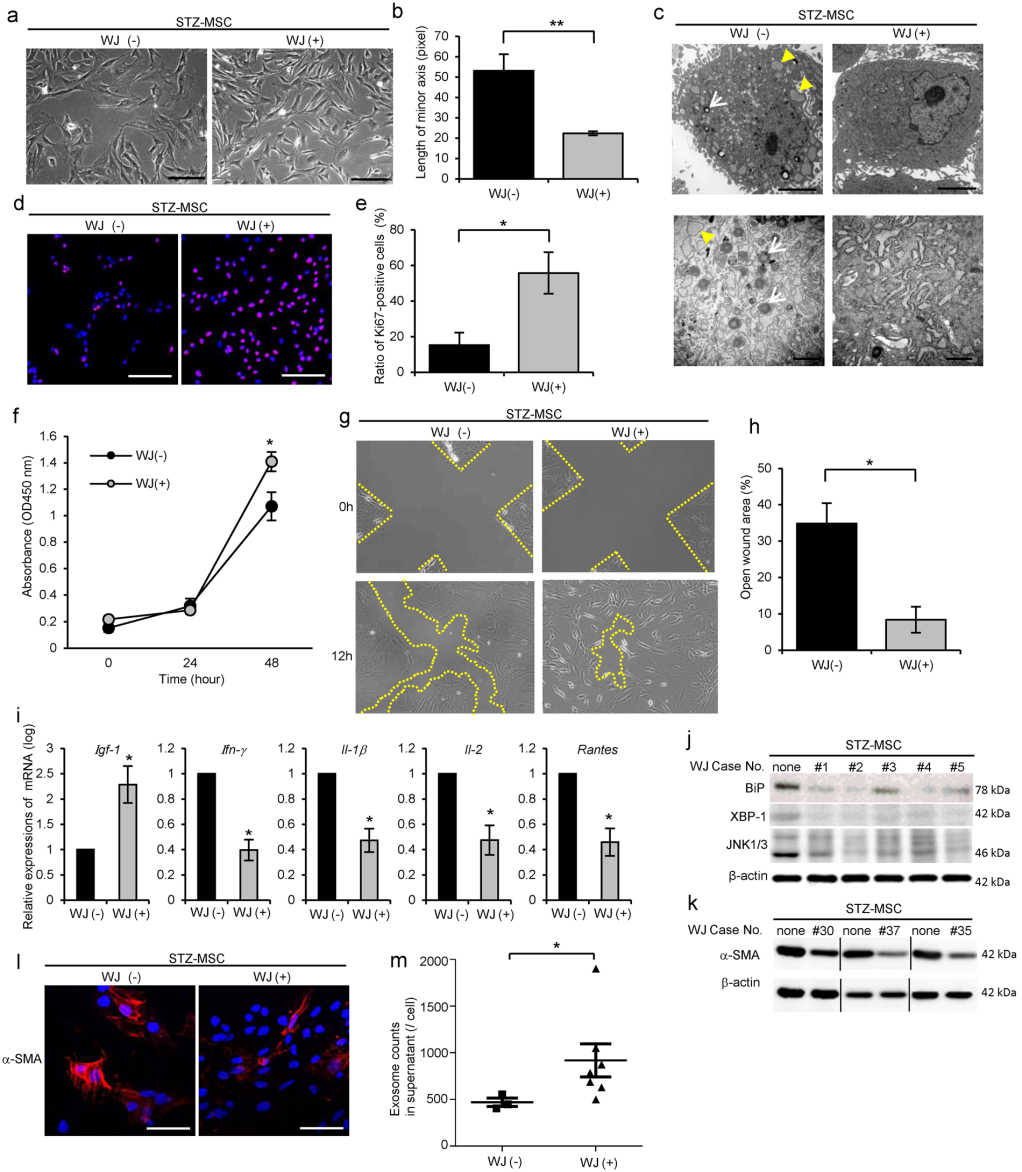
Activating effects of WJs for STZ-MSC. (**a**) Phase contrast images of STZ-MSC. Bar: 100 µm. (**b**) The average length of minor axis of cells in phase contrast images. All cells in five panels of each STZ-MSC were measured. N = 4, each. **P *<* 0.01. (**c**) TEM images of STZ-MSC. White arrows show mitochondrial degeneration. Yellow arrowheads show abnormal dilation of the ER. Bar: 5 µm in upper panels; 500 nm in lower panels. (**d**) Immunofluorescence staining of STZ-MSC with Ki-67 antibody (red). Nuclei counterstained with DAPI (blue). Bar: 100 µm. (**e**) Average ratio of Ki-67-positive cells to total cell count in five panels of each STZ-MSC. N = 3, each. *P *<* 0.05. (**f**) MTT proliferation assays of STZ-MSC. Absorbance was measured 0, 24 and 48 hours after MTT addition. WJ(−); n = 7, WJ(+); n = 29. *P *<* 0.05. (**g**) STZ-MSC scratch assay. The open wound area was measured immediately and 12 hours after performing the cross-scratch. Yellow lines indicate the cell mobilization edge. (**h**) The ratio of open wound area at 12 hours compared with 0 hours. Five cross-scratch points in each STZ-MSC were measured. N = 3, each. *P *<* 0.05. (**i**) Relative expressions of mRNA in STZ-MSC. WJ(−); n = 4, WJ(+); n = 12. *P *<* 0.05. (**j**) Western blot analysis of STZ-MSC cultured with 5 lots of WJs (#1–5) or without WJs using anti-BiP, anti-XBP-1, anti-JNK1/3 and β-actin antibodies. The cropped images of immunoblots displayed in the figure and the full-length blots were shown in Supplementary Fig. S14. (k) Western blot analysis of STZ-MSC cultured with 3 lots of WJs (#30, 37, 35) or without WJs using anti-α-SMA and β-actin antibodies. The cropped images of immunoblots displayed in the figure and the full-length blots were shown in Supplementary Fig. S15. (**l**) Immunofluorescence staining of STZ-MSC with anti-α-SMA antibody (red). Nuclei counterstained with DAPI (blue). Bar: 100 µm. (**m**) The exosome count in the STZ-MSC supernatant quantified by CD9 ELISA. WJ(−); n = 3, WJ(+); n = 7. *P *<* 0.05. Values are expressed a mean ± SE.

**Figure 6:**
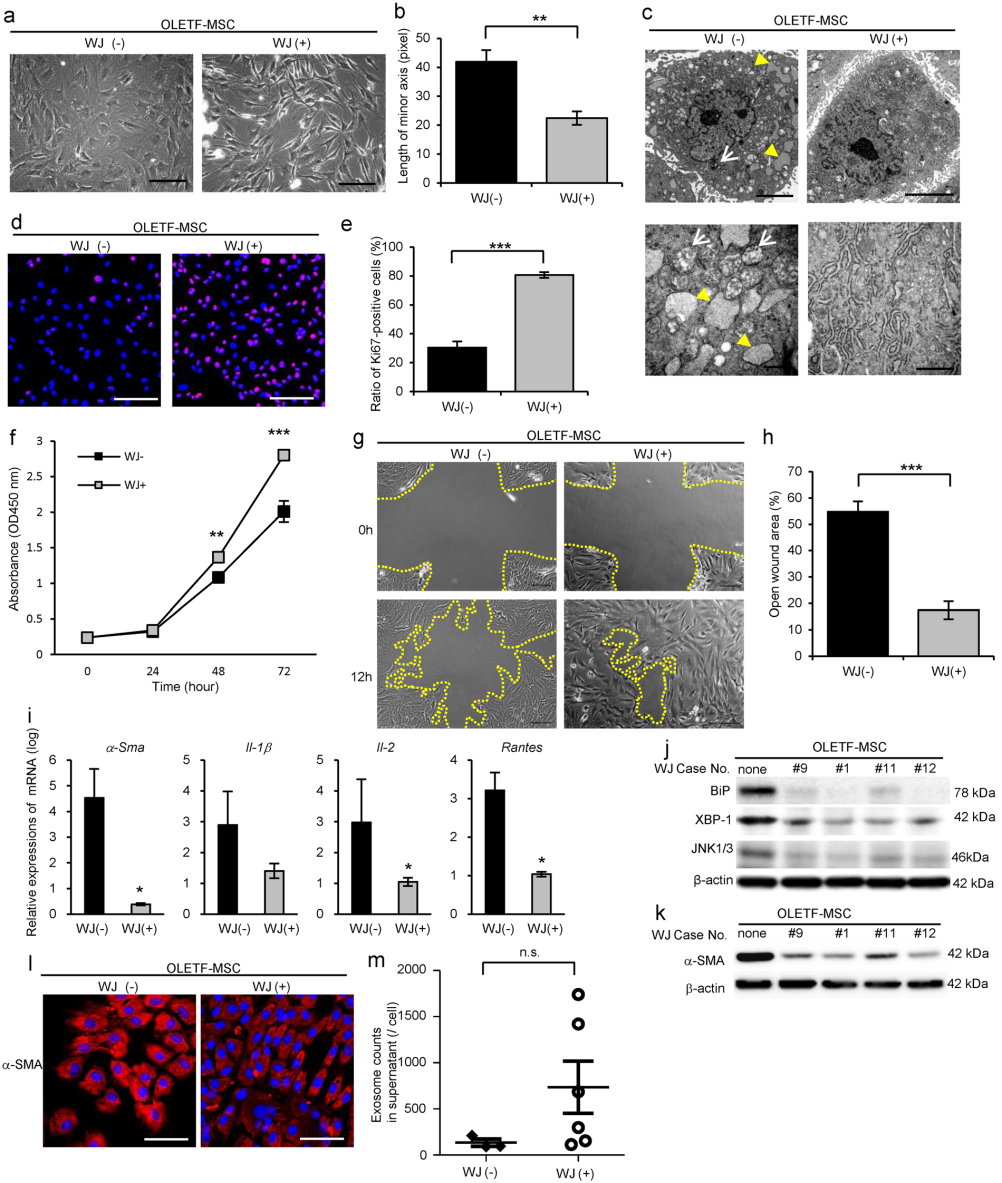
Activating effects of WJs for OLETF-MSC. (**a**) Phase contrast images of OLETF-MSC. Bar: 100 µm. (**b**) The average length of the minor axis of cells in phase contrast images were measured in all cells in five panels of each OLETF-MSC. N = 5, each. **P *<* 0.01. (**c**) TEM images of OLETF-MSC. White arrows show mitochondria degenerations. Yellow arrowheads show abnormal dilation of the ER. Bar: 5 µm in upper panels and 500 nm in lower panels. (**d**) Immunofluorescence staining of OLETF-MSC with Ki-67 antibody (red). Nuclei counterstained with DAPI (blue). Bar: 100 µm. (**e**) The average ratio of Ki-67-positive cells to total cell count in five panels of each OLETF-MSC. N = 3, each. ***P *<* 0.001. (**f**) MTT proliferation assays of OLETF-MSC. Absorbance at 450 nm was measured 0, 24, 48 and 72 hours after the MTT addition. WJ(−); n = 5, WJ(+); n = 15. **P < 0.01, ***P *<* 0.001. (**g**) Scratch assay of OLETF-MSC. The open wound area was measured immediately and 12 hours after performing the cross-scratch. Yellow lines indicate the cell mobilization edge. (**h**) Quantitative analysis of the ratio of open area at 12 hours compared with 0 hours, measured in five cross-scratch points in each OLETF-MSC. N = 3, each. ***P *<* 0.001. (**i**) Relative expressions of mRNA in OLETF-MSC. WJ(−); n = 4, WJ(+); n = 10. *P *<* 0.05. (**j**) Western blot analysis of OLETF-MSC which are cultured with WJs (lots #9, #1, #11, #12) or without WJs using anti-BiP, anti-XBP-1, anti-JNK1/3 and β-actin antibodies. The cropped images of immunoblots displayed in the figure and the full-length blots were shown in Supplementary Fig. S16. (**k**) Western blot analysis of OLETF-MSC cultured with WJs (lots #9, #1, #11, #12) or without WJs using anti-α-SMA and β-actin antibodies. The cropped images of immunoblots displayed in the figure and the full-length blots were shown in Supplementary Fig. S17. (**l**) Immunofluorescence staining of OLETF-MSC with anti-α-SMA antibody (red). Nuclei counterstained with DAPI (blue). Bar: 100 µm. (**m**) The number of exosomes in the supernatant of OLETF-MSC quantified by CD9 ELISA. WJ(−); n = 3, WJ(+); n = 6. Values are expressed a mean ± SE.

Under the phase contrast microscope, STZ-MSC and OLETF-MSC cultured with WJs changed into thicker, spindle-shaped cells, and the projections of the cells were thinner and longer (Figs 5a and 6a). Cell area represented by the length of the short-axis of cells was markedly decreased (*P* = 0.0095, Fig. 5b; *P* = 0.0031, Fig. 6b). These effects were enhanced in a concentration-dependent manner with WJs (Supplementary Figs S5a and S6a). Mitochondrial degeneration and abnormal expansion of ER was improved in STZ-MSC and OLETF-MSC by culturing with WJs by TEM observations (Figs 5c and 6c).

The proliferative ability of STZ-MSC and OLETF-MSC cultured with WJs (STZ-MSC-WJ(+) and OLETF-MSC-WJ(+)) was improved significantly as indicated by the Ki-67 labeling index (Fig. 5d; *P* = 0.0409, Fig. 5e and Fig. 6d; *P* = 0.0005, Fig. 6e) and MTT proliferative assay (*P* = 0.0402, Fig. 5f; *P* = 0.0023 at 48 hours and *P* < 0.0001 at 72 hours, Fig. 6f). Cell mobilization was markedly up-regulated in STZ-MSC-WJ(+) and OLETF-MSC-WJ(+) as assessed by scratch assay (Figs 5g and 6g). The open wound area was significantly decreased in STZ-MSC-WJ(+) and OLETF-MSC-WJ(+)12 hours after creating the wound (*P* = 0.0163, Fig. 5h; *P* < 0.0001, Fig. 6h).

### WJs improved expression of growth factors and cytotoxic cytokines/chemokines by DM-MSC

Relative mRNA expression of *Igf-1* was recovered, whereas *Ifn-γ*, *Il-1β*, *Il-2* and *Rantes* were suppressed in STZ-MSC cultured with WJs (STZ-MSC-WJ(+)) compared with STZ-MSC cultured without WJs (STZ-MSC-WJ(−)) (*P* < 0.05, Fig. 5i). Similarly, the relative mRNA expressions of *α-Sma*, *Il-1β*, *Il-2* and *Rantes* were suppressed in OLETF-MSC cultured with WJs (OLETF-MSC-WJ(+)) compared with OLETF-MSC cultured without WJs (OLETF-MSC-WJ(−)) (*P* < 0.05, Fig. 6i).

### WJs improved ER stress and suppressed actin stress fibers in STZ-MSC and OLETF-MSC

High expression of BiP, XBP-1 (splicing) and JNK1/3, which indicated marked ER stress, was suppressed clearly in STZ-MSC and OLETF-MSC by culturing with WJs (Figs 5j and 6j). WJs changed the expression of ER stress markers similar to ER stress inhibitor, 4-phenylbutyrate (PBA) (Supplementary Figs S5b and S6b). These results were consistent with ultrastructural findings by TEM. Alpha-SMA which was overexpressed in the cytoplasm of STZ-MSC and OLETF-MSC was suppressed by culture with WJs (Fig. 5k,l and Fig. 6k,l). These effects were enhanced in a concentration-dependent and time-dependent manner of exposure to WJs in STZ-MSC-WJ (Supplementary Fig. S5d,e) and OLETF-MSC-WJ (Supplementary Fig. S6c,d).

### Exosome secretion by DM-MSC was increased by WJs

The therapeutic potential of BM-MSC in nephropathy has been reported to be largely mediated by paracrine factors including exosomes which were released from intracellular endosomes^28^. Therefore, we investigated the change of exosome secretion ability as one of the functional improvements in DM-MSC. The number of exosome secreted into the supernatant by the STZ-MSC was significantly increased by culturing with WJs (*P* *=* 0.0333, Fig. 5m). A similar tendency was observed in OLETF-MSC (*P* *=* 0.0952, Fig. 6m).

### Exosomes contained in WJs were the key factor activating DM-MSC

**Figure 7:**
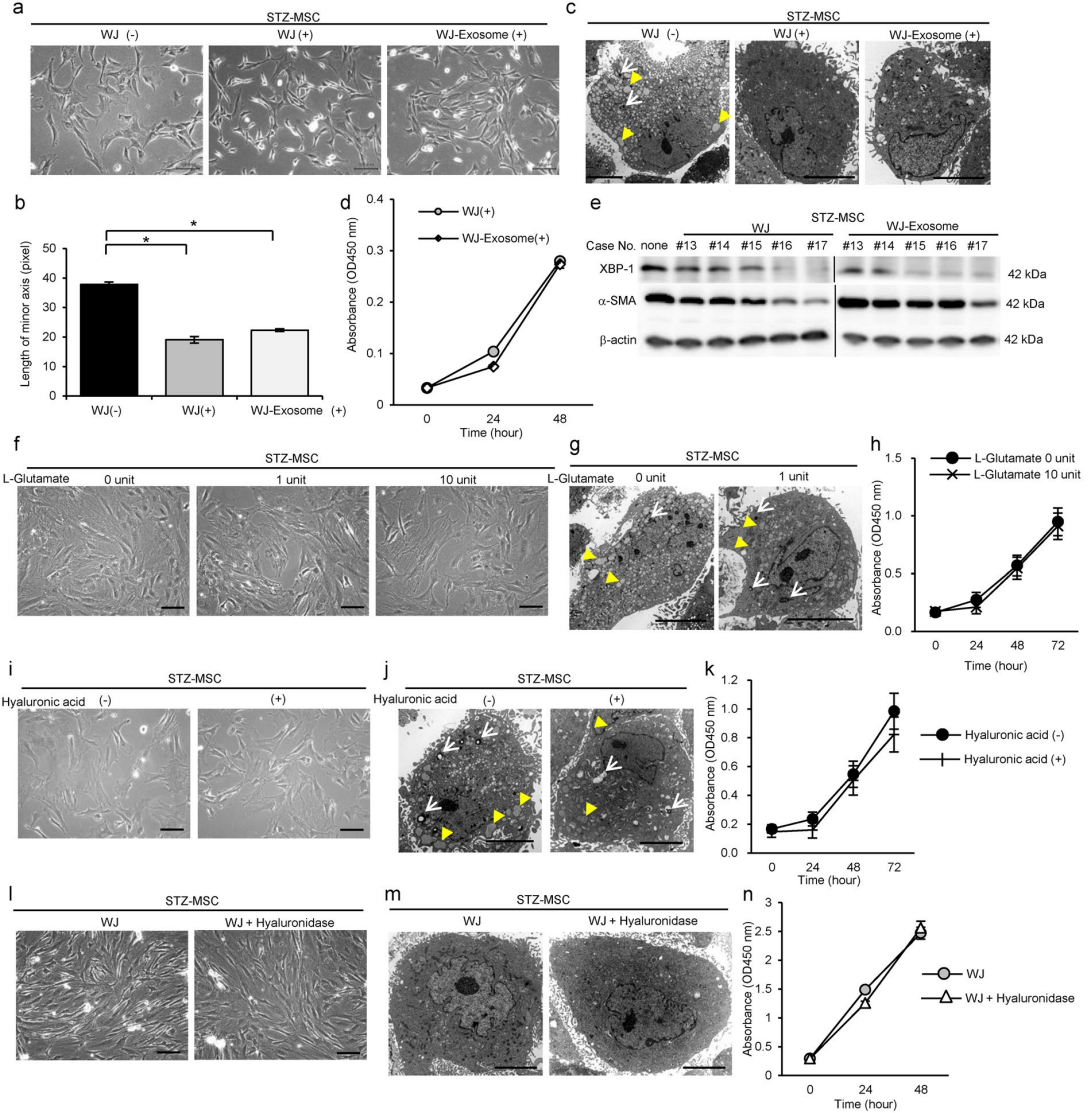
Activating effects of exosomes isolated from WJs (WJ-Exosome), L-Glutamate and hyaluronic acid compared with complete WJs for STZ-MSC. (**a**) Phase contrast images of STZ-MSC cultured with complete WJs or WJ-Exosome. Bar: 100 µm. (**b**) The average length of minor axis of cells in phase contrast images. All cells in five panels of each STZ-MSC were measured. N = 3, each. *P < 0.05. (**c**) TEM images of STZ-MSC. White arrows show mitochondria degenerations. Yellow arrowheads show abnormal dilation of the ER. Bar: 5 µm. (**d**) MTT proliferation assays of STZ-MSC. Absorbance was measured 0, 24 and 48 hours after the MTT addition. N = 5, each. (**e**) Western blot analysis of STZ-MSC cultured with complete WJs (lots #13–17) or WJ-exosome (same lots) using anti-α-SMA and β-actin antibodies. The cropped images of immunoblots displayed in the figure and the full-length blots were shown in Supplementary Fig. S18. (**f**) Phase contrast images of STZ-MSC cultured with L-Glutamate. One unit of L-Glutamate indicates the same amount of L-Glutamate included in complete WJs which are added to STZ-MSC. Bar: 100 µm. (**g**) TEM images of STZ-MSC cultured with L-Glutamate. White arrows show mitochondria degenerations. Yellow arrowheads show abnormal dilation of the ER. Bar: 5 µm. (**h**) MTT proliferation assays of STZ-MSC. Absorbance was measured 0, 24, 48 and 72 hours after the MTT addition. N = 5, each. (**i**) Phase contrast images of STZ-MSC cultured with hyaluronic acid. Bar: 100 µm. (**j**) TEM images of STZ-MSC cultured with hyaluronic acid. White arrows show mitochondria degeneration. Yellow arrowheads show abnormal dilation of the ER. Bar: 5 µm. (**k**) MTT proliferation assays of STZ-MSC. Absorbance was measured 0, 24, 48 and 72 hours after the MTT addition. N = 5, each. (**l**) Phase contrast images of STZ-MSC cultured with WJs and WJs pretreated with hyaluronidase. Bar: 100 µm. (**m**) TEM images of STZ-MSC. Bar: 5 µm. (**n**) MTT proliferation assays of STZ-MSC. Absorbance was measured 0, 24 and 48 hours after the MTT addition. N = 5, each. Values are expressed a mean ± SE.

To investigate the major components of WJs activating DM-MSC, we focused on the exosomes contained in WJs. The morphology of STZ-MSC changed to thicker and spindle-shape by exosomes isolated from WJs (WJ-exosome), similar to complete WJs (WJ(+)) (Fig. 7a). The length of the minor axis of cells was markedly decreased in both WJs and WJ-exosome treated cells (*P* < 0.05, WJ(+) vs. WJ(−); *P* < 0.05, WJ-exosome(+) vs. WJ(−); Fig. 7b). Abnormal expansion of ER was clearly improved in both WJs and WJ-exosome treated cells, whereas autophagosomes remained partially in WJ-exosome treated cells in TEM observation (Fig. 7c). Proliferative ability of STZ-MSC cultured with WJ-exosome was similar to that of WJs by MTT proliferative assay (Fig. 7d). WJ-exosome suppressed high expression of XBP-1 in STZ-MSC, similar to WJs, whereas the suppressive effect of WJ-exosome for the expression of α-SMA was slightly weaker than WJs (Fig. 7e).

We also investigated the activating effects of L-Glutamate and hyaluronic acid which were other candidate factors in WJs to activate DM-MSC. However, morphological findings and cell proliferation of STZ-MSC was not altered by the addition of 1 unit of L-Glutamate, which was equivalent amount of L-Glutamate contained in complete WJs to be added to cells, and even by 10 units (Fig. 7f,g,h). Similarly, the addition of hyaluronic acid into the culture medium did not show the activation effect on STZ-MSC (Fig. 7i,j,k). In order to confirm the absence of the effect of hyaluronic acid, we prepared the WJs which were pretreated with hyaluronidase to degrade hyaluronic acid in WJs enzymatically. This modified WJs still exhibited the activation effect of STZ-MSC. Morphological findings and proliferation ability of STZ-MSC which was cultured with modified WJs were similar to that of complete WJs which were not pretreated with hyaluronidase (Fig. 7l,m,n).

### DM-MSC activated by WJs (DM-MSC-WJ) ameliorated renal injury in diabetic rats

**Figure 8:**
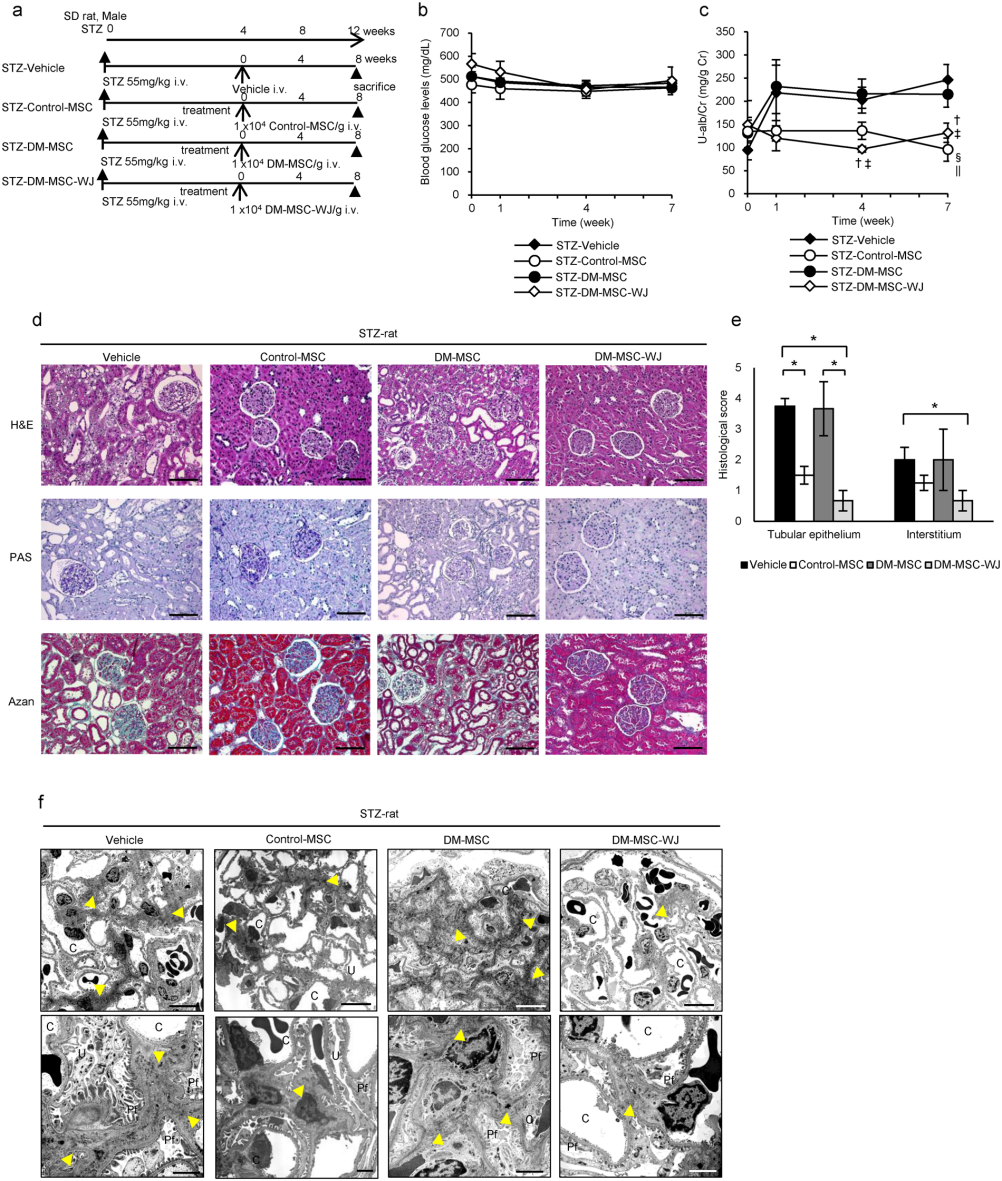
Therapeutic effect of DM-MSC which are activated by WJs for nephropathy in STZ-induced diabetic rats. (**a**) Experimental protocol for MSC therapies in STZ-diabetic rats. (**b**) Changes in blood glucose levels after initial BM-MSC administration. Values are expressed as mean ± SE of 4–6 animals. (**c**) Changes of U-alb/Cr after the administration of BM-MSC. Values are expressed as mean ± SE of 4–6 animals. ^†^ *P* < 0.05 STZ-DM-MSC-WJ vs. STZ-Vehicle; ^‡^ *P* *<* 0.05 STZ- DM-MSC-WJ vs. STZ-DM-MSC; ^§^ *P* *<* 0.05 Control-MSC vs. STZ-Vehicle; ^||^ *P* *<* 0.05 Control-MSC vs. STZ-DM-MSC. (**d**) Histological findings of the renal cortex in H&E, PAS, and Azan staining kidney sections at 8 weeks after the administration of BM-MSC in STZ-diabetic rats. Bar: 100 µm. (**e**) Quantification of tubular epithelial damage and interstitial change in STZ-Vehicle, Control-MSC, STZ-DM-MSC and STZ-DM-MSC-WJ rats. Data shown are representative of five panels per animal at × 100 magnification. Data are expressed as mean ± SE of 3–4 animals. *P *<* 0.05. (**f**) Ultrastructural TEM analysis of the renal glomerulus in STZ-induced diabetic rats 8 weeks after initial administration of BM-MSC. C, glomerular capillary; Pf, podocyte foot process; Yellow arrowhead, mesangial matrixes. Bar: 10 µm in upper panels, 2 µm in lower panels.

The experimental protocol for BM-MSC therapies in STZ rats is shown (Fig. 8a). To investigate the efficacy of DM-MSC-WJ on DN in a manner more analogous to autologous transplantation in humans, we used STZ rats treated with rat BM-MSC to evaluate the effectiveness of the treatment in the donor species. The mortality rate of STZ diabetic rats was 11.1% (n = 18) in the 30 weeks after administration of STZ. Blood glucose levels did not change in any of groups (Fig. 8b), and pancreatic islet tissue size and insulin expression levels did not increase (Supplementary Fig. S7). We initiated the treatment when albuminuria was well developed. The value of U-alb/Cr was 93.82 ± 20.45 mg/g Cr (means ± SE, n = 3-5) in STZ-Vehicle rats, whereas it was 14.73 ± 5.45 mg/g Cr in control rats before each treatment. Although administration of DM-MSC exacerbated U-alb/Cr levels to the same extent as observed in STZ-Vehicle rats after 4 and 7 weeks of treatment, DM-MSC-WJ inhibited albumin secretion in the urine and progression of renal injury similar to Control-MSC (Fig. 8c). Systolic blood pressure tended to decrease in the DM-MSC-WJ-treated group compared with the Vehicle- and DM-MSC-treated groups in the early stage of treatment (Supplementary Fig. S8). Histological findings of the kidney in STZ rats were observed. Abnormal dilatation of the renal tubules and marked accumulation of inflammatory cells by H&E staining, degeneration and atrophic change of tubular epithelium with PAS staining, and fibrotic changes in the interstitial area with Azan staining were observed in STZ-Vehicle rats and STZ-DM-MSC rats (Fig. 8d). These histological damages were improved by administration of either Control-MSC or DM-MSC-WJ (Fig. 8d). The quantitative value from the histological scoring system is shown in Fig. 8e. Ultrastructural abnormalities were observed by TEM in the glomerulus in STZ rats. An expansion of glomerular mesangial matrix and mild swelling of podocyte foot processes were observed in STZ-Vehicle rats (Fig. 8f). Administration of DM-MSC did not suppress the expansion of glomerular mesangial matrix, whereas DM-MSC-WJ did suppress this expansion similar to Control-MSC (Fig. 8f). There was no obvious change in the podocyte foot processes between STZ-Control-MSC rats, STZ-DM-MSC rats, and STZ-DM-MSC-WJ rats.

We also examined the therapeutic effects of BM-MSCs using OLETF rats, a type 2 diabetes model. The experimental protocol and results were shown in Supplementary Fig. S9. LETO-MSC was used as Control-MSC, while OLETF-MSC was used as DM-MSC. Although DM-MSC administration exacerbated U-alb/Cr levels to the same extent as observed in OLETF-vehicle rats after 3 and 6 weeks of treatment (Supplementary Fig. S9c), DM-MSC-WJ inhibited albumin secretion into the urine and progression of renal injury, such as tubular dilatation, atrophy of tubular epithelial cells, inflammatory cell infiltration and fibrosis in interstitial area, similar to Control-MSC in OLETF rats (Supplementary Fig. S9d).

DM-MSC was distributed in the kidney later at day 5 after MSCs injection and quickly disappeared in a few days, whereas DM-MSC-WJ was already distributed in the kidney at day 2 and remained 15 days after MSCs injection (Supplementary Fig. S10a). Distribution of DM-MSC-WJ in the lung tended to increase compared with DM-MSC (Supplementary Fig. S10b), whereas there was no significant difference in the localization in the liver and spleen (Supplementary Fig. S10c,d).

## Discussion

Since DM-MSC lost therapeutic efficacy in DN, we have created a novel activation method for DM-MSC to significantly increase the therapeutic effect of the autologous transplantation. We demonstrated that UC extracts, namely WJs, are a valuable tool for improvement of diabetes-induced abnormalities in BM-MSC. To the best of our knowledge, this is the first study to show the great potency of WJs to improve BM-MSC which has numerous impairments induced by diabetes. BM-MSC activated by WJs had restored proliferative capacity, motility and endoplasmic reticulum functions to produce renal trophic factors, which resulted in amelioration of histological damages in DN. We found that exosomes contained in WJs might be a promising component for this activation.

BM-MSC derived from diabetic models (DM-MSC) including STZ and OLETF rats showed both morphological and functional abnormalities. Cultured DM-MSC showed strong heterogeneity with increased actin filaments in the cytoplasm, flattened, expanded two-dimensional area, and shortened cell projections. We found that the minor axis of the cells passing through the nucleus may represent these morphological abnormalities of the cells, that is, the minor axis was longer in DM-MSC which correlated with enlarged cell area, whereas Control-MSC and DM-MSC-WJ showed spindle-shaped with shorter length of minor axis. Therefore, the length of the minor axis of the cells seems to be useful for the quantitative comparison between normal and abnormal BM-MSC because this index correlates with impairments of organelles, such as ER and mitochondria, and the excessive amount of stress fiber actin. Previous reports have used the total cell area to evaluate proliferation and differentiation potential of aged BM-MSC^29^, whereas no reports have applied the minor axis of cells as an indicator for the abnormalities of BM-MSC.

The proliferative activity was significantly decreased, and mRNA expressions of growth factor and cytokines/chemokines were altered in DM-MSC, which were correlated with enhancement of the ER stress marker XBP-1. Previous studies have shown that hyperglycemia caused BM-MSC abnormality via overproduction of advanced glycation end product (AGE), which induced low proliferation, senescence, apoptosis and abnormal differentiation *in vitro* ^22, 23, 30^. ER stress is a major contributor to chronic metabolic and inflammatory diseases such as type 2 diabetes, obesity and insulin resistance^31, 32^. It also induces the unfolded protein response, which initiates inflammatory responses via the nuclear factor kappa B (NF-κB) pathway^31^. Excessive aggregation of damaged or unfolded proteins induces proteotoxic stress and failure to produce essential proteins to maintain cell function^33^. In contrast, even in normal BM-MSC, cell abnormalities, such as telomere shortening, senescence and elongation of proliferation time occur due to cultured stress caused by sequential cell passaging^34^. In this study, we clarified the variety of abnormalities of DM-MSC, such as severity of ER stress, marked mitochondrial degeneration, down-regulation of growth factor and up-regulation of cytotoxic factors, by comparing STZ-MSC with normal BM-MSC, and OLETF-MSC with age-matched LETO-MSC. These results suggested that DM-MSC has more serious and specific functional abnormalities in addition to those merely induced by culture stress and/or individual aging, which may lead to decreased energetic metabolism and down-regulation of renal trophic factor synthesis by BM-MSC and thus loss of therapeutic effects in DN *in vivo*.

To improve the abnormalities in DM-MSC, we developed UC extracts, namely WJs, as an activator for BM-MSC. UC consists of the amniotic membrane, UC vessels, Wharton’s jelly-derived MSCs and stromal components including Wharton’s jelly, which are a valuable source of biologically active substances^35^. Wharton’s jelly-derived MSCs itself were not focused in this study because the treatment with these cells became allo-transplantations. We showed that WJs contained IGF-1, EGF, PDGF-AB and b-FGF as components of growth factors, hyaluronic acid, collagen and MUC-1 as components of extracellular matrix (ECM), L-Glutamate and rich exosomes. Our results demonstrated that hyaluronic acid and L-Glutamate had no effects, but exosomes purified from WJs (WJ-exosome) improved the abnormalities of DM-MSC almost equal to complete WJs through enhancement of proliferation and suppression of ER stress. Exosomes are 40 -100 nanometer-sized microvesicles which are released from various types of cells, including MSCs, stromal cells, parenchymal cells and cancer cells, under physiological and pathological conditions^36^. In the present study, WJ-exosome is thought to originate from MSCs present in the UC, vascular smooth muscle cells or chorionic cytotrophoblasts^37^. MiR-146a-5p, miR-148a and miR-148b which are contained in exosomes derived from UC-MSC have been reported to enhance the proliferation of MSCs via up-regulation of NF-κB activity or activation of Hedgehog signaling^38, 39^. The present results showed that WJ-exosome alone improved not only the proliferative ability but also morphological impairment and ER stress in DM-MSC, similar to complete WJs. This suggests that WJ-exosome might be a package of cell activation factors and key contributor to functional improvement of the impaired DM-MSC. In contrast, the recovery of mitochondrial abnormality and suppressive effect of α-SMA expression obtained by WJ-exosome alone was relatively weak compared to those obtained from complete WJs. Basic-FGF and IGF-1, which were contained in WJs, are known to suppress autophagy, oxidative stress and senescence of BM-MSC^23, 40^. Therefore, these growth factors in WJs might also contribute to the improvement of abnormal DM-MSC.

WJs improved morphological and functional abnormalities of DM-MSC. WJs greatly improved increased actin filaments in the cytoplasm, flattened, expanded two-dimensional area, shortened cell projections found in DM-MSC. Ultrastructuraly, degenerative mitochondria and swollen ER were recovered. Improvement of energy metabolism in mitochondria and protein synthesis in ER were considered to lead enhancement of proliferation, mobilization, production of trophic factors in DM-MSC. As expected, addition of WJs induced up-regulation of expressions of the growth factor *Igf-1* in DM-MSC. Tomasoni *et al*. reported that the repair of damaged renal proximal tubular cells by MSCs was induced by the combined trophic effect of IGF-1 released by BM-MSC and the transfer of the mRNA of the corresponding IGF-1 receptor via exosomes^41^. Interestingly, our results showed that WJs increased the secretion of exosomes by DM-MSC. We have investigated the effectiveness of BM-MSC for renal injury by inhibiting excessive expressions of proinflammatory cytokine, fibrosis in tubular interstitium, the epithelial-to-mesenchymal transition of tubular epithelial cells^15^. The effectiveness of exosomes has reported via the transfer of mRNAs of hepatocyte growth factor and macrophage-stimulating protein which were packed in exosomes^42, 43^. According to these results, the increase in production and secretion of exosomes by activating DM-MSC might be one of the mechanisms to improve DN.

WJs suppressed excessive actin stress fiber in the cytoplasm and the various proinflammatory cytokine expressions in DM-MSC. This might induce earlier distribution of DM-MSC-WJ by promoting migration ability of cells in the kidney and longer distribution *in vivo* including lung, liver and spleen without exclusion as abnormal substances. The activated cells could thus exert a greater therapeutic effect on impaired renal tissue by paracrine effects of renal trophic factors; for example, a larger number of exosomes secreted from DM-MSC-WJ, which located in the kidney and distant organs.

DM-MSC-WJ restored the therapeutic effects on DN caused by both type 1 and type 2 diabetes *in vivo*. Histologically, DM-MSC-WJ ameliorated the renal tubular epithelial damage, interstitial inflammatory cell infiltration and the fibrotic change common to type 1 and type 2 DN despite differences in the pathology of diabetes and the age of each model. These results indicated that WJs could activate BM-MSCs from various diabetic pathological conditions, and could further demonstrate the therapeutic effect.

In conclusion, we developed a novel method to activate abnormal DM-MSC using UC extracts WJs and demonstrated the morphological and functional improvement of DM-MSC *in vitro*. We further demonstrated that activated DM-MSC possess therapeutic effects in DN *in vivo*. This method may be of great benefit for autologous transplantation of BM-MSC not only for DN patients but also for patients with other diabetic complications and other diseases that BM-MSC exhibits abnormalities.

## Methods

### Animal models of diabetes

Eight-week-old male C57BL/6 mice, 8-week-old male Sprague-Dawley (SD) rats, OLETF rats and LETO rats were purchased from Sankyo Lab Service Corp., Inc. (Tokyo, Japan). T1D models were obtained by a single intraperitoneal administration of 150 mg/kg of STZ (Wako Pure Chemical Industries, Ltd., Osaka, Japan) into 8-week-old C57BL/6 mice to generate high-dose-STZ-induced diabetic mice; by intraperitoneal administration of 40 mg/kg of STZ for 5 consecutive days to generate low-dose-STZ-induced diabetic mice; by a single tail vein injection of 55 mg/kg of STZ into 8-week-old SD rats. Control mice and rats for T1D model were treated with buffer via intraperitoneal and intravenous injection, respectively. T2D model was obtained by the natural development of diabetes in OLETF rats. Control rats for T2D model were obtained by the natural course in LETO rats. Blood glucose levels were monitored using a glucometer (Nipro Carefast Meter; NIPRO Corporation, Osaka, Japan). Successful establishment of the diabetes model was confirmed by blood glucose levels of 400 mg/dl or more for STZ-diabetic mice; by blood glucose levels of 300 mg/dl or more for STZ-diabetic rats; by blood glucose levels of 140 mg/dl or more after fasting for 12 hours for OLETF rats. All methods for the animal experiments were performed in accordance with the relevant guideline and regulations of the animal experiment committee of Sapporo Medical University (Sapporo, Japan). All experimental protocols and studies were approved by the animal experiment committee of Sapporo Medical University (Sapporo, Japan).

### Isolation and culture of rat MSCs

Bone marrow was collected from each model of diabetic rats and control rats. BM-MSC was harvested by adherent cultures of bone marrow cells as described previously^44^.

### Phase contrast microscopic observation of BM-MSC

Morphological findings of BM-MSC were monitored by phase contrast microscopy (Eclipse TE200; Nikon, Tokyo, Japan). Morphological changes of BM-MSC were quantified by measuring the length of the minor axis of the cell passing through the nucleus at the portion perpendicular to the long axis of the nucleus and calculating the average value. Image J software^45^ was used to measure the length of minor axis of cells. All of the cells present in five panels randomly photographed for BM-MSC isolated from individual rats or each condition were analyzed.

### Transmission electron microscopic observation of BM-MSC, glomeruli and exosomes

BM-MSC, renal tissues and exosomes isolated from WJs were observed with a transmission electron microscope (TEM) (H7650; Hitachi High-Technologies Corporation, Tokyo, Japan) as described in Supplementary Methods in detail.

### Proliferation assays of BM-MSC

Proliferation of BM-MSC was analyzed using Cell Counting Kit-8 (CCK-8; Dojindo Laboratories, Kumamoto, Japan) and Ki-67 labeling index, which was determined by the ratio of the number of Ki-67-positive nuclei with respect to the number of total nuclei, as described in Supplementary Methods in detail.

### Quantitative real-time polymerase chain reaction (RT-PCR) of BM-MSC

Quantitative RT-PCR analysis was performed as described in Supplementary Methods in detail using specific primers for rat *Igf-1*, *Ifn-γ*, *Il-1β*, *Il-2*, *Rantes* and *α-Sma*, which are described in Supplementary Table S3. Relative amounts of mRNA were normalized to an internal control, *Gapdh*.

### Immunoblotting of BM-MSC

Protein expression of BM-MSC was analyzed by immunoblotting as described in Supplementary Methods in detail. Cell lysates of cultured BM-MSC were subjected to SDS-PAGE. Anti-XBP-1, anti-α-SMA, anti-BiP, anti-JNK1/3, anti-XBP-1, anti-HRD-1, anti-PERK, anti-phospho-IRE-1α, anti-eIF2α and anti-β-actin antibodies were used to analyze protein expression. Primary and secondary antibodies used for immunoblotting are listed in Supplementary Tables S1 and S2. Expression levels of XBP-1 were semi-quantified using Image J software^45^.

### Intravenous administration of BM-MSC

At 4 weeks after STZ injection, mice were administered 4 times with 1 × 10^4^ Control-MSC (STZ-Control-MSC) or DM-MSC obtained from the bone marrow of STZ rats (STZ-DM-MSC)/g body weight via the tail vein every 2 weeks, whereas controls received vehicle (STZ-Vehicle). Similarly, in SD rat at 4 weeks after STZ injection or in OLETF rat at 6.5 months of age, rats were administered 1 × 10^4^ Control-MSC (STZ-Control-MSC or OLETF-Control-MSC), DM-MSC (STZ-DM-MSC or OLETF-DM-MSC) or DM-MSC activated by WJs (STZ-DM-MSC-WJ or OLETF-DM-MSC-WJ)/g body weight via the tail vein respectively, whereas controls received vehicle (STZ-Vehicle or OLETF-Vehicle).

### Biochemical tests for albuminuria

The mice and rats were housed in metabolic cages and urine was collected for 3 hours. Analysis of albumin and creatinine levels in the urine was performed by SRL, Inc. (Tokyo, Japan). Albumin levels were measured by an immune-turbidimetric method, and creatinine levels were measured by an enzymatic method. Urinary albumin excretion was normalized to urinary creatinine excretion.

### Histological evaluation of renal tissues with quantitative evaluation

Renal tissues obtained from mice and rats was evaluated as described in Supplementary Methods in detail. The histological damage in renal tissues were evaluated quantitatively focusing on tubular epithelium and interstitial changes according to previous reports^18, 46^. Briefly, impairment of tubular epithelium was graded from 0 to 4 according to the presence of tubular dilatation, protein cylinders, and atrophic changes (0, no change; 1, 25% change or less; 2, 25–50% change; 3, 50–75% change; 4, more than 75% change). Interstitial changes were graded from 0 to 4 as described above according to the presence of fibrosis and inflammation. Quantitative analysis was performed in five representative panels per animal at ×100 magnification.

### Histological evaluation of UC tissues

UC tissues were obtained after the caesarean section surgery of full-term babies with the approval of the ethical committee of Sapporo Medical University. Informed consent forms were signed by all donors. Histological findings of UC tissues were evaluated as described in Supplementary Methods in detail.

### Preparation of Wharton’s jelly extract supernatant (WJs) from UC tissues

UC were washed with saline immediately after being obtained to remove blood components. After removing the UC arteries and veins, the sheath of the amnion was cut into 5-mm-wide longitudinal sections. All of the sectioned sheathes of amnion, UC vessels and Wharton’s jelly were collected and suspended serum-free medium and shaken for 72 hours at 4 °C. The supernatants of the tissue suspensions, what we call WJs, were collected and quantified the protein concentrations with bicinchoninic acid (BCA) Protein Assay Kit (Thermo Fisher Scientific). The viscosity of WJs was measured by the tuning fork vibration type viscometer (SV-1A; A&D, Tokyo, Japan) according to the manufacturer’s instructions. WJs were incubated at 37 °C in 5% CO_2_ with 10% of FBS for 96 hours to confirm that the remaining cellular components have lost their viability.

### Identification and quantification of the number of exosomes

Extracellular vesicles ‘exosomes’ were isolated from WJs using exosome precipitation solution (Total Exosome Isolation reagent, Thermo Fisher Scientific) according to the manufacturer’s instructions. Ultrastructures of exosomes were observed by TEM as described previously. Exosomes isolated from WJs were lysed using lysis buffer and the expressions of CD9 and HSP70 were analyzed by immunoblotting. To isolate exosomes secreted from BM-MSC, cells was cultured with or without WJs for 48 hours, subsequently the medium was changed into fresh without WJs, and the supernatant was collected 24 hours after changing the medium. Exosomes were isolated from the supernatant using Total Exosome Isolation reagent (Thermo Fisher Scientific). The number of exosomes isolated from WJs and supernatants of BM-MSC were quantified using CD9 Exo enzyme linked immunosorbent assay (ELISA) (System Biosciences, Inc., Palo Alto, CA).

### Measurement of the physiologically active substance in WJs

Growth factors such as IGF-1, EGF, PDGF-AB and b-FGF were evaluated using Quantikine ELISA kit for human (R&D Systems, Inc., Minneapolis, MN). Concentration of L-Glutamate in WJs was measured using F-kit L-Glutamate (J.K. International Inc., Tokyo, Japan) by calculating molecular absorption factors. Concentration of hyaluronic acid in WJs was measured using Quantikine ELISA kit (R&D Systems, Inc.). WJs samples were diluted with α-MEM 50-fold for the measurement of growth factors and L-Glutamate, and 5000-fold for the measurement of hyaluronic acid for optimum analysis.

### Activation of DM-MSC with WJs

STZ-MSC and OLETF-MSC at passage 3 were activated by adding WJs into the culture medium at various concentrations and culturing for 12 to 96 hours. Activated DM-MSC were used for various analyses or harvested for treatment of STZ rats *in vivo*.

### Scratch assay

The migration ability of BM-MSC was evaluated by measuring the area of open wound 12 hours after scratching the cell monolayer as described in Supplementary Methods in detail. After acquiring the images, five points of open area at 0 and 12 hours were quantified using Image J software^45^.

### Statistical analysis

Data from quantitative experiments were expressed as mean ± standard error (SE) values. Analysis of variance was employed for multiple comparisons. Two-way repeated measures (mixed between-within subjects) analysis of variance followed by Bonferroni’s test was used for serial assessment. Statistical analysis was performed using GraphPad Prism 6.0 (GraphPad Software Inc., San Diego, CA). Differences were considered significant at *P* < 0.05 for all two-tailed tests.

### Ethics

Animal experimentation: All experimental protocols and studies were approved by the animal experiment committee of Sapporo Medical University (Sapporo, Japan).

Human study: The human study was conducted in accordance with the ethical principles of the Declaration of Helsinki and was approved by the ethical committee of Sapporo Medical University (Registration numbers; 24-142, 25-1227, 262-1031, 262-1046, 262-110). Written informed consent was received from participants prior to inclusion in the study.

## Electronic supplementary material

Supplementary information
