# Supplementary material for: Umbilical cord extracts improve diabetic abnormalities in bone marrow-derived mesenchymal stem cells and increase their therapeutic effects on diabetic nephropathy
Source: Sci Rep. 2017 Aug 16;7:8484. doi: 10.1038/s41598-017-08921-y (PMC5559488; doi:10.1038/s41598-017-08921-y)
Supplement: Supplementary file 1 — Supplementary information [file 41598_2017_8921_MOESM1_ESM.pdf]

SUPPLEMENTARY INFORMATION

1. Supplementary Results

2. Supplementary Methods

3. Supplementary Discussion

4. Supplementary References

5. Supplementary Table and Figure Legends

6. Supplementary Tables

7. Supplementary Figures

Title:

Umbilical cord extracts improve diabetic abnormalities in bone marrow-derived mesenchymal stem cells and increase their therapeutic effects on diabetic nephropathy

Kanna Nagaishi*, Yuka Mizue, Takako Chikenji, Miho Otani, Masako Nakano, Yusaku Saijo, Hikaru Tsuchida, Shinichi Ishioka, Akira Nishikawa, Tsuyoshi Saito, Mineko Fujimiya

Supplementary Results

Morphology and proliferative ability of bone marrow-derived mesenchymal stem cells (BM-MSC) depending on the duration of diabetes

Morphological findings of BM-MSC derived from streptozotocin (STZ)-induced diabetic rats (STZ-MSC) and Otsuka Long-Evans Tokushima Fatty (OLETF) diabetic rats (OLETF-MSC) were abnormal with short cell processes and flattened enlarged individual cell area with increased actin fibers in the cytoplasm compared with Control-MSC and Long-Evans Tokushima Otsuka (LETO)-MSC, respectively. STZ-MSC isolated from rats at 12 weeks after administration of STZ showed more severe abnormalities than that isolated at 4 and 8 weeks (Supplementary Fig. S1a). A similar trend was observed in OLETF-MSC. OLETF-MSC isolated from rats at 8-month-old showed more severe abnormalities than that isolated at 4.5-month-old (Supplementary Fig. S2a). Proliferation of DM-MSC was significantly reduced in STZ-MSC and OLETF-MSC compared with Control-MSC and LETO-MSC, respectively. Cell growth of the BM-MSC was reduced in accordance with the duration of diabetes in STZ rats and OLETF rats (Supplementary Figs S1b and S2b).

Immunophenotype and differentiation potentials into multiple mesenchymal lineages of BM-MSC

STZ-MSC and OLETF-MSC expressed cluster of differentiation (CD) 90 and CD44 cell surface antigens, as did Control-MSC and LETO-MSC, while CD45, CD34,

CD31, CD11b and HLA-DR were not expressed. This expression pattern is characteristics of rat MSCs, as described previously (Supplementary Fig. S1c,d and S2c,d). STZ-MSC and OLETF-MSC exhibited similar adipogenic differentiation potentials to Control-MSC and LETO-MSC, respectively (Supplementary Fig. S1e, S2e). The osteogenic differentiation potentials of STZ-MSC and OLETF-MSC were lower than those of Control-MSC and LETO-MSC, respectively (Supplementary Fig. S1f, S2f).

Blood glucose levels and urinary albumin-creatinine ratio in low dose STZ-administered diabetic mice

We compare blood glucose levels and urinary albumin-creatinine ratio (U-alb/Cr) in mice administered a single high dose (150 mg/kg) of STZ and mice administered five low doses (40 mg/kg) of STZ (Supplementary Fig. S3a,b). Because there was no difference in U-alb/Cr between the two groups, we used the single high-dose administration model in this study.

Insulin expressions in pancreatic islet tissues of STZ-induced diabetic mice

Insulin expression was markedly reduced in STZ mice, and was not increased by vehicle and Control-MSC treatments. These results corresponded to a lack of improvement in blood glucose levels in STZ mice treated with the various BM-MSC (Supplementary Fig. S3c).

Therapeutic effect of insulin pellet on STZ-induced diabetic mice

The experimental protocol for glycaemic control with insulin pellets in STZ mice is shown in Supplementary Fig. S4a. To distinguish the drug toxicity of STZ and influence of hyperglycaemia on the histological findings of the kidney, STZ mice were treated with insulin pellets. Blood glucose levels began to decline from the day after pellet transplantation and were maintained at 200 mg/dL or less continuously after 1 week of treatment (Supplementary Fig. S4b). Serum glycoalbumin levels were significantly reduced in the insulin-treated group compared with the untreated group at 4 weeks after transplantation (Supplementary Fig. S4c). Histological findings for the kidneys of STZ mice demonstrated abnormal dilatation of the renal tubules and mild inflammatory cell infiltration in mice without insulin treatment (Supplementary Fig. S4e, middle panels). Although abnormal dilatation of the renal tubules was clearly improved, accumulation of inflammatory cells was unchanged by insulin treatment (Supplementary Fig. S4e, right panels). There was no significant difference in U-alb/Cr levels in mice with or without glycaemic control for 4 weeks (Supplementary Fig. S4d).

Morphological improvement of DM-MSC by addition of Wharton’s jelly extract supernatant

Under the phase contrast microscope, addition of Wharton’s jelly extract supernatant (WJs) changed the morphology of STZ-MSC and OLETF-MSC into thicker

and spindle-shaped cells with thinner and longer processes. These effects were enhanced in a concentration-dependent manner of WJs (Supplementary Figs S5a and S6a).

Mechanism of WJs reducing ER stress in DM-MSC

STZ-MSC and OLETF-MSC were cultured with 4-phenylbutyrate (PBA) or WJs to compare the suppressive effect of ER stress. The expression of HMG-CoA Reductase Degradation 1 (HRD-1), PKR-like ER kinase (PERK) and phospho- Inositol Requiring (IRE) -1α and translation initiation factor (eIF) 2α were up-regulated by the stimulation with PBA as similar as addition of WJs in STZ-MSC and OLETF-MSC (Supplementary Figs S5b and S6b). On the other hand, Control-MSC were stimulated with Tunicamycin (TM), Thapsigargin (TG) and high-glucose (HG) culture media, respectively. Any of TM, TG and HG increased the expression of XBP-1. High dose of TG and HG suppressed PERK, while TM and low dose of TG up-regulated PERK conversely (Supplementary Fig. S5c). There was no obvious change in ERAD and IRE-1α pathway.

Exposure time and concentration dependency of WJs on the suppressive effect of actin stress fibers in STZ-MSC and OLETF-MSC

The excessive expression of α-SMA in the cytoplasm of STZ-MSC and OLETF-MSC was decreased by culturing with WJs. The suppressive effect was

enhanced in time- and concentration-dependent manner in STZ-MSC-WJ (Supplementary Fig. 5d,e) and OLETF-MSC-WJ (Supplementary Fig. S6c,d).

Immunophenotype and differentiation potentials into multiple mesenchymal lineages of DM-MSC activated by WJs

Addition of WJs did not affect the expression of cell surface antigens in STZ-MSC or OLETF-MSC (Supplementary Figs S5f and S6e). Addition of WJs increased the adipogenic differentiation potentials of STZ-MSC and OLETF-MSC (Supplementary Figs S5g and S6f). Conversely, addition of WJs reduced the osteogenic differentiation potentials of STZ-MSC and OLETF-MSC (Supplementary Figs S5h and S6g).

Insulin expressions in pancreatic islet tissues of STZ-induced diabetic rats

Insulin expression was markedly reduced in STZ rats, and was not increased by any of vehicle, DM-MSC and DM-MSC-WJ treatments. These results corresponded to a lack of improvement in blood glucose levels in STZ rats treated with various BM-MSC (Supplementary Fig. S7).

Change in systolic blood pressure in STZ rats

Systolic blood pressure tended to decrease in the DM-MSC-WJ-treated group compared with the vehicle- and DM-MSC-treated groups in the early stage of treatment (Supplementary Fig. S8).

OLETF-MSC activated by WJs ameliorated renal injury in OLETF rats

We also examined the therapeutic effects of BM-MSCs using OLETF rats, a type 2 diabetes model. The experimental protocol for BM-MSC therapies in OLETF rats is shown in Supplementary Fig. S9a. LETO-MSC was used as Control-MSC, while OLETF-MSC was used as DM-MSC. Blood glucose levels did not change in any of groups (Supplementary Fig. S9b). We initiated the treatment when albuminuria was well developed at an age of 6.5 months. Although DM-MSC administration exacerbated U-alb/Cr levels to the same extent as observed in OLETF-vehicle rats after 3 and 6 weeks of treatment (Supplementary Fig. S9c), DM-MSC-WJ inhibited both albumin secretion into the urine and progression of renal injury, such as tubular dilatation, atrophy of tubular epithelial cells, inflammatory cell infiltration and fibrosis in interstitial area, similar to Control-MSC in OLETF rats (Supplementary Fig. S9d). The glomerular abnormality was mild even in OLETF-vehicle rats.

Distribution of STZ-MSC and STZ-MSC activated by WJs

Abnormal STZ-MSC were detected in the kidney at day 5 after MSC injection and quickly disappeared after a few more days. Conversely, activated STZ-MSC (STZ-

MSC-WJ) were detected in the kidney earlier, at day 2 after injection, and still detectable 15 days after injection (Supplementary Fig. S10a). Larger numbers of STZ-MSC-WJ were detected also in the interstitium of the lung, liver and spleen, compared with abnormal DM-MSC (Supplementary Fig. S10b–d).

Supplementary Discussion

To investigate the mechanism of WJs reducing ER stress in DM-MSC, STZ-MSC and OLETF-MSC were cultured with 4-phenylbutyrate (PBA)[1], which inhibited ER stress by promoting chemical chaperone activity, or WJs. The expression of HRD-1[2], which is ER-associated degradation (ERAD)-associated E3 ubiquitin-protein ligase, PERK[3], eIF2α and phospho-IRE-1α[4], which are ER transmembrane proteins and its downstream signaling molecule, were up-regulated by the stimulation with PBA and the addition of WJs in STZ-MSC and OLETF-MSC. Because WJs suppressed the ER stress as similar as PBA, it was thought that inhibition of ER stress is one of mechanism of WJs. On the other hand, to investigate whether HG induces ER stress in normal MSC, Control-MSC were stimulated with Tunicamycin (TM), which is an ER stress inducer by inhibiting N-glycosylation of protein[4], or Thapsigargin (TG), which is an inhibitor of endoplasmic reticulum Ca^{2+}-ATPase and decrease in ER calcium levels[3], or HG culture media . The expression of XBP-1 was increased by the stimulation with TM, TG and HG in Control-MSC. Because HG suppressed the expression of PERK, it was thought that hyperglycemia mainly induces ER stress via PERK pathway.

The osteogenic differentiation potentials of BM-MSC in both STZ rats and OLETF rats were significantly decreased. Previous studies have shown that hyperglycemia down-regulated the osteogenic differentiation of BM-MSC via the suppression of heme oxygenase-1[5,6], down-regulation of bone morphogenetic protein 2[7], or induction of apoptosis and senescence via an increase of the receptor for AGEs[8]. Several studies reported the relationship between diabetes and osteoporosis based on MSC abnormality and endocrine roles for both bone and adipose tissue[9,10]. The adipogenic differentiation potentials were similar in STZ-MSC and Control-MSC and in OLETF-MSC and LETO-MSC, respectively. These results suggested that hyperglycemia affected seriously the ostegenic differentiation potentials of DM-MSC. On the other hand, WJs up-regulated adipogenic differentiation potentials and down-regulated osteogenic differentiation potentials in both STZ-MSC and OLETF-MSC. These results suggested that the differentiation potentials of DM-MSC did not affect the therapeutic effects on DN directly.

STZ is commonly used to induce diabetes in experimental animal models and frequently to investigate the pathogenesis of human diabetic nephropathy. However, the cytotoxicity of STZ reportedly affects the pathology of nephropathy in STZ-induced diabetic models [11,12]. Tay YC and Tesch GH reported that focal area of acute tubular necrosis (ATN) with tubular vacuolisation is the primary tissue disorders occurring in the early phase (at 1, 2 and 6 weeks after the administration of STZ) of high-dose (> 200 mg/kg) STZ administered diabetic nephropathy (DN) in model mice. Tay YC also suggested the optimal dose of STZ to induce DN in mice to include two lower doses of STZ, such as 75 mg/kg at the first time and 150 mg/kg at the second time or 150 mg/kg

at the first time and 150 mg/kg at the second time intravenously injected in 5 days apart. We administered 150 mg/kg of STZ only once, which induced lower irritation than occurred with the above protocol. Although mild interstitial hypercellularity and tubular dilatation were observed, ATN and/or interstitial fibrosis were not observed histologically in STZ mice during the early phase (5 weeks after the injection of STZ). Furthermore, to distinguish the drug toxicity of STZ and the influence of hyperglycemia in the histological findings of the kidney, we regulated blood glucose levels by the transplantation of insulin pellets in STZ mice. Although excessive tubular dilatation improved with glycaemic control, interstitial hypercellularity and U-alb / Cr levels were not improved by its treatment alone. These results suggested that glycaemic control alone was insufficient for cellular infiltration and subsequent fibrotic changes. It was also reflected in the result that U-alb / Cr did not improve.

In addition, we also examined the therapeutic effects of BM-MSCs using OLETF rats to avoid the effects of STZ toxicity on nephropathy. As DM-MSC-WJ inhibited albumin secretion into the urine in OLETF rats similar to STZ rats. Histologically, DM-MSC-WJ ameliorated the renal tubular epithelial damage, interstitial inflammatory cell infiltration and the fibrotic change common to type 1 and type 2 DN despite differences in the pathology of diabetes and the age of each model. These results indicated that WJs could activate BM-MSCs from various diabetic pathological conditions, and could further demonstrate the therapeutic effect.

DM-MSC was detected in the kidney later and disappeared earlier than activated DM-MSC (DM-MSC-WJ). The reason for the later distribution was due to a delay in redistribution from the lung caused by low mobility of DM-MSC. The reason

for the earlier disappearance of DM-MSC from the kidney was due to their identification as an abnormal substance and consequent removal. These abnormalities of DM-MSC were ameliorated by activation with WJs in DM-MSC-WJ. WJs improved the excessive actin stress fiber in the cytoplasm and mobility of DM-MSC. Furthermore, WJs down-regulated various proinflammatory cytokine expressions, such as *Ifn-γ, Il-1β, Il-2 and Rantes*, in DM-MSC. These effects might induce the effective distribution and enhance therapeutic effects of DM-MSC-WJ by reducing the adverse immune reactions and avoid early exclusion of activated DM-MSC *in vivo*.

Supplementary Methods

Isolation, culture, and characterization of rat BM-MSC

Bone marrow was collected from each model of diabetic rats and control rats. BM-MSC was harvested by adherent cultures of bone marrow cells as described previously[13]. Briefly, bone marrow cells were harvested from femurs and tibias by flushing whole bone marrow with complete α-modified Eagle's medium (α-MEM; Invitrogen) containing 15% fetal bovine serum and 1% penicillin-streptomycin. Single cell suspensions were filtered through a 70-μm nylon filter (Becton Dickinson) and plated in 75-cm^2 flasks. The cells were grown in complete α-MEM at 37°C and 5% CO_2. After 72 hours, the medium was replaced with fresh medium, and adherent cells grown to 80% confluency to obtain samples were defined as passage 0. Cells in passage 3 were used for experiments. Characterization of rat MSCs was determined by fluorescence-activated cell sorting (Calibur; BD Bioscience) using rat surface antigen–

specific antibodies, CD90, CD44, CD45, CD43, CD31, CD11b and HLA-DR (Immunotech-Beckman Coulter). The *in vitro* differentiation potential of BM-MSC was confirmed by previously described methods[14]. Briefly, rat MSCs were cultured with adipogenic and osteogenic differentiation medium (Takara Bio Inc.) for 10 to 14 days, following the manufacturer's instructions. Adipogenic differentiation was detected by Oil red O staining (Sigma-Aldrich). Osteogenic differentiation was detected using an Alkaline Phosphatase Staining Kit (Primary Cell Co., Ltd.).

Transmission electron microscopic observation of BM-MSC, glomeruli and exosomes

BM-MSC, renal tissues and exosomes isolated from WJs were fixed for 24 hours with 2.5% glutaraldehyde (Wako Pure Chemical Industries, Ltd.). Samples were washed with PBS, fixed with 1% osmium tetroxide solution (TAAB Laboratories Equipment Ltd., Aldermaston, England) and dehydrated with ethanol (Wako Pure Chemical Industries, Ltd.). After soaking the sample in propylene oxide (KANTO KAGAKU, Tokyo, Japan), samples were embedded with an epoxy resin (TAAB Laboratories Equipment Ltd.) and polymerized with heating. Ultrathin sections (70-nm-thickness) of the sample were prepared using the ultramicrotome (MT-X; RMC Boeckeler Instruments, Inc., Tucson, AZ). Samples were observed with a transmission electron microscope (TEM) (H7650; Hitachi High-Technologies Corporation, Tokyo, Japan) after electron staining was performed.

Proliferation assays of BM-MSC

Three thousand BM-MSCs were prepared in 96-well cell culture plates (Corning Costar; Sigma-Aldrich, St. Louis, MO) and cultured with or without WJs for 24 - 72 hours. Triplicate wells were used for each sample. Proliferation of BM-MSC was analyzed using Cell Counting Kit-8 (CCK-8; Dojindo Laboratories, Kumamoto, Japan). Briefly, cells were treated with 10 μL of tetrazolium salt (WST-8) in each well for another 3 hours. The absorbance at 490 nm (A450) was measured with a microplate reader (Infinite M1000 Pro; TECAN, Männedorf, Switzerland).

Thirty thousand BM-MSCs were prepared in Lab-Tek II Chamber Slides (2-well type, Thermo Fisher Scientific, Waltham, MA) and cultured with or without WJs for 48 hours. After cells were immersed with 4% paraformaldehyde, Ki-67 immunohistochemistry was performed using primary antibodies and secondary antibodies (Supplementary Tables S1 and S2). Nuclei were stained with DAPI (Dojindo Laboratories) and observed by confocal laser scanning microscopy (LSM 510; Carl Zeiss, Oberkochen, Germany) using 420 and 590 nm filters. Ki-67 labeling index was determined by the ratio of the number of Ki-67-positive nuclei with respect to the number of total nuclei. All cells present in five panels randomly photographed for BM-MSC isolated from individual rats or each conditions of cells were analyzed.

Quantitative real-time polymerase chain reaction (RT-PCR) analysis

Total RNA was extracted using TRIzol® Plus RNA Purification Kit (Invitrogen), and 500 ng total RNA was reverse-transcribed into cDNA with oligo-dT

primers using the Omniscript RT kit (Qiagen). Quantitative RT-PCR was performed using an ABI PRISM® 7000 Sequence Detection System SDS (Applied Biosystems) with SYBR® Green 1 PCR Master Mix (Applied Biosystems) for 40 cycles of a two-step amplification (95°C for 15 seconds and 60°C for 1 minutes). Melting curves using Dissociation Curves software (Applied Biosystems) ensured that only a single product was amplified. Data were analyzed using comparative Ct Method (ΔΔCT Method). Specific primers used for rat *Igf-1*, *Ifn-γ*, *Il-1β*, *Il-2*, *Rantes* and *α-Sma* are shown in Supplementary Table S3. The rat *Gapdh* primer acted as an internal standard for RNA integrity and quantity.

Immunoblotting

Protein expression was analyzed by immunoblotting. BM-MSC and exosomes isolated from WJs were lysed in Radio Immuno Precipitation Assay buffer that included 20 mM Tris-HCl, pH 7.4, 150 mM sodium chloride, 1 mM EDTA (Sigma-Aldrich), pH 8.0, 0.1% (w/v) sodium dodecyl sulfate, 0.1% sodium deoxycholate, and 1% TritonX-100, and one tablet of complete Mini™ (Roche Diagnostics) and Phos STOP (Roche Diagnostics). Five to 40 μg of each lysate, as determined protein concentrations by bicinchoninic acid (BCA) Protein Assay Kit (Thermo Fisher Scientific), was resolved on a 12% denaturing polyacrylamide gel and transferred to a polyvinylidene difluoride membrane. After blocking with 5% nonfat dry milk in Tris buffered saline with Tween 20, the membrane was incubated with primary antibodies (Supplementary Table S1). It was then incubated with horseradish peroxidase-conjugated secondary antibodies

(Supplementary Table S2). Immuno-reactivity was developed using the enhanced chemiluminescence kit (Amersham Biosciences).

Regulation of ER stress in BM-MSC

STZ-MSC and OLETF-MSC were cultured with each concentration of 4-phenylbutyrate (PBA, Sigma-Aldrich, St. Louis, MO). In addition, Control-MSC was cultured with each concentration of Tunicamycin (TM, Sigma-Aldrich, St. Louis, MO), Thapsigargin (TG, Wako Pure Chemical Industries, Ltd., Osaka, Japan) and high-glucose culture media for 24 hours, respectively. The expression of HRD-1, PERK, phospho-IRE-1α and translation initiation factor (eIF) 2α were analyzed by immunoblotting. Primary and secondary antibodies were listed in Supplementary Table S1 and S2.

Transplantation of insulin pellets into STZ mice

After confirming that the blood glucose level had risen to above 400 mg/dL 1 week after STZ administration, insulin pellets (LinBit, LinShin Canada, Ontario, Canada) were placed subcutaneously under the middle dorsal skin of STZ mice. Blood glucose levels were monitored weekly. Glycoalbumin levels were analysed by consignment (FUJIFILM Monolith, Tokyo, Japan).

Histological evaluation of renal tissues with quantitative evaluation

Renal tissues obtained from mice and rats were fixed in 4% paraformaldehyde. Paraffin-embedded sections of kidney were stained with H&E (Wako Pure Chemical Industries, Ltd.). Kidney sections were also stained with PAS and Azan (Muto Pure Chemicals, Tokyo, Japan). Stained sections were observed with a light microscope (NIS element BR 3.0; Nikon). The histological damage in the kidney was evaluated quantitatively focusing on tubular epithelium and interstitial changes.

Histological evaluation of UC tissues

UC tissues were obtained after the caesarean section surgery of full-term babies with the approval of the ethical committee of Sapporo Medical University. Informed consent forms were signed by all donors. The middle portion of the UC was fixed in 4% paraformaldehyde. The paraffin-embedded sections of UC were stained with H&E (Wako Pure Chemical Industries, Ltd.), MUC1 antibody and DAB (3,3'-Diaminobenzidine, Dojindo Laboratories) substrate-chromogen. The sections were analyzed with light microscope (NIS element BR 3.0). Frozen section of UC was stained with anti-hyaluronic acid, anti-type 1 collagen, and anti-type 3 collagen antibodies to analyze protein expressions. Primary antibodies and secondary antibodies used for immunofluorescence staining are listed in Supplementary Tables S1 and S2. Nuclei were stained with DAPI (Dojindo Laboratories) and observed by confocal laser scanning microscopy (LSM 510) using 420 and 590 nm filters.

Histological evaluation of pancreatic islet tissues

Pancreatic tissues obtained from mice and rats were fixed in 4% paraformaldehyde. Frozen sections of pancreas tissue were stained with anti-insulin-proinsulin and anti-glucagon antibodies to analyze protein expressions. Primary antibodies and secondary antibodies used for immunofluorescence staining are listed in Supplementary Tables S1 and S2.

Measurement of systolic blood pressure in rats

Systolic blood pressure was measured in STZ rats using non-invasive blood pressure monitor (MK-2000ST, Muromachi Kikai Co., Ltd. Tokyo, Japan). The blood pressure was measured 5 to 8 times in each rat, and the average value was taken as the value of each rat.

Detection of donor BM-MSC

Forty-eight hours after culturing with or without WJs, STZ-MSC and STZ-MSC-WJ were marked with PKH26 Red Fluorescent Cell Linker Kit (Sigma-Aldrich) and administered to STZ induced diabetic rats by tail vein injection. Rats were euthanized at 2, 5, 8 or 15 days after MSCs injection, and kidneys, lungs, liver and spleen were collected. These organs were immersed in 4% paraformaldehyde for 48 hours. Frozen sections of each organ were stained with DAPI (Dojindo Laboratories) at 0.1 mg/mL. The distribution of MSCs expressing red fluorescence in each organ was observed by confocal laser scanning microscopy (LSM 510; Carl Zeiss).

Supplementary References

1. Kim, H. J. *et al.* Inhibition of endoplasmic reticulum stress alleviates lipopolysaccharide-induced lung inflammation through modulation of NF-kappaB/HIF-1alpha signaling pathway. *Sci. Rep.* **3**, 1142, doi:10.1038/srep01142 (2013).
2. Oslowski, C. M. & Urano, F. Measuring ER stress and the unfolded protein response using mammalian tissue culture system. *Methods Enzymol.* **490**, 71-92, doi:10.1016/B978-0-12-385114-7.00004-0 (2011).
3. Hirasawa, H., Jiang, C., Zhang, P., Yang, F. C. & Yokota, H. Mechanical stimulation suppresses phosphorylation of eIF2alpha and PERK-mediated responses to stress to the endoplasmic reticulum. *FEBS Lett.* **584**, 745-752, doi:10.1016/j.febslet.2009.12.028 (2010).
4. Iwawaki, T. *et al.* Translational control by the ER transmembrane kinase/ribonuclease IRE1 under ER stress. *Nat. Cell Biol.* **3**, 158-164, doi:10.1038/35055065 (2001).
5. Barbagallo, I. *et al.* Overexpression of heme oxygenase-1 increases human osteoblast stem cell differentiation. *J. Bone Miner. Metab.* **28**, 276-288, doi:10.1007/s00774-009-0134-y (2010).
6. Vanella, L., Sanford, C., Jr., Kim, D. H., Abraham, N. G. & Ebraheim, N. Oxidative stress and heme oxygenase-1 regulated human mesenchymal stem cells differentiation. *Int. J. Hypertens.* **2012**, 890671, doi:10.1155/2012/890671 (2012).

7 Wang, J. *et al.* High glucose inhibits osteogenic differentiation through the BMP signaling pathway in bone mesenchymal stem cells in mice. *EXCLI J* **12**, 584-597 (2013).

8 Stolzing, A., Sellers, D., Llewelyn, O. & Scutt, A. Diabetes induced changes in rat mesenchymal stem cells. *Cells Tissues Organs* **191**, 453-465, doi:10.1159/000281826 (2010).

9 de Paula, F. J., Horowitz, M. C. & Rosen, C. J. Novel insights into the relationship between diabetes and osteoporosis. *Diabetes Metab. Res. Rev.* **26**, 622-630, doi:10.1002/dmrr.1135 (2010).

10 Tan, J. *et al.* The Influence of Diabetes Mellitus on Proliferation and Osteoblastic Differentiation of MSCs. *Curr. Stem Cell Res. Ther.* (2015).

11 Tay, Y. C. *et al.* Can murine diabetic nephropathy be separated from superimposed acute renal failure? *Kidney Int.* **68**, 391-398, doi:10.1111/j.1523-1755.2005.00405.x (2005).

12 Tesch, G. H. & Allen, T. J. Rodent models of streptozotocin-induced diabetic nephropathy. *Nephrology (Carlton)* **12**, 261-266, doi:10.1111/j.1440-1797.2007.00796.x (2007).

13 Javazon, E. H., Colter, D. C., Schwarz, E. J. & Prockop, D. J. Rat marrow stromal cells are more sensitive to plating density and expand more rapidly from single-cell-derived colonies than human marrow stromal cells. *Stem Cells* **19**, 219-225, doi:10.1634/stemcells.19-3-219 (2001).

14 Romanov, Y. A., Svintsitskaya, V. A. & Smirnov, V. N. Searching for alternative sources of postnatal human mesenchymal stem cells: candidate MSC-

like cells from umbilical cord. *Stem Cells* **21**, 105-110, doi:10.1634/stemcells.21-1-105 (2003).

Supplementary Figure Legends

Supplementary Figure S1. Characterization of bone marrow-derived mesenchymal stem cells (BM-MSC) isolated from control rats (Control-MSC) and streptozotocin (STZ)-induced diabetic rats (STZ-MSC)

(a) Phase contrast images of BM-MSC (passage 3) isolated from 4-week-STZ loaded rats (STZ-4w), 8-week-STZ loaded rats (STZ-8w) and 12-week-STZ loaded rats (STZ-12w). Bar: 100 µm. (b) MTT proliferation assays of STZ-MSC. BM-MSC isolated from 12 to 16-week-STZ loaded rats was compared with BM-MSC isolated from 4 to 8-week-STZ loaded rats. Absorbance at 450 nm was measured at 0, 24 and 48 hours after the addition of MTT. Values are means ± SE of n=3, each. *P < 0.05. (c) (d) Immunophenotype analysis of Control-MSC (c) and STZ-MSC (d) surface antigen expression by flow cytometry. Red lines represent target antibodies and blue lines represent isotype controls. (e)(f) Adipogenic and osteogenic differentiation of Control-MSC and STZ-MSC. Fat droplets are visualized by Oil red O staining (red). Bone matrix is visualized by alkaline phosphatase staining (blue). Bar: 100 µm (e) and 500 µm (f).

Supplementary Figure S2. Characterization of BM-MSC isolated from Long-Evans Tokushima Otsuka (LETO) rats (LETO-MSC) and Otsuka Long-Evans Tokushima Fatty (OLETF) diabetic rats (OLETF-MSC)

(a) Phase contrast images of BM-MSC (passage 3) isolated from 4.5-month-old OLETF rats (OLETF-4.5M) and 8-month-old OLETF rats (OLETF-8M). Bar: 100 µm. (b) MTT proliferation assays of OLETF-MSC compared with LETO-MSC. Absorbance at 450 nm was measured at 0, 24 and 48 hours after the addition of MTT. Values are means ± SE of n=3, each. *$P < 0.05$. (c) (d) Immunophenotype analysis of LETO-MSC (c) and OLETF-MSC (d) surface antigen expression by flow cytometry. Red lines represent target antibodies and blue lines represent isotype controls. (e)(f) Adipogenic and osteogenic differentiation of LETO-MSC and OLETF-MSC. Fat droplets are visualized by Oil red O staining (red). Bone matrix is visualized by alkaline phosphatase staining (blue). Bar: 100 µm (e) and 500 µm (f).

Supplementary Figure S3. Analysis of pancreatic and renal function in STZ-induced diabetic mice

(a) Changes in blood glucose levels in control mice and low dose STZ (40 mg/kg) -administered mice. Values are expressed as mean ± SE of 6 animals in the control group and 26 animals in the STZ group. (b) Changes in urine albumin/creatinine ratio (U-alb/Cr) in control mice and low dose STZ (40 mg/kg) -administered mice. Values are expressed as mean ± SE of 6 animals in the control group and 26 animals in the STZ

group. (c) Immunofluorescence staining of proinsulin (red) in islet tissues of control and STZ-induced diabetic mice. DAPI was used to counterstain nuclei (blue). Bar: 50 µm.

Supplementary Figure S4. Therapeutic effect of insulin pellets for diabetic nephropathy in STZ-induced diabetic mice

(a) Experimental protocol for insulin therapies in streptozotocin (STZ)-induced diabetic mice. (b) Changes in blood glucose levels after transplantation of insulin pellets. Values are expressed as mean ± SE of 3–9 animals. *$P < 0.05$ STZ-Ins+ vs. STZ-Ins-. (c) Glycoalbumin levels in serum at 4 weeks after transplantation of insulin pellets. Values are expressed as mean ± SE of 3–9 animals. *$P < 0.05$. (d) Changes of urine albumin/creatinine ratio (U-alb/Cr) after transplantation of insulin pellets. Values are expressed as mean ± SE of 3–9 animals. *$P < 0.05$ STZ-Ins- vs. Control, **$P < 0.05$ STZ-Ins+ vs. Control. (e) Histological findings for renal cortex using haematoxylin and eosin, Periodic acid-Schiff, and Azan staining of kidney sections at 4 weeks after insulin pellet transplantation. Bar: 100 µm.

Supplementary Figure S5. Activating effects of Wharton's jelly extract supernatant (WJs) for STZ-MSC

(a) Phase contrast images of STZ-MSC cultured with 0 to 2.0 mg/ml WJs. Bar: 100 µm. (b) Western blot analysis of STZ-MSC cultured with 5 mM or 10 mM 4-phenylbutyric acid (PBA) and 1.0 mg/ml WJs, using anti-HRD-1, anti-PERK, anti-phospho-IRE-1a,

anti-eIF2a and anti-β-actin antibodies. The cropped images of immunoblots displayed in the figure and the full-length blots were shown in Supplementary Fig. S19 online. (c) Western blot analysis of control-MSC cultured with 0.1, 0.5 or 1.0 mg/ml tunicamycin (TM); 1 or 10 mM thapsigargin (TG); or 300 or 450 mg/dL high glucose medium (HG), using anti-XBP-1, anti-PERK and anti-β-actin antibodies. The cropped images of immunoblots displayed in the figure and the full-length blots were shown in Supplementary Fig. S20 online. (d) Western blot analysis of STZ-MSC cultured with 0 to 1.0 mg/ml WJs, using anti-α-SMA and anti-β-actin antibodies. The cropped images of immunoblots displayed in the figure and the full-length blots were shown in Supplementary Fig. S21 online. (e) Western blot analysis of STZ-MSC cultured with or without WJs owing to the difference of an additional period from 12 to 96 hours, using anti-α-SMA and anti-β-actin antibodies. The cropped images of immunoblots displayed in the figure and the full-length blots were shown in Supplementary Fig. S22 online. (f) Immunophenotype analysis of STZ-MSC-WJ surface antigen expression by flow cytometry. Red lines represent target antibodies and blue lines represent isotype controls. (g)(h) Adipogenic and osteogenic differentiation of STZ-MSC and STZ-MSC-WJ. Fat droplets are visualized by Oil red O staining (red). Bone matrix is visualized by alkaline phosphatase staining (blue). Bar: 100 μm (g) and 500 μm (h).

Supplementary Figure S6. Activating effects of WJs for OLETF-MSC

(a) Phase contrast images of OLETF-MSC cultured with 0 to 2.0 mg/ml WJs. Bar: 100 μm. (b) Western blot analysis of OLETF-MSC cultured with 5 mM or 10 mM 4-

phenylbutyric acid (PBA) and 1.0 mg/ml WJs, using anti-HRD-1, anti-PERK, anti-phospho-IRE-1a, anti-eIF2a and anti-β-actin antibodies. The cropped images of immunoblots displayed in the figure and the full-length blots were shown in Supplementary Fig. S23 online. (c) Western blot analysis of OLETF-MSC cultured with 0 to 0.5 mg/ml WJs, using anti-α-SMA and anti-β-actin antibodies. The cropped images of immunoblots displayed in the figure and the full-length blots were shown in Supplementary Fig. S24 online. (d) Western blot analysis of OLETF-MSC cultured with or without WJs owing to the difference of an additional period from 12 to 96 hours, using anti-α-SMA and anti-β-actin antibodies. The cropped images of immunoblots displayed in the figure and the full-length blots were shown in Supplementary Fig. S25 online. (e) Immunophenotype analysis of OLETF-MSC-WJ surface antigen expression by flow cytometry. Red lines represent the target antibodies and blue lines represent the isotype controls. (f)(g) Adipogenic and osteogenic differentiation of LETO-MSC and OLETF-MSC. Fat droplets are stained red with Oil red O. Bone matrixes are stained blue by alkaline phosphatase (ALP). Bar: 100 μm (f) and 500 μm (g).

Supplementary Figure S7. Immunohistochemical analysis of pancreatic tissues in control and STZ-induced diabetic rats

Immunofluorescence staining of proinsulin (red) and glucagon (green) in islet tissues of rats. DAPI was used to counterstain nuclei (blue). Bar: 50 μm.

Supplementary Figure S8. Systolic blood pressures in STZ-induced diabetic rats

Systolic blood pressure change ratio in STZ-induced diabetic rats.

Supplementary Figure S9. Therapeutic effect of BM-MSC isolated from diabetic rats (DM-MSC) for diabetic nephropathy in OLETF diabetic rats

(a) Experimental protocol for BM-MSC therapies in OLETF diabetic rats. (b) Changes of blood glucose levels (mg/dl) after initial BM-MSC administration. Values are expressed as mean ± SE of 5–6 animals. (c) Changes of urine albumin/creatinine ratio (U-alb/Cr) after initial BM-MSC administration. Values are expressed as mean ± SE of 5–6 animals. †, $P < 0.05$ OLETF-Vehicle vs. LETO; ‡, $P < 0.05$ OLETF-Control-MSC vs. LETO; §, $P < 0.05$ OLETF-DM-MSC vs. LETO; ||, $P < 0.05$ OLETF-DM-MSC-WJ vs. LETO; ¶, $P < 0.05$ OLETF-Control-MSC vs. OLETF-Vehicle; **, $P < 0.05$ OLETF-Control-MSC vs. OLETF-DM-MSC; ††, $P < 0.05$ OLETF-DM-MSC-WJ vs. OLETF-Vehicle; ‡‡, $P < 0.05$ OLETF-DM-MSC-WJ vs. OLETF-DM-MSC. (d) Histological findings of the renal cortex in H&E-, PAS, and Azan staining kidney sections at 8 weeks after the initial administration of BM-MSC in OLETF diabetic rats. Bar: 100 µm.

Supplementary Figure S10. Distribution of STZ-MSC and STZ-MSC activated by WJs (STZ-MSC-WJ) in STZ-induced diabetic rats

Distribution of administered STZ-MSC and STZ-MSC-WJ in STZ-induced diabetic rats at days 2, 5, 8 and 15. MSCs were detected by the immunofluorescence marker PKH26 (red) in kidney (a), lung (b), liver (c) and spleen (d). DAPI was used to counterstain of nuclei (blue). White arrow shows the distribution of BM-MSC. Bar: (a) (b) (d) 100 μm, (c) 50 μm.

Supplementary Figure S11. Full unedited gel for Figure 1h

XBP-1; lane 1, Control-MSC lane 1; lane 2, Control-MSC lane 2; lane3, Control-MSC lane 3; lane 4, Control-MSC lane 4; lane 5, STZ-MSC lane 1; lane 6, STZ-MSC lane 2; lane 7, STZ-MSC lane 3; lane 8, STZ-MSC lane 4.

β-actin; lane 1, Control-MSC lane 1; lane 2, Control-MSC lane 2; lane3, Control-MSC lane 3; lane 4, Control-MSC lane 4; lane 5, STZ-MSC lane 1; lane 6, STZ-MSC lane 2; lane 7, STZ-MSC lane 3; lane 8, STZ-MSC lane 4.

Supplementary Figure S12. Full unedited gel for Figure 2h

XBP-1; lane 2, LETO-MSC lane 1; lane 3, LETO-MSC lane 2; lane4, LETO-MSC lane 3; lane 5, OLETF-MSC lane 1; lane 6, OLETF-MSC lane 2; lane 7, OLETF-MSC lane 3.

β-actin; lane 2, LETO-MSC lane 1; lane 3, LETO-MSC lane 2; lane4, LETO-MSC lane 3; lane 5, OLETF-MSC lane 1; lane 6, OLETF-MSC lane 2; lane 7, OLETF-MSC lane 3.

Supplementary Figure S13. Full unedited gel for Figure 4h

CD9; lane 2, Case No. #3; lane 3, Case No. #4; lane 4, Case No. #5; lane 5, Case No. #6; lane 6, Case No. #7; lane 7, Case No. #8.

HSP70; lane 2, Case No. #3; lane 3, Case No. #4; lane 4, Case No. #5; lane 5, Case No. #6; lane 6, Case No. #7; lane 7, Case No. #8.

β-actin; lane 2, Case No. #3; lane 3, Case No. #4; lane 4, Case No. #5; lane 5, Case No. #6; lane 6, Case No. #7; lane 7, Case No. #8.

Supplementary Figure S14. Full unedited gel for Figure 5j

BiP; lane 2, none; lane 3, Case No. #1; lane 4, Case No. #2; lane 5, Case No. #3; lane 6, Case No. #4; lane 7, Case No. #5.

XBP-1; lane 6, none; lane 7, Case No. #1; lane 8, Case No. #2; lane 9, Case No. #3; lane 10, Case No. #4; lane 11, Case No. #5.

JNK1/3; lane 5, none; lane 6, Case No. #1; lane 7, Case No. #2; lane 8, Case No. #3; lane 9, Case No. #4; lane 10, Case No. #5.

β-actin; lane 2, none; lane 3, Case No. #1; lane 4, Case No. #2; lane 5, Case No. #3; lane 6, Case No. #4; lane 7, Case No. #5.

Supplementary Figure S15. Full unedited gel for Figure 5k

α-SMA; lane 1, none; lane 2, Case No. #30; lane 9, none; lane 10, Case No. #37; lane 15, none; lane 16, Case No. #35.

β-actin; lane 1, none; lane 2, Case No. #30; lane 9, none; lane 10, Case No. #37; lane 15, none; lane 16, Case No. #35.

Supplementary Figure S16. Full unedited gel for Figure 6j

BiP; lane 2, none; lane 3, Case No. #9; lane 4, Case No. #1; lane 5, Case No. #11; lane 6, Case No. #12.

XBP-1; lane 6, none; lane 7, Case No. #9; lane 8, Case No. #1; lane 9, Case No. #11; lane 10, Case No. #12.

JNK1/3; lane 2, none; lane 3, Case No. #9; lane 4, Case No. #1; lane 5, Case No. #11; lane 6, Case No. #12.

β-actin; lane 2, none; lane 3, Case No. #9; lane 4, Case No. #1; lane 5, Case No. #11; lane 6, Case No. #12.

Supplementary Figure S17. Full unedited gel for Figure 6k

α-SMA; lane 6, none; lane 7, Case No. #9; lane 8, Case No. #1; lane 9, Case No. #11; lane 10, Case No. #12.

β-actin; lane 6, none; lane 7, Case No. #9; lane 8, Case No. #1; lane 9, Case No. #11; lane 10, Case No. #12.

Supplementary Figure S18. Full unedited gel for Figure 7e

XBP-1; lane 1, none; lane 2, WJ-#13; lane 3, WJ-#14; lane 4, WJ-#15; lane 5, WJ-#16; lane 6, WJ-#17; lane 24, WJ-exosome-#13; lane 25, WJ-exosome-#14; lane 26, WJ-exosome-#15; lane 27, WJ-exosome-#16; lane 28, WJ-exosome-#17.

α-SMA; lane 1, none; lane 2, WJ-#13; lane 3, WJ-#14; lane 4, WJ-#15; lane 5, WJ-#16; lane 6, WJ-#17; lane 24, WJ-exosome-#13; lane 25, WJ-exosome-#14; lane 26, WJ-exosome-#15; lane 27, WJ-exosome-#16; lane 28, WJ-exosome-#17.

β-actin; lane 1, none; lane 2, WJ-#13; lane 3, WJ-#14; lane 4, WJ-#15; lane 5, WJ-#16; lane 6, WJ-#17; lane 24, WJ-exosome-#13; lane 25, WJ-exosome-#14; lane 26, WJ-exosome-#15; lane 27, WJ-exosome-#16; lane 28, WJ-exosome-#17.

Supplementary Figure S19. Full unedited gel for Supplementary Figure S5b

HRD-1; lane 1, none; lane 2, PBA 5mM; lane 3, PBA 10 mM; lane 4, WJ-#18; lane 5, WJ-#19.

PERK; lane 1, none; lane 2, PBA 5mM; lane 3, PBA 10 mM; lane 4, WJ-#18; lane 5, WJ-#19.

p-IRE-1a; lane 1, none; lane 2, PBA 5mM; lane 3, PBA 10 mM; lane 4, WJ-#18; lane 5, WJ-#19.

eIF2a; lane 1, none; lane 2, PBA 5mM; lane 3, PBA 10 mM; lane 4, WJ-#18; lane 5, WJ-#19.

β-actin; lane 1, none; lane 2, PBA 5mM; lane 3, PBA 10 mM; lane 4, WJ-#18; lane 5, WJ-#19.

Supplementary Figure S20. Full unedited gel for Supplementary Figure S5c

XBP-1; lane 1, none; lane 2, TM 0.1 μg/mL; lane 3, TM 0.5 μg/mL; lane 4, TM 1.0 μg/mL; lane 5, TG 1 μg/mL; lane 6, TM 10 μg/mL; lane 7, High glucose (HG) 300 mg/dL; lane 8, High glucose (HG) 450 mg/dL.

PERK; lane 1, none; lane 2, TM 0.1 μg/mL; lane 3, TM 0.5 μg/mL; lane 4, TM 1.0 μg/mL; lane 5, TG 1 μg/mL; lane 6, TM 10 μg/mL; lane 7, High glucose (HG) 300 mg/dL; lane 8, High glucose (HG) 450 mg/dL.

β-actin; lane 1, none; lane 2, TM 0.1 μg/mL; lane 3, TM 0.5 μg/mL; lane 4, TM 1.0 μg/mL; lane 5, TG 1 μg/mL; lane 6, TM 10 μg/mL; lane 7, High glucose (HG) 300 mg/dL; lane 8, High glucose (HG) 450 mg/dL.

Supplementary Figure S21. Full unedited gel for Supplementary Figure S5d

α-SMA; lane 2, WJ-0 mg/ml; lane 3, WJ-0.1 mg/ml; lane 4, WJ-0.5 mg/ml; lane 5, WJ-1.0 mg/ml.

β-actin; lane 2, WJ-0 mg/ml; lane 3, WJ-0.1 mg/ml; lane 4, WJ-0.5 mg/ml; lane 5, WJ-1.0 mg/ml.

Supplementary Figure S22. Full unedited gel for Supplementary Figure S5e

α-SMA; lane 1, 12h WJ(-); lane 2, 12h WJ(+); lane 4, 24h WJ(-); lane 5, 24h WJ(+); lane 7, 48h WJ(-); lane 8, 48h WJ(+);

lane 10, 72h WJ(-); lane 11, 72h WJ(+); lane 13, 96h WJ(-); lane 14, 96h WJ(+).

β-actin; lane 1, 12h WJ(-); lane 2, 12h WJ(+); lane 4, 24h WJ(-); lane 5, 24h WJ(+); lane 7, 48h WJ(-); lane 8, 48h WJ(+);

lane 10, 72h WJ(-); lane 11, 72h WJ(+); lane 13, 96h WJ(-); lane 14, 96h WJ(+).

Supplementary Figure S23. Full unedited gel for Supplementary Figure S6b

HRD-1; lane 1, none; lane 2, PBA 5mM; lane 3, PBA 10 mM; lane 4, WJ-#18; lane 5, WJ-#19.

PERK; lane 1, none; lane 2, PBA 5mM; lane 3, PBA 10 mM; lane 4, WJ-#18; lane 5, WJ-#19.

p-IRE-1a; lane 1, none; lane 2, PBA 5mM; lane 3, PBA 10 mM; lane 4, WJ-#18; lane 5, WJ-#19.

eIF2a; lane 1, none; lane 2, PBA 5mM; lane 3, PBA 10 mM; lane 4, WJ-#18; lane 5, WJ-#19.

β-actin; lane 1, none; lane 2, PBA 5mM; lane 3, PBA 10 mM; lane 4, WJ-#18; lane 5, WJ-#19.

Supplementary Figure S24. Full unedited gel for Supplementary Figure S6c

α-SMA; lane 6, WJ-0 mg/ml; lane 7 WJ-0.1 mg/ml; lane 8, WJ-0.5 mg/ml.

β-actin; lane 6, WJ-0 mg/ml; lane 7 WJ-0.1 mg/ml; lane 8, WJ-0.5 mg/ml.

Supplementary Figure S25. Full unedited gel for Supplementary Figure S6d

α-SMA; lane 1, 12h WJ(-); lane 2, 12h WJ(+); lane 3, 24h WJ(-); lane 4, 24h WJ(+); lane 5, 48h WJ(-); lane 6, 48h WJ(+); lane 7, 72h WJ(-); lane 8, 72h WJ(+); lane 9, 96h WJ(-); lane 10, 96h WJ(+).

β-actin; lane 1, 12h WJ(-); lane 2, 12h WJ(+); lane 3, 24h WJ(-); lane 4, 24h WJ(+); lane 5, 48h WJ(-); lane 6, 48h WJ(+); lane 7, 72h WJ(-); lane 8, 72h WJ(+); lane 9, 96h WJ(-); lane 10, 96h WJ(+).

Supplementary Table Legends and Caption

Supplementary Table S1. Primary antibodies used for immunoblotting, immunofluorescence and immunohistochemistry

Rab, rabbit; Ms, mouse; Rt, rat; Hu, human; Sh, sheep; Chk, chicken; Dg, dog; Ho, horse; Cw, cow; GP, guinea pig; Pg, pig.

Supplementary Table S2. Secondary antibodies used for immunoblotting and immunofluorescence

Gt, goat; Dnk, donkey.

Supplementary Table S3. PCR Primers

Supplementary Tables

Supplementary Table S1. Primary antibodies used for immunoblotting, immunofluorescence and immunohistochemistry

Antibody	Species	Molecular weight (kDa)	Manufacture
**Immunoblotting**			
XBP-1	Rab	29, 42	Abcam Inc.
α-SMA	Rab	42	Abcam Inc.
β-actin	Ms	42	Sigma-Aldrich
CD9	Rab	~28	System Biosciences, Inc.
HSP70	Rab	53-70	System Biosciences, Inc.
BiP	Rab	78	Cell Signaling Technology
Jnk1/3	Rab	54, 46	Santa Cruz Biotechnology
HRD-1	Rab	67	Abcam Inc.
PERK	Rab	140	Cell Signaling Technology
p-IRE-1α	Rab	110	Novus
eIF2α	Rab	38	Cell Signaling Technology

Antibody	Species	Reactivity	Manufacture
**Immunofluorescence / Immunohistochemistry**			
Ki-67	Rab	Ms, Rt, Hu	Abcam Inc.
Hyaluronic acid	Sh	Ms, Chk, Dg, Hu	Abcam Inc.
Type 1 collagen	Rab	Ms, Rt, Ho, Cw, Hu	Abcam Inc.
Type 3 collagen	Rab	Ms, Rt, Cw, Hu	Abcam Inc.
α-SMA	Rab	Ms, Rt, Chk, GP, Cw, Hu, Pg	Abcam Inc.
MUC-1	Rab	Ms, Hu	Abcam Inc.

Insulin-Proinsulin	Ms	Ms, Rt, Cw, Hu, Pg	Abcam Inc.
Glucagon	Rab	Ms, Rt, Hu	Abcam Inc.

Rab, rabbit; Ms, mouse; Rt, rat; Hu, human; Sh, sheep; Chk, chicken; Dg, dog; Ho, horse; Cw, cow; GP, guinea pig; Pg, pig.

Supplementary Table S2. Secondary antibodies used for immunoblotting and immunofluorescence

**Antibody**	**Species**	**Conjugate**	**Manufacture**
**Immunoblotting**			
Rabbit IgG	Gt	HRP	Amersham
Mouse IgG	Gt	HRP	Amersham
**Immunofluorescence**			
Rabbit IgG	Gt	Cy3	Millipore
Sheep IgG	Dnk	Cy3	Millipore
Mouse IgG	Gt	Cy3	Millipore
Rabbit IgG	Dnk	Alexa488	Millipore

Gt, goat; Dnk, donkey.

Supplementary Table S3. PCR primers

Gene	Locus	Direction	Sequence
Igf-1	NM_178866	forward	5'- *tacttcaacaagcccacaggc* -3'
		reverse	5'- *tcagcggagcacagtacatctc* -3'
Ifn-γ	NM_138880	forward	5'- *gccaaggcacactcattgaaa* -3'
		reverse	5'- *cttttgccagttcctccagatatc* -3'
Il-1β	NM_031512	forward	5'- *ctgacagaccccaaaagattaagg* -3'
		reverse	5'- *cttgtcgagatgctgctgtga* -3'
Il-2	NM_ 053836	forward	5'- *caggccacagaattgaaacatc* -3'
		reverse	5'- *ccagcgtcttccaagtgaaag* -3'
Rantes	NM_031116	forward	5'- *tatggctcggacaccactcc* -3'
		reverse	5'- *gacaaagacgactgcaaggttg* -3'
α*-Sma*	NM_031004	forward	5'- *tccctggagaagagctacgaac* -3'
		reverse	5'- *ccaatgaaagatggctggaag* -3'
Gapdh	NM_017008	forward	5'- *atgggtgtgaaccacgagaaa* -3'
		reverse	5'- *ggatacattgggggtaggaa* -3'

Supplementary Figure S1

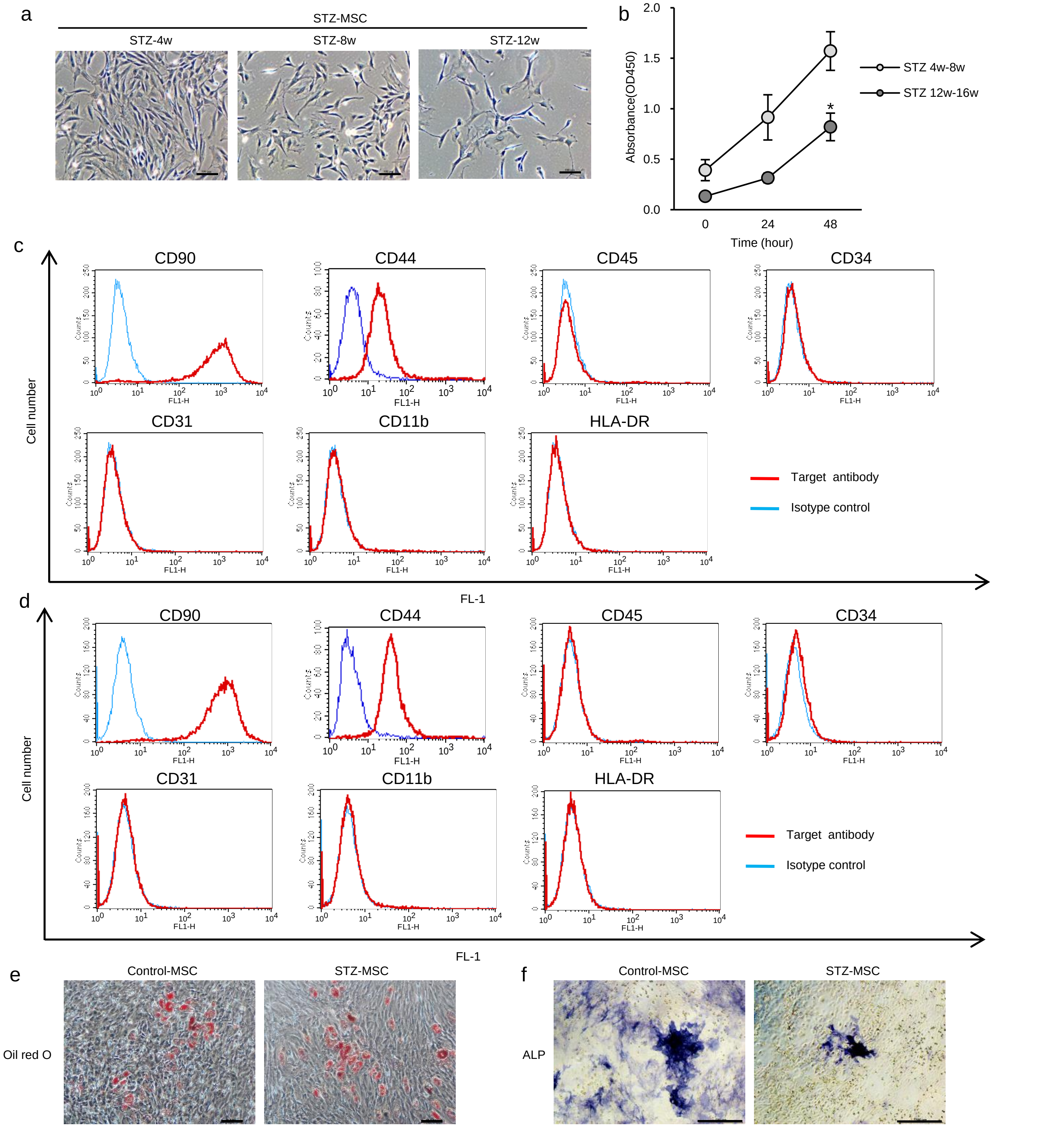

Supplementary Figure S1. Characterization of bone marrow-derived mesenchymal stem cells (BM-MSC) isolated from control rats (Control-MSC) and streptozotocin (STZ)-induced diabetic rats (STZ-MSC)

(a) Phase contrast images of BM-MSC (passage 3) isolated from 4-week-STZ loaded rats (STZ-4w), 8-week-STZ loaded rats (STZ-8w) and 12-week-STZ loaded rats (STZ-12w). Bar: 100 μm. (b) MTT proliferation assays of STZ-MSC. BM-MSC isolated from 12 to 16-week-STZ loaded rats was compared with BM-MSC isolated from 4 to 8-week-STZ loaded rats. Absorbance at 450 nm was measured at 0, 24 and 48 hours after the addition of MTT. Values are means ± SE of n=3, each. *P < 0.05. (c)(d) Immunophenotype expressions of Control-MSC (c) and STZ-MSC (d) surface antigens which were analyzed by flow cytometry. Red lines represent the target antibodies and blue lines represent the isotype controls. (e)(f) Adipogenic and osteogenic differentiation of Control-MSC and STZ-MSC. Fat droplets are stained red with Oil red O. Bone matrixes are stained blue by alkaline phosphatase (ALP). Bar: 100 μm (e) and 500 μm (f).

Supplementary Figure S2

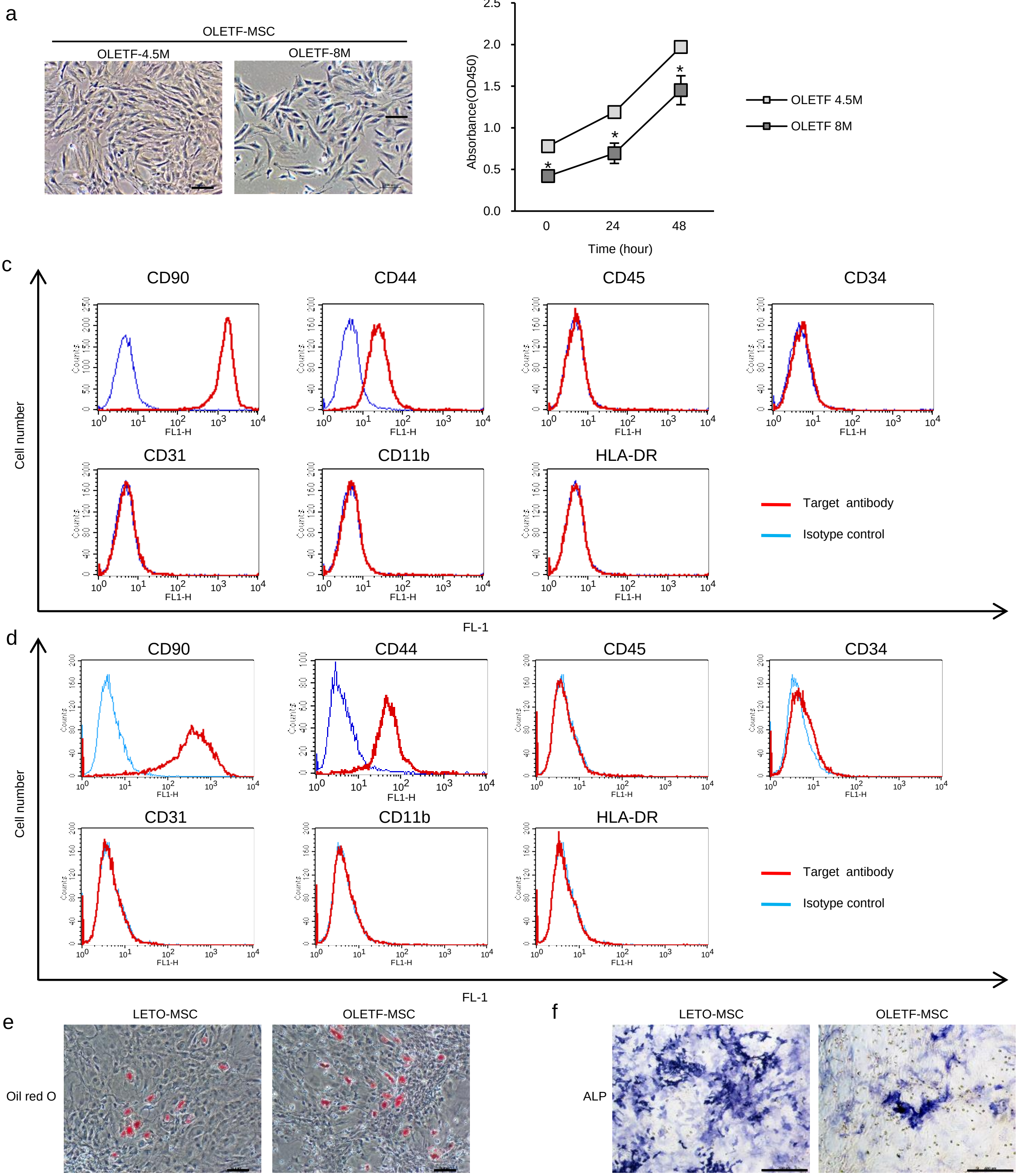

Supplementary Figure S2. Characterization of BM-MSC isolated from Long-Evans Tokushima Otsuka (LETO) rats (LETO-MSC) and Otsuka Long-Evans Tokushima Fatty (OLETF) diabetic rats (OLETF-MSC)

(a) Phase contrast images of BM-MSC (passage 3) isolated from 4.5-month-old OLETF rats (OLETF-4.5M) and 8-month-old OLETF rats (OLETF-8M). Bar: 100 μm. (b) MTT proliferation assays of OLETF-MSC compared with LETO-MSC. Absorbance at 450 nm was measured at 0, 24 and 48 hours after the addition of MTT. Values are means ± SE of n=3, each. *P < 0.05. (c)(d) Immunophenotype expressions of LETO-MSC (c) and OLETF-MSC (d) surface antigens which were analyzed by flow cytometry. Red lines represent the target antibodies and blue lines represent the isotype controls. (e)(f) Adipogenic and osteogenic differentiation of LETO-MSC and OLETF-MSC. Fat droplets are stained red with Oil red O. Bone matrixes are stained blue by alkaline phosphatase (ALP). Bar: 100 μm (e) and 500 μm (f).

Supplementary Figure S3

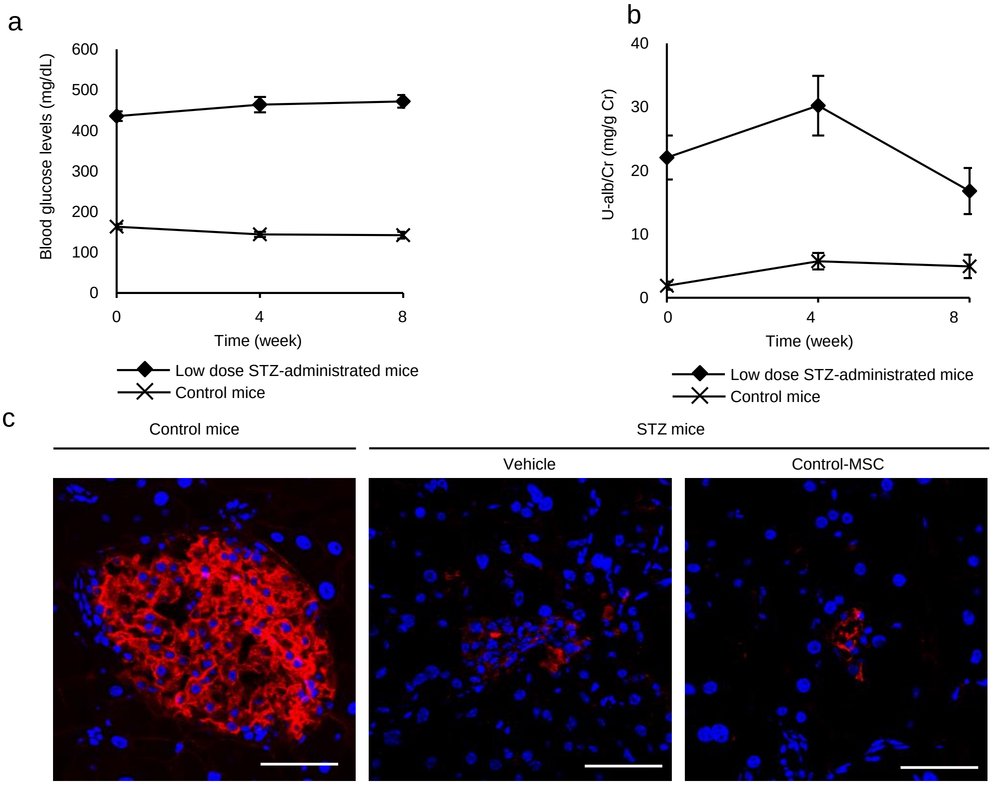

Supplementary Figure S3. Analysis of pancreas and renal function in control mice and STZ-induced diabetic mice

(a) Changes of blood glucose levels in control mice and low dose STZ (40 mg/kg) -administered mice. Values are expressed as mean ± SE of 6 animals in control group and 26 animals in STZ group. (b) Changes of urine albumin/creatinine ratio (U-alb/Cr) in control mice and low dose STZ (40 mg/kg) -administered mice. Values are expressed as mean ± SE of 6 animals in control group and 26 animals in STZ group. (c) Immunofluorescence staining of proinsulin (red) in the islet tissues of control mice and STZ-induced diabetic mice. DAPI was used for counterstaining nuclei (blue). Bar: 50 μm.

Supplementary Figure S4

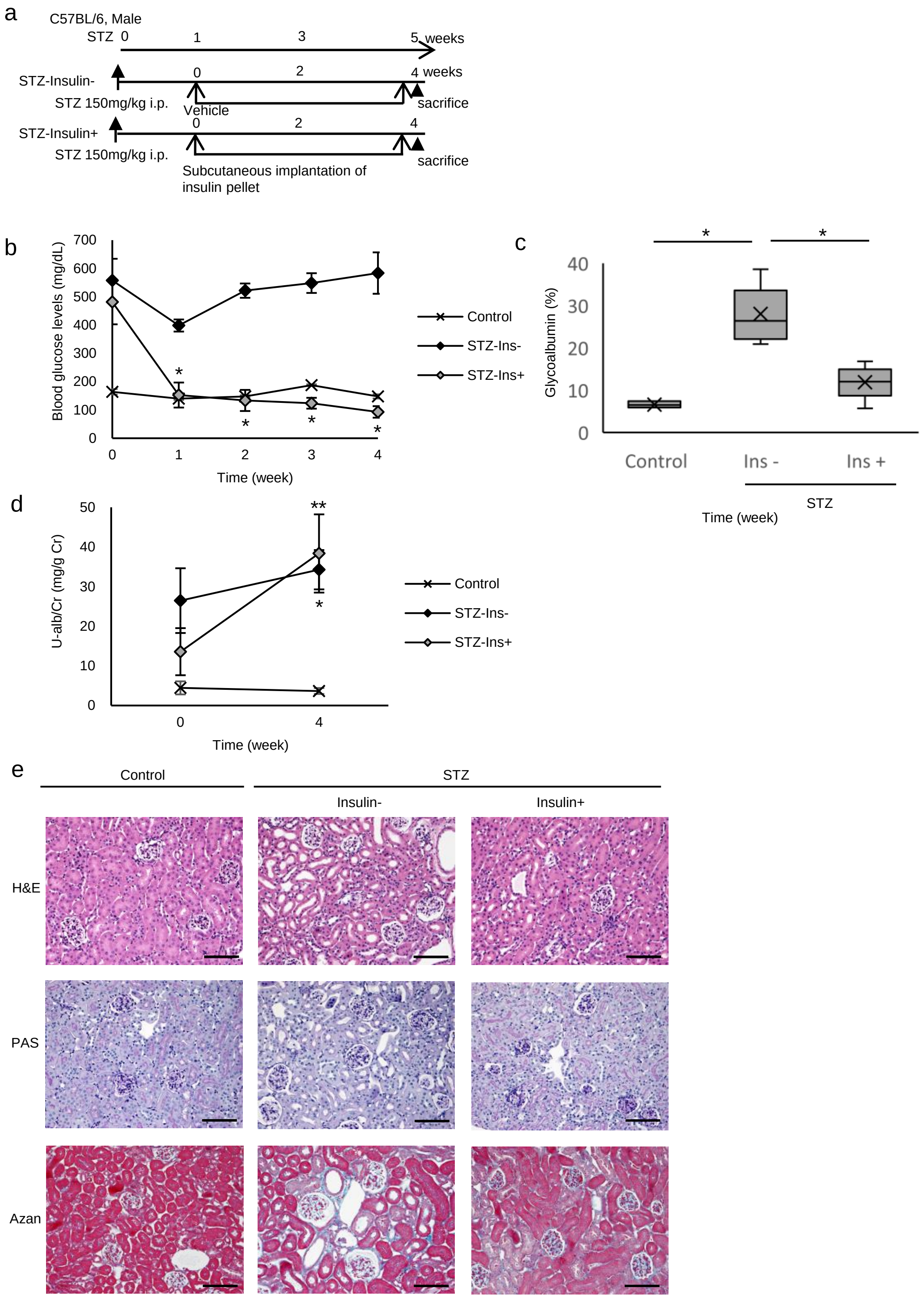

Supplementary Figure S4. Therapeutic effect of insulin pellet for diabetic nephropathy in STZ-induced diabetic mice

(a) Experimental protocol for insulin therapies in streptozotocin (STZ)-induced diabetic mice. (b) Changes in blood glucose levels after the administration of insulin pellets. Values are expressed as mean ± SE of 3–9 animals. **P* < 0.05 STZ-Ins+ vs. STZ-Ins-. (c) Glycoalbumin levels in serum at 4 weeks after the transplantation of insulin pellets. Values are expressed as mean ± SE of 3–9 animals. **P* < 0.05. (d) Changes of urine albumin/creatinine ratio (U-alb/Cr) after the transplantation of insulin pellets. Values are expressed as mean ± SE of 3–9 animals. **P* < 0.05 STZ-Ins- vs. Control, ***P* < 0.05 STZ-Ins+ vs. Control. (e) Histological findings of the renal cortex in H&E-, PAS, and Azan staining kidney sections at 4 weeks after the transplantation of insulin pellets. Bar: 100 μm.

Supplementary Figure S5

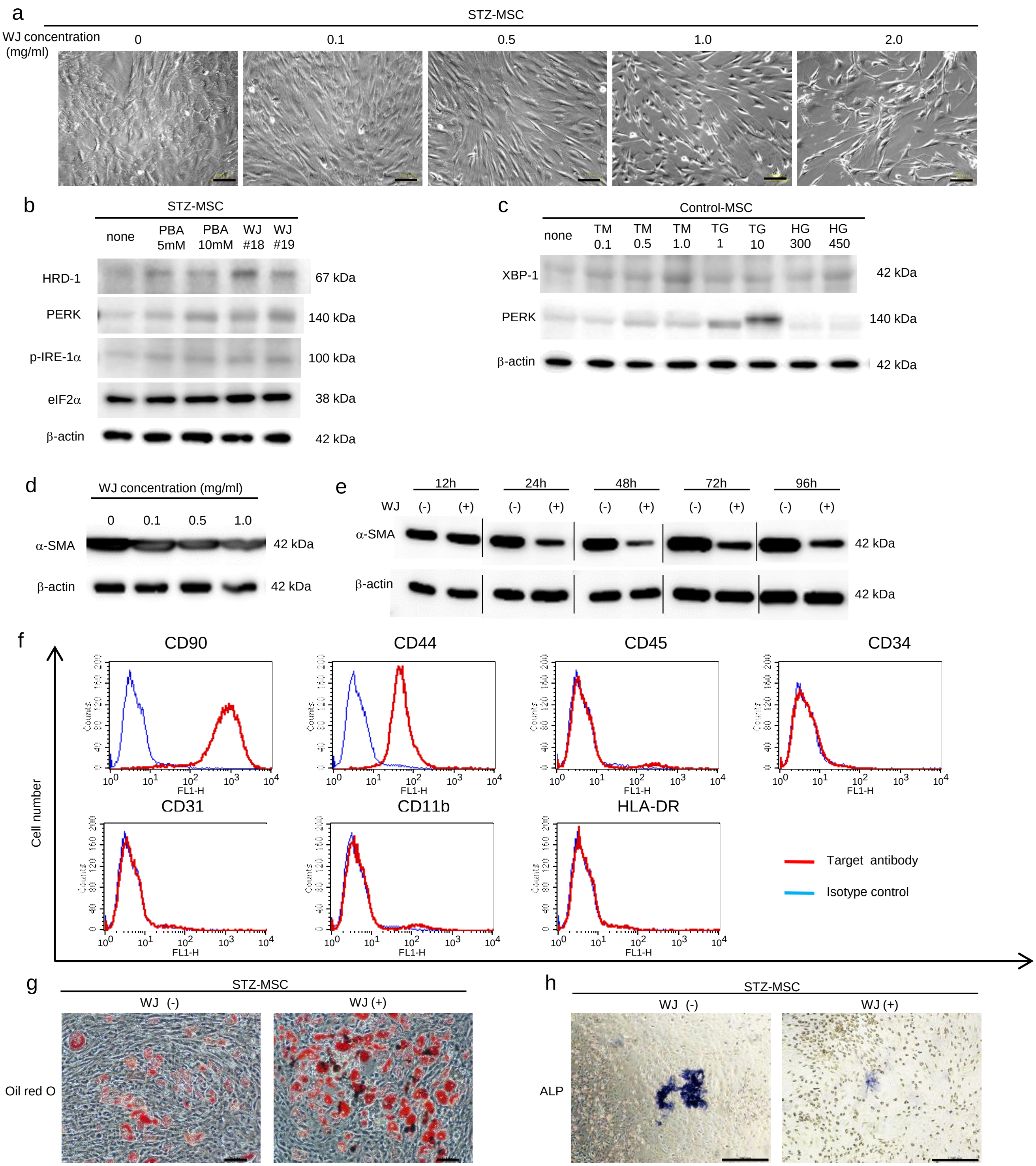

Supplementary Figure S4. Activating effects of Wharton's jelly extract supernatant (WJs) for STZ-MSC
(a) Phase contrast images of STZ-MSC cultured with 0 to 2.0 mg/ml of WJs. Bar: 100 µm. (b) Western blot analysis of STZ-MSC cultured with 5 mM or 10 mM of 4-phenylbutyric acid (PBA) and 1.0 mg/ml of WJs using anti-HRD-1, PERK, phospho-IRE-1α, eIF2α and β-actin antibodies. (c) Western blot analysis of control-MSC cultured with 0.1, 0.5, 1.0 µg/ml of Tunicamycin (TM), 1 or 10 µM of Thapsigargin (TG), 300 or 450 mg/dL of high glucose medium using anti-XBP-1, PERK and β-actin antibodies. (d) Western blot analysis of STZ-MSC cultured with 0 to 1.0 mg/ml of WJs using anti-α-SMA and β-actin antibodies. (e) Western blot analysis of STZ-MSC cultured with or without WJs owing to the difference of an additional period from 12 to 96 hours using anti-α-SMA and β-actin antibodies. (f) Immunophenotype expressions of STZ-MSC-WJ surface antigens which were analyzed by flow cytometry. Red lines represent the target antibodies and blue lines represent the isotype controls. (g)(h) Adipogenic and osteogenic differentiation of Control-MSC and STZ-MSC. Fat droplets are stained red with Oil red O. Bone matrixes are stained blue by alkaline phosphatase (ALP). Bar: 100 µm (g) and 500 µm (h).

Supplementary Figure S6

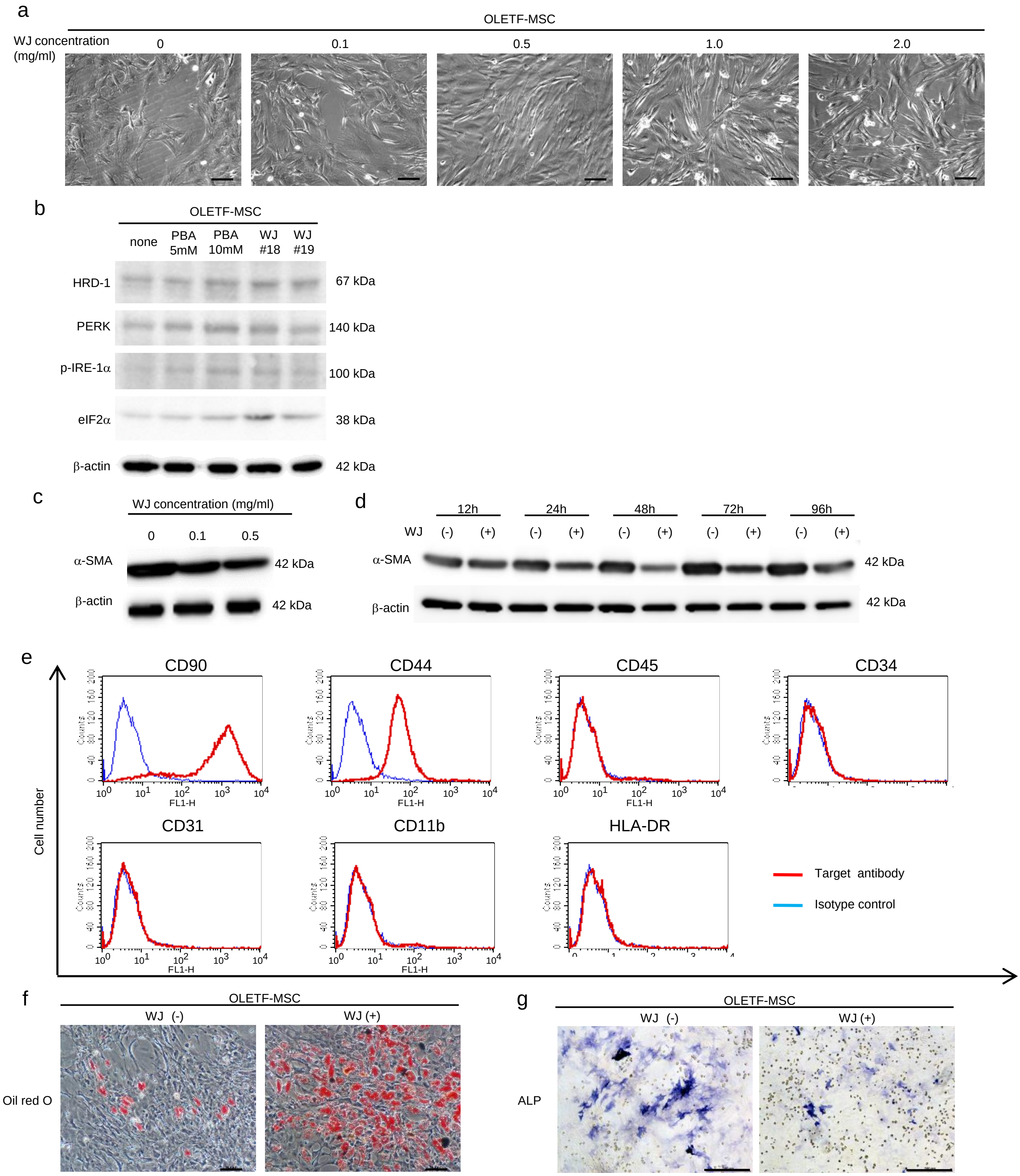

Supplementary Figure S5. Activating effects of WJs for OLETF-MSC

(a) Phase contrast images of OLETF-MSC cultured with 0 to 2.0 mg/ml of WJs. Bar: 100 µm. (b) Western blot analysis of OLETF-MSC cultured with 5 mM or 10 mM of 4-phenylbutyric acid (PBA) and 1.0 mg/ml of WJs using anti-HRD-1, PERK, phospho-IRE-1α, eIF2α and β-actin antibodies. (c) Western blot analysis of OLETF-MSC cultured with 0 to 0.5 mg/ml of WJs using anti-a-SMA and b-actin antibodies. (d) Western blot analysis of OLETF-MSC cultured with or without WJs owing to the difference of an additional period from 12 to 96 hours using anti-a-SMA and b-actin antibodies. (e) Immunophenotype expressions of OLETF-MSC-WJ surface antigens which were analyzed by flow cytometry. Red lines represent the target antibodies and blue lines represent the isotype controls. (f)(g) Adipogenic and osteogenic differentiation of LETO-MSC and OLETF-MSC. Fat droplets are stained red with Oil red O. Bone matrixes are stained blue by alkaline phosphatase (ALP). Bar: 100 µm (f) and 500 µm (g).

Supplementary Figure S7

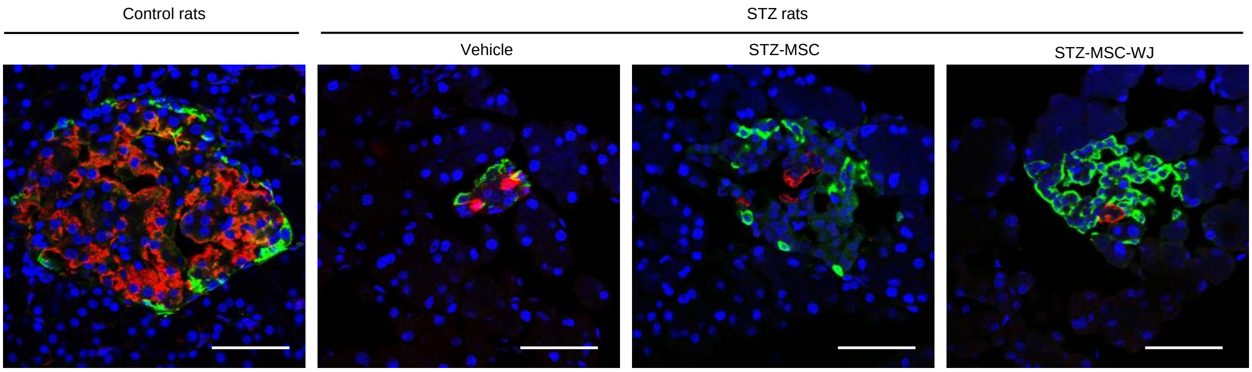

Supplementary Figure S6. Analysis of pancreas in control rats and STZ-induced diabetic rats

Immunofluorescence staining of proinsulin (red) and glucagon (green) in the islet tissues of rats. DAPI was used for counterstaining nuclei (blue). Bar: 50 µm.

Supplementary Figure S8

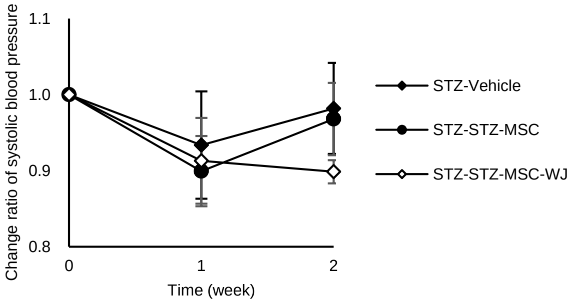

Supplementary Figure S7. Systolic blood pressures in STZ-induced diabetic rats

Change ratio of systolic blood pressure in STZ-induced diabetic rats.

Supplementary Figure S9

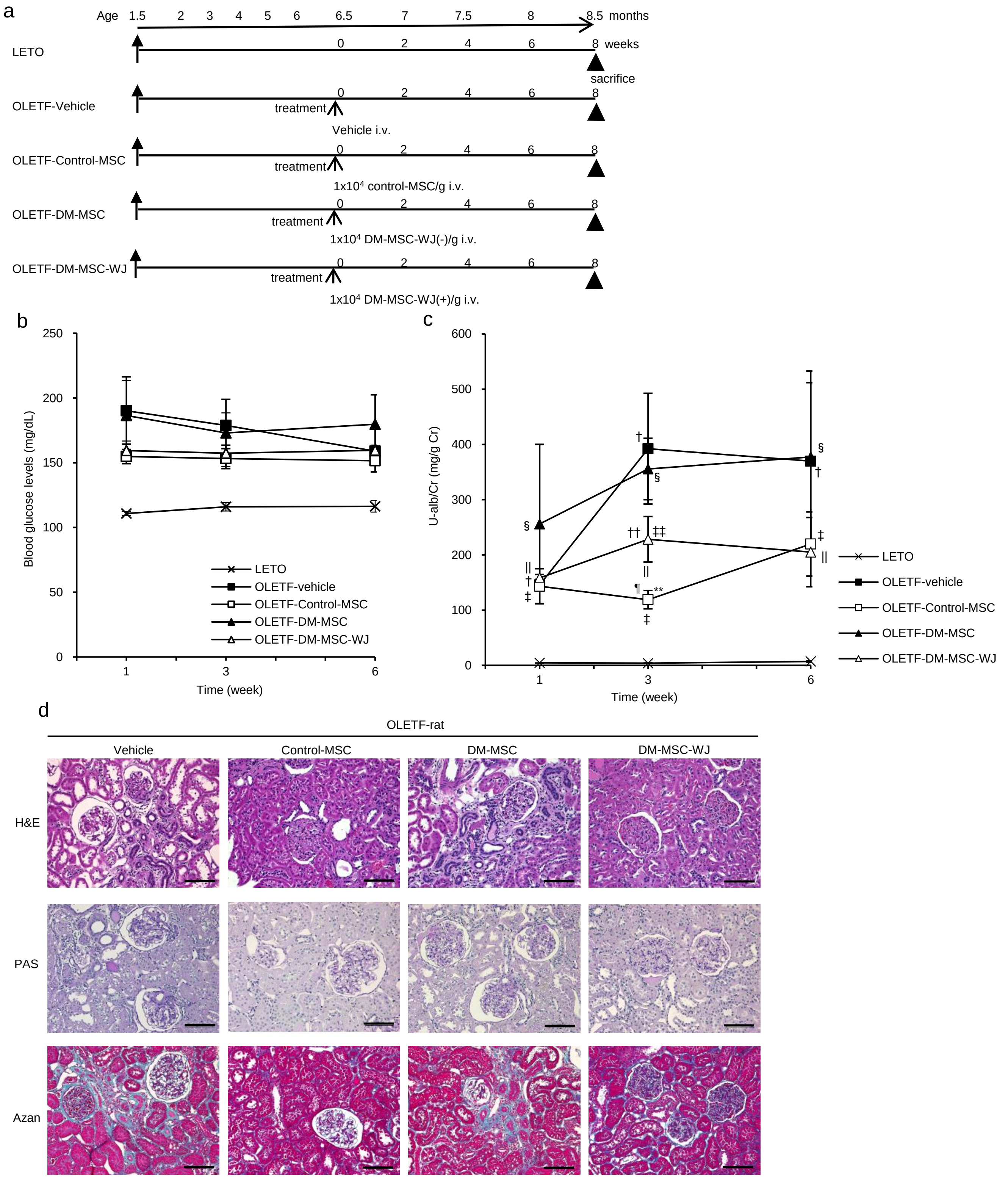

Supplementary Figure S9. Therapeutic effect of BM-MSC isolated from diabetic rats (DM-MSC) for diabetic nephropathy in OLETF diabetic rats (a) Experimental protocol for BM-MSC therapies in OLETF diabetic rats. (b) Cchanges of fasting blood glucose levels after initial BM-MSC administration. Values are expressed as mean ± SE of 5–6 animals. (c) Changes of urine albumin/creatinine ratio (U-alb/Cr) after initial BM-MSC administration. Values are expressed as mean ± SE of 5–6 animals. †, P < 0.05 OLETF-Vehicle vs. LETO; ‡, P < 0.05 OLETF-Control-MSC vs. LETO; §, P < 0.05 OLETF-DM-MSC vs. LETO; ||, P < 0.05 OLETF-DM-MSC-WJ vs. LETO; ¶, P < 0.05 OLETF-Control-MSC vs. OLETF-Vehicle; **, P < 0.05 OLETF-Control-MSC vs. OLETF-DM-MSC; ††, P < 0.05 OLETF-DM-MSC-WJ vs. OLETF-Vehicle; ‡‡, P < 0.05 OLETF-DM-MSC-WJ vs. OLETF-DM-MSC. (d) Histological findings of the renal cortex in H&E-, PAS, and Azan staining kidney sections at 8 weeks after the initial administration of BM-MSC in OLETF diabetic rats. Bar: 100 μm.

Supplementary Figure S10

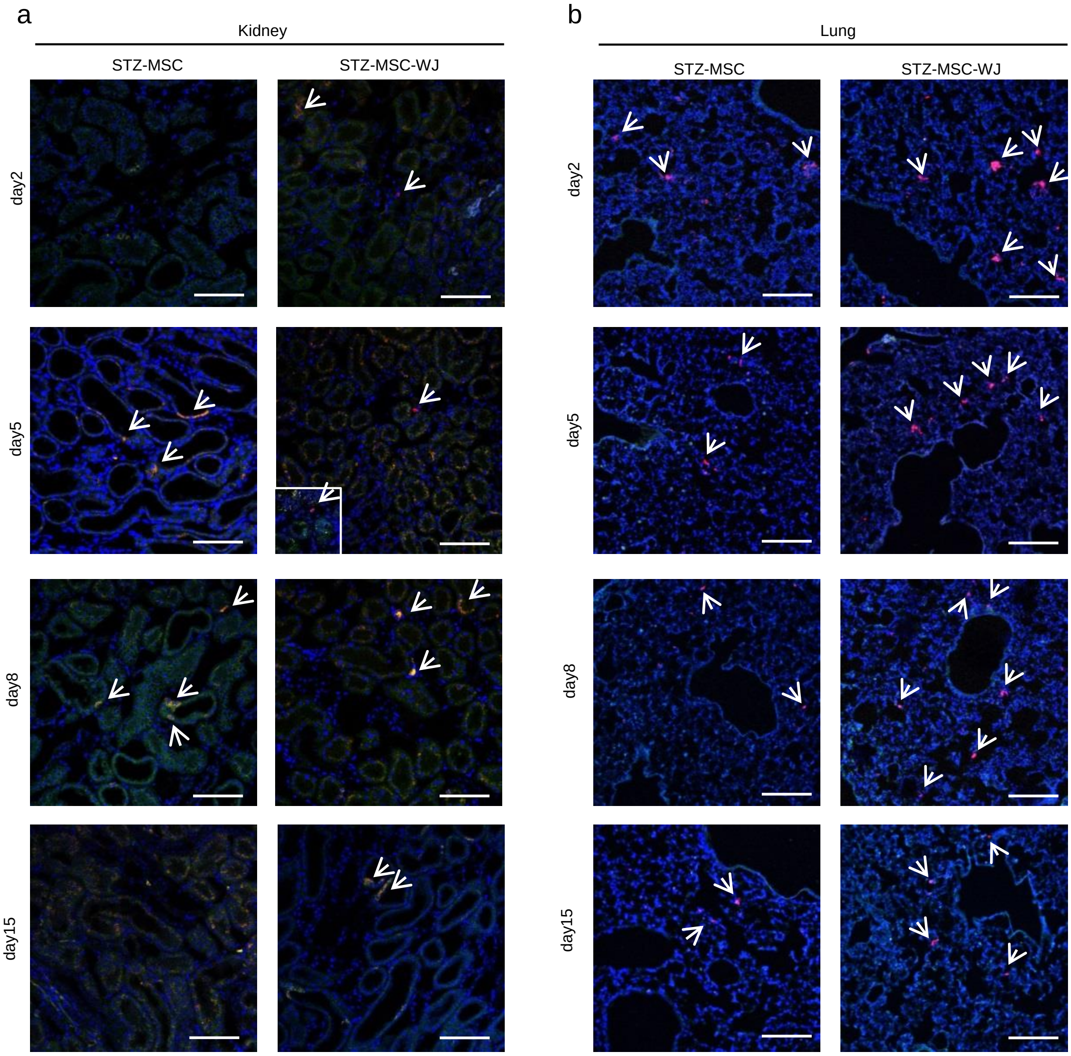

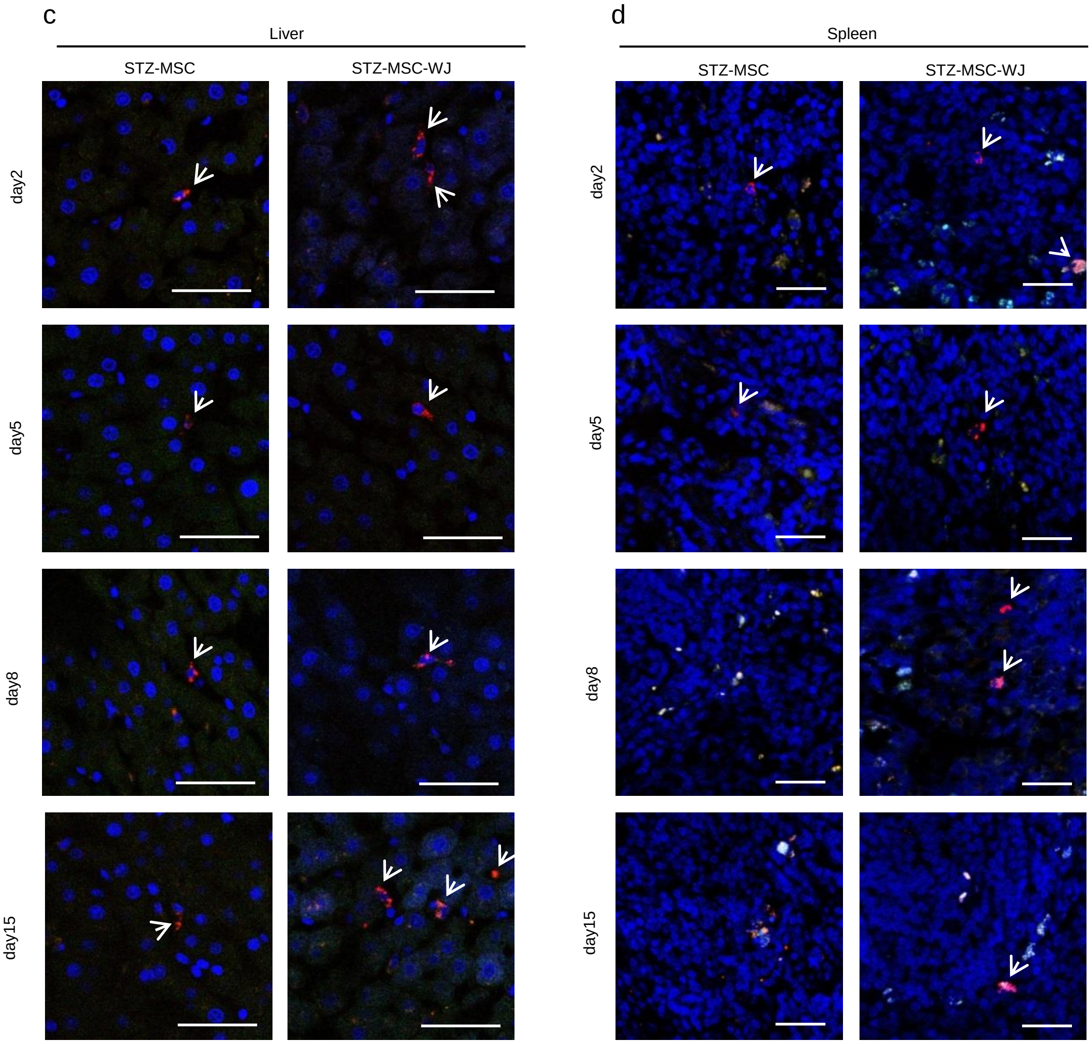

Supplementary Figure S8. Distribution of STZ-MSC and STZ-MSC activated with WJs (STZ-MSC-WJ) in STZ-induced diabetic rats

Distribution of administered STZ-MSC and STZ-MSC-WJ in STZ-induced diabetic rats at days 2, 5, 8 and 15. MSCs were detected with the immunofluorescence marker PKH26 (red) in kidney (a), lung (b), liver (c) and spleen (d). DAPI was used for counterstaining of nuclei (blue). White arrow shows the distribution of BM-MSC. Bar: (a) (b) (d) 100 µm, (c) 50 µm.

Supplementary Figure S11

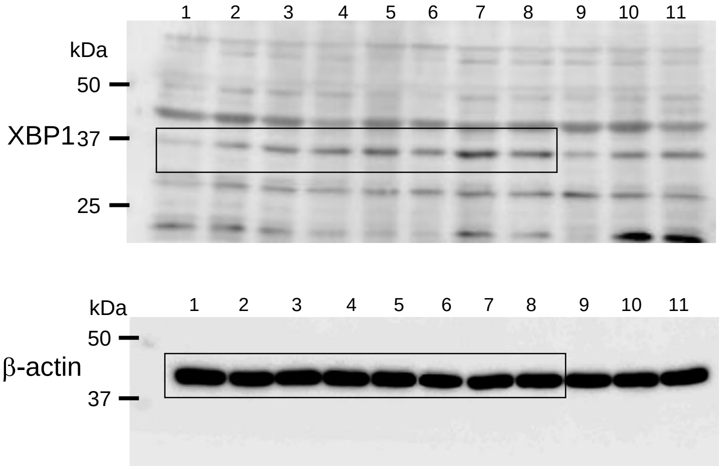

Supplementary Figure S11. Full unedited gel for Figure 1h
XBP-1; lane 1, Control-MSC lane 1; lane 2, Control-MSC lane 2; lane3, Control-MSC lane 3; lane 4, Control-MSC lane 4; lane 5, STZ-MSC lane 1; lane 6, STZ-MSC lane 2; lane 7, STZ-MSC lane 3; lane 8, STZ-MSC lane 4.
β–actin; lane 1, Control-MSC lane 1; lane 2, Control-MSC lane 2; lane3, Control-MSC lane 3; lane 4, Control-MSC lane 4; lane 5, STZ-MSC lane 1; lane 6, STZ-MSC lane 2; lane 7, STZ-MSC lane 3; lane 8, STZ-MSC lane 4.

Supplementary Figure S12

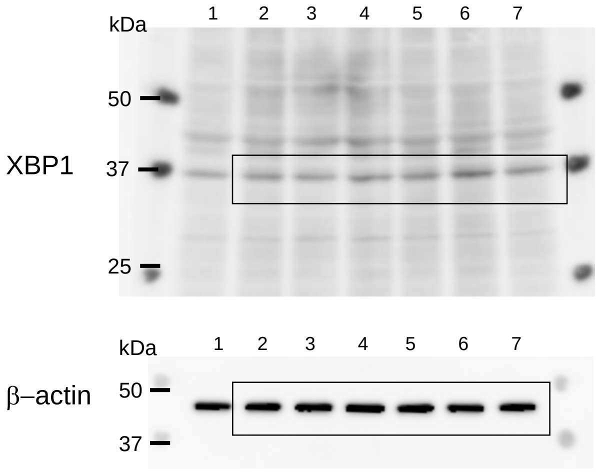

Supplementary Figure S12. Full unedited gel for Figure 2h
XBP-1; lane 2, LETO-MSC lane 1; lane 3, LETO-MSC lane 2; lane4, LETO-MSC lane 3; lane 5, OLETF-MSC lane 1; lane 6, OLETF-MSC lane 2; lane 7, OLETF-MSC lane 3.
β–actin; lane 2, LETO-MSC lane 1; lane 3, LETO-MSC lane 2; lane4, LETO-MSC lane 3; lane 5, OLETF-MSC lane 1; lane 6, OLETF-MSC lane 2; lane 7, OLETF-MSC lane 3.

Supplementary Figure S13

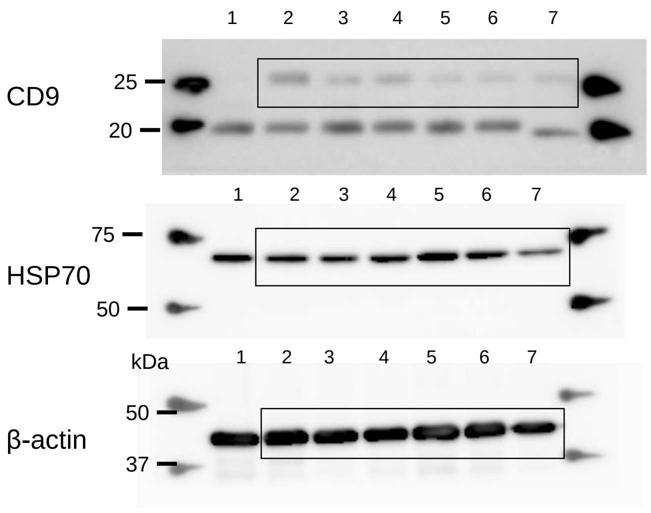

Supplementary Figure S13. Full unedited gel for Figure 4h
CD9; lane 2, Case No. #3; lane 3, Case No. #4; lane 4, Case No. #5; lane 5, Case No. #6; lane 6, Case No. #7; lane 7, Case No. #8.
HSP70; lane 2, Case No. #3; lane 3, Case No. #4; lane 4, Case No. #5; lane 5, Case No. #6; lane 6, Case No. #7; lane 7, Case No. #8.
β–actin; lane 2, Case No. #3; lane 3, Case No. #4; lane 4, Case No. #5; lane 5, Case No. #6; lane 6, Case No. #7; lane 7, Case No. #8.

Supplementary Figure S14

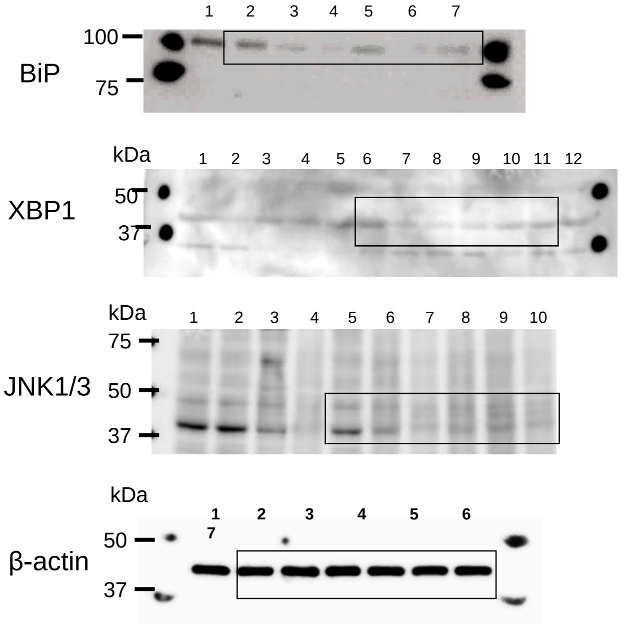

Supplementary Figure S14. Full unedited gel for Figure 5j
BiP; lane 2, none; lane 3, Case No. #1; lane 4, Case No. #2; lane 5, Case No. #3; lane 6, Case No. #4; lane 7, Case No. #5.
XBP-1; lane 6, none; lane 7, Case No. #1; lane 8, Case No. #2; lane 9, Case No. #3; lane 10, Case No. #4; lane 11, Case No. #5.
JNK1/3; lane 5, none; lane 6, Case No. #1; lane 7, Case No. #2; lane 8, Case No. #3; lane 9, Case No. #4; lane 10, Case No. #5.
β–actin; lane 2, none; lane 3, Case No. #1; lane 4, Case No. #2; lane 5, Case No. #3; lane 6, Case No. #4; lane 7, Case No. #5.

Supplementary Figure S15

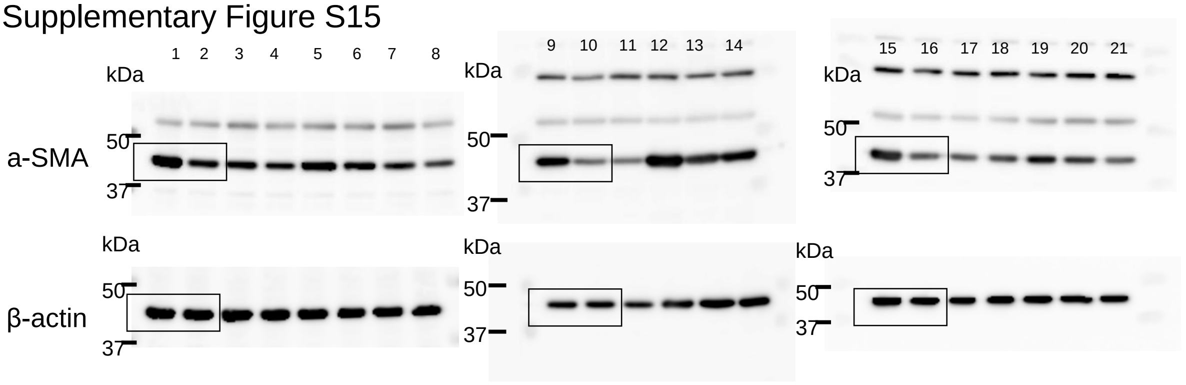

Supplementary Figure S15. Full unedited gel for Figure 5k
α−SMA; lane 1, none; lane 2, Case No. #30; lane 9, none; lane 10, Case No. #37; lane 15, none; lane 16, Case No. #35.
β−actin; lane 1, none; lane 2, Case No. #30; lane 9, none; lane 10, Case No. #37; lane 15, none; lane 16, Case No. #35.

Supplementary Figure S16

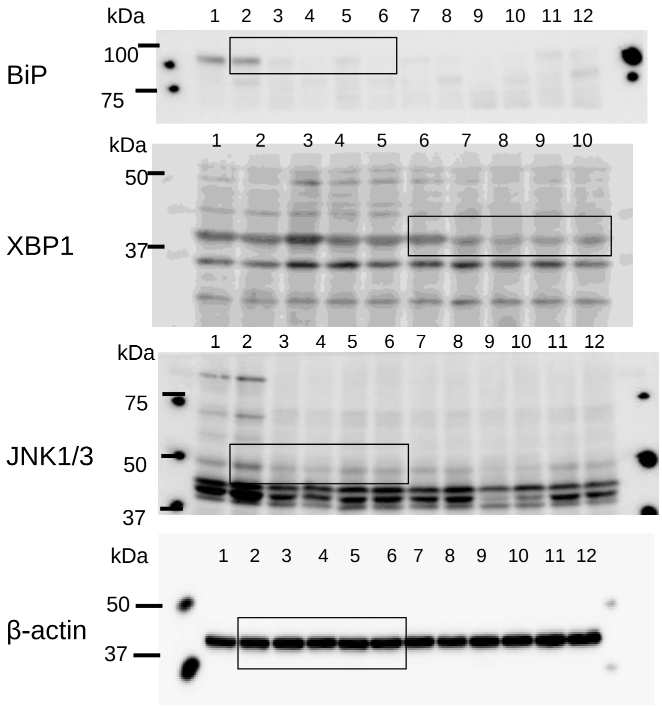

Supplementary Figure S16. Full unedited gel for Figure 6j
BiP; lane 2, none; lane 3, Case No. #9; lane 4, Case No. #1; lane 5, Case No. #11; lane 6, Case No. #12.
XBP-1; lane 6, none; lane 7, Case No. #9; lane 8, Case No. #1; lane 9, Case No. #11; lane 10, Case No. #12.
JNK1/3; lane 2, none; lane 3, Case No. #9; lane 4, Case No. #1; lane 5, Case No. #11; lane 6, Case No. #12.
β−actin; lane 2, none; lane 3, Case No. #9; lane 4, Case No. #1; lane 5, Case No. #11; lane 6, Case No. #12.

Supplementary Figure S17

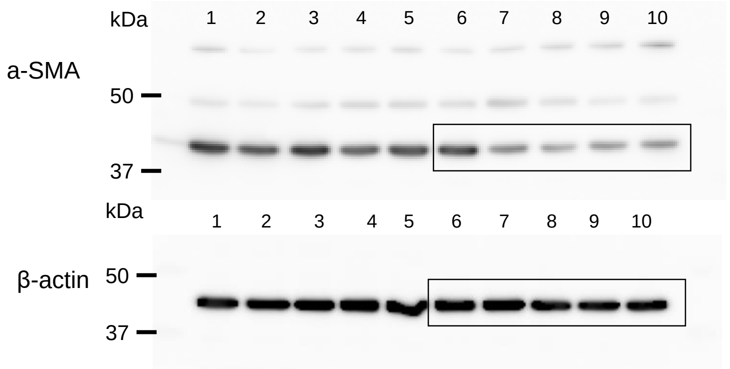

Supplementary Figure S17. Full unedited gel for Figure 6k

α−SMA; lane 6, none; lane 7, Case No. #9; lane 8, Case No. #1; lane 9, Case No. #11; lane 10, Case No. #12.

β−actin; lane 6, none; lane 7, Case No. #9; lane 8, Case No. #1; lane 9, Case No. #11; lane 10, Case No. #12.

Supplementary Figure S18

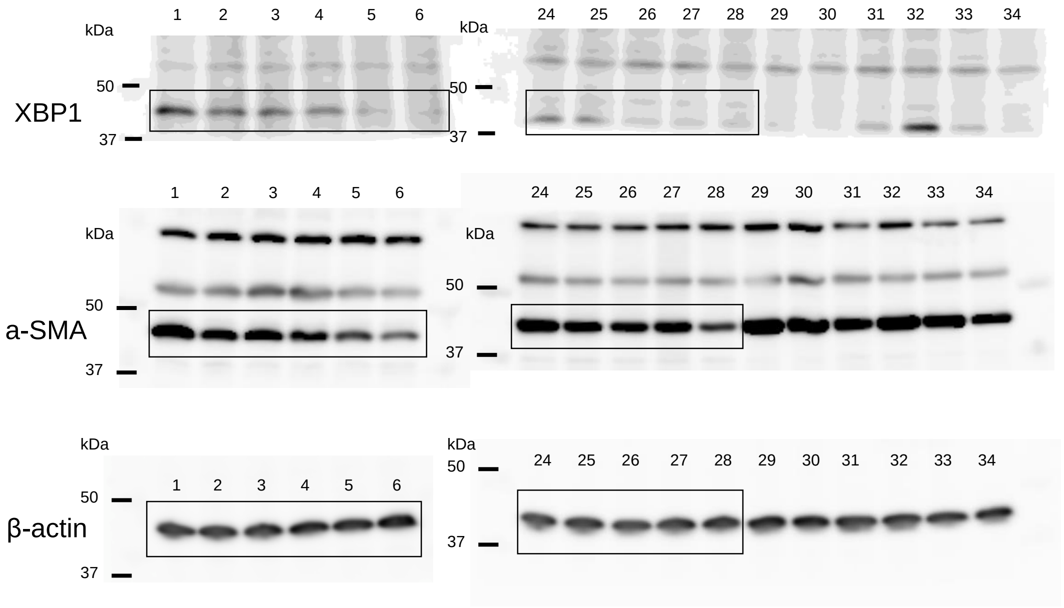

Supplementary Figure S18. Full unedited gel for Figure 7e

XBP-1; lane 1, none; lane 2, WJ-#13; lane 3, WJ-#14; lane 4, WJ-#15; lane 5, WJ-#16; lane 6, WJ-#17; lane 24, WJ-exosome-#13; lane 25, WJ-exosome-#14; lane 26, WJ-exosome-#15; lane 27, WJ-exosome-#16; lane 28, WJ-exosome-#17.

α−SMA; lane 1, none; lane 2, WJ-#13; lane 3, WJ-#14; lane 4, WJ-#15; lane 5, WJ-#16; lane 6, WJ-#17; lane 24, WJ-exosome-#13; lane 25, WJ-exosome-#14; lane 26, WJ-exosome-#15; lane 27, WJ-exosome-#16; lane 28, WJ-exosome-#17.

β−actin; lane 1, none; lane 2, WJ-#13; lane 3, WJ-#14; lane 4, WJ-#15; lane 5, WJ-#16; lane 6, WJ-#17; lane 24, WJ-exosome-#13; lane 25, WJ-exosome-#14; lane 26, WJ-exosome-#15; lane 27, WJ-exosome-#16; lane 28, WJ-exosome-#17.

Supplementary Figure S19

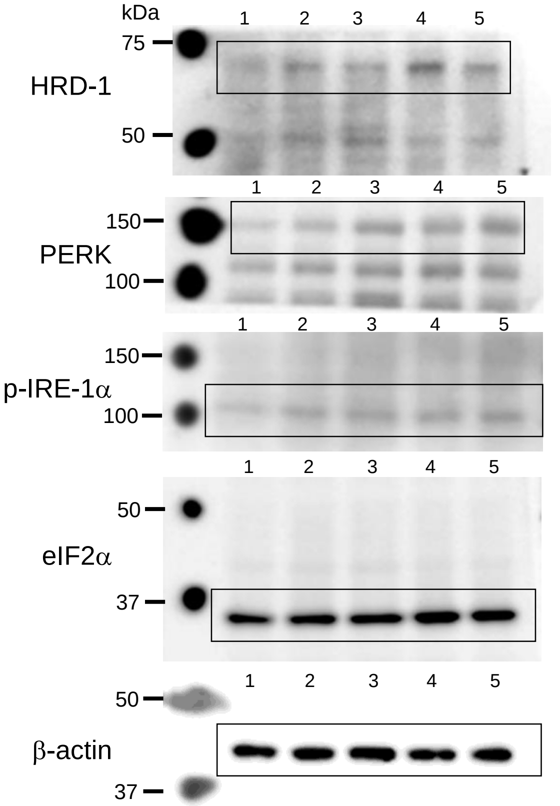

Supplementary Figure S19. Full unedited gel for Supplementary Figure S5b
HRD-1; lane 1, none; lane 2, PBA 5mM; lane 3, PBA 10 mM; lane 4, WJ-#18; lane 5, WJ-#19.
PERK; lane 1, none; lane 2, PBA 5mM; lane 3, PBA 10 mM; lane 4, WJ-#18; lane 5, WJ-#19.
p-IRE-1α; lane 1, none; lane 2, PBA 5mM; lane 3, PBA 10 mM; lane 4, WJ-#18; lane 5, WJ-#19.
eIF2α; lane 1, none; lane 2, PBA 5mM; lane 3, PBA 10 mM; lane 4, WJ-#18; lane 5, WJ-#19.
β–actin; lane 1, none; lane 2, PBA 5mM; lane 3, PBA 10 mM; lane 4, WJ-#18; lane 5, WJ-#19.

Supplementary Figure S20

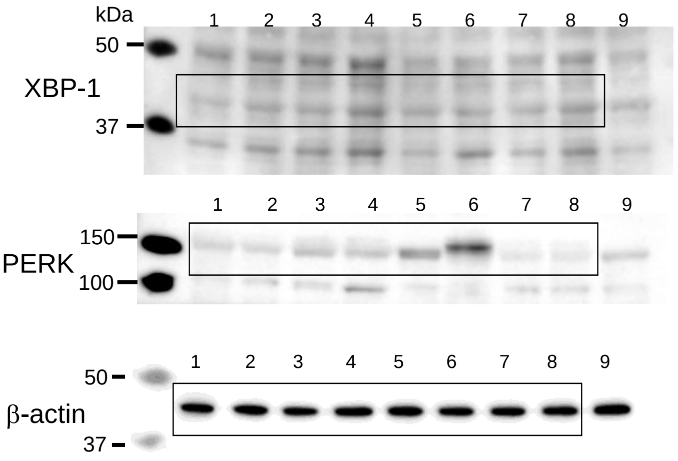

Supplementary Figure S20. Full unedited gel for Supplementary Figure S5c
XBP-1; lane 1, none; lane 2, TM 0.1 mg/mL; lane 3, TM 0.5 mg/mL; lane 4, TM 1.0 mg/mL; lane 5, TG 1 mg/mL; lane 6, TM 10 mg/mL; lane 7, High glucose (HG) 300 mg/dL; lane 8, High glucose (HG) 450 mg/dL.
PERK; lane 1, none; lane 2, TM 0.1 mg/mL; lane 3, TM 0.5 mg/mL; lane 4, TM 1.0 mg/mL; lane 5, TG 1 mg/mL; lane 6, TM 10 mg/mL; lane 7, High glucose (HG) 300 mg/dL; lane 8, High glucose (HG) 450 mg/dL.
b-actin; lane 1, none; lane 2, TM 0.1 mg/mL; lane 3, TM 0.5 mg/mL; lane 4, TM 1.0 mg/mL; lane 5, TG 1 mg/mL; lane 6, TM 10 mg/mL; lane 7, High glucose (HG) 300 mg/dL; lane 8, High glucose (HG) 450 mg/dL.

Supplementary Figure S21

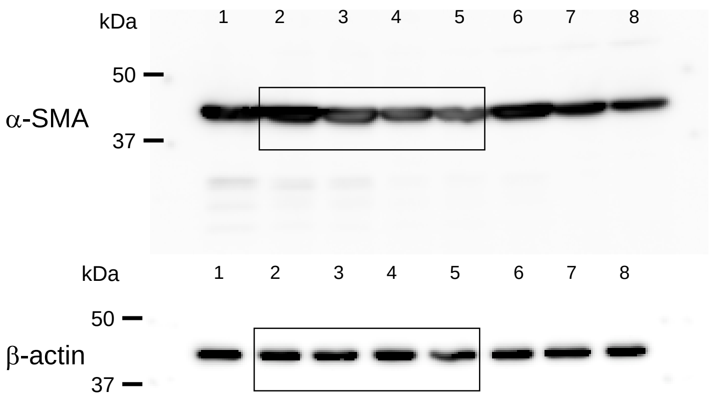

Supplementary Figure S21. Full unedited gel for Supplementary Figure S5d
α–SMA; lane 2, WJ-0 mg/ml; lane 3, WJ-0.1 mg/ml; lane 4, WJ-0.5 mg/ml; lane 5, WJ-1.0 mg/ml.
β–actin; lane 2, WJ-0 mg/ml; lane 3, WJ-0.1 mg/ml; lane 4, WJ-0.5 mg/ml; lane 5, WJ-1.0 mg/ml.

Supplementary Figure S22

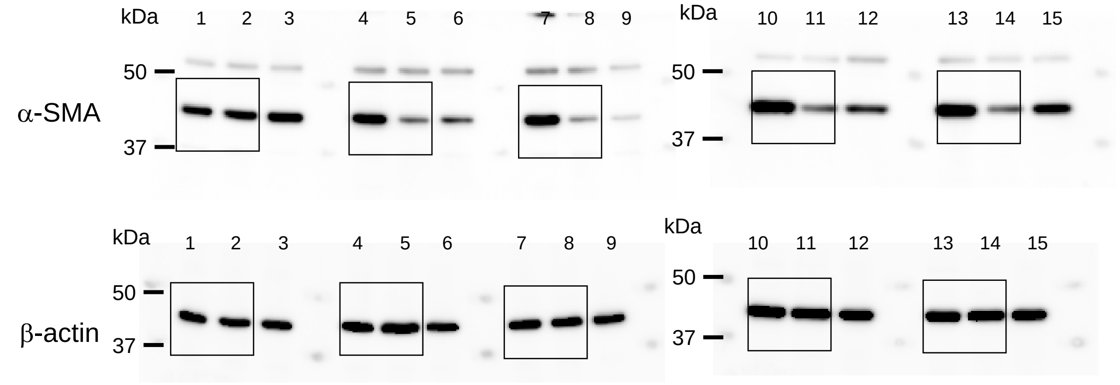

Supplementary Figure S22. Full unedited gel for Supplementary Figure S5e
α–SMA; lane 1, 12h WJ(-); lane 2, 12h WJ(+); lane 4, 24h WJ(-); lane 5, 24h WJ(+); lane 7, 48h WJ(-); lane 8, 48h WJ(+); lane 10, 72h WJ(-); lane 11, 72h WJ(+); lane 13, 96h WJ(-); lane 14, 96h WJ(+).
β–actin; lane 1, 12h WJ(-); lane 2, 12h WJ(+); lane 4, 24h WJ(-); lane 5, 24h WJ(+); lane 7, 48h WJ(-); lane 8, 48h WJ(+); lane 10, 72h WJ(-); lane 11, 72h WJ(+); lane 13, 96h WJ(-); lane 14, 96h WJ(+).

Supplementary Figure S23

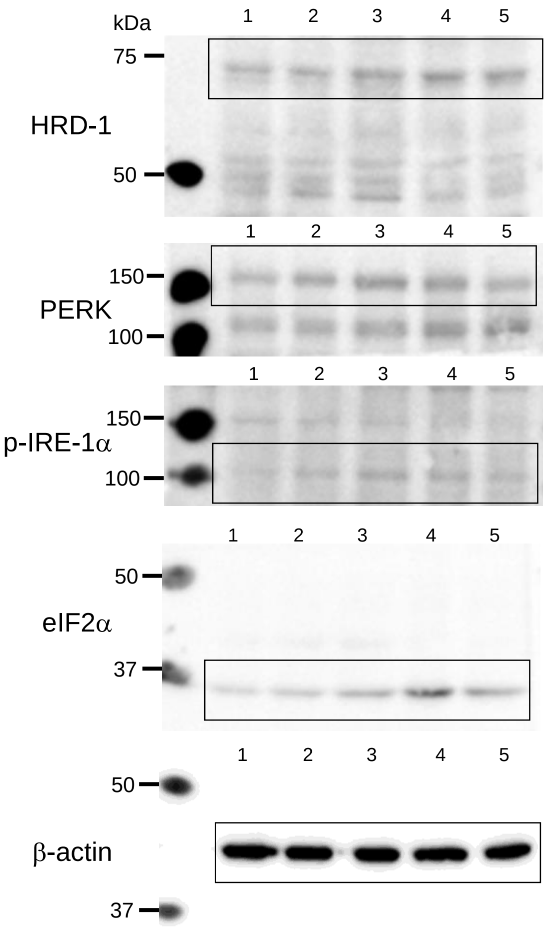

Supplementary Figure S23. Full unedited gel for Supplementary Figure S6b

HRD-1; lane 1, none; lane 2, PBA 5mM; lane 3, PBA 10 mM; lane 4, WJ-#18; lane 5, WJ-#19.
PERK; lane 1, none; lane 2, PBA 5mM; lane 3, PBA 10 mM; lane 4, WJ-#18; lane 5, WJ-#19.
p-IRE-1α; lane 1, none; lane 2, PBA 5mM; lane 3, PBA 10 mM; lane 4, WJ-#18; lane 5, WJ-#19.
eIF2α; lane 1, none; lane 2, PBA 5mM; lane 3, PBA 10 mM; lane 4, WJ-#18; lane 5, WJ-#19.
β–actin; lane 1, none; lane 2, PBA 5mM; lane 3, PBA 10 mM; lane 4, WJ-#18; lane 5, WJ-#19.

Supplementary Figure S24

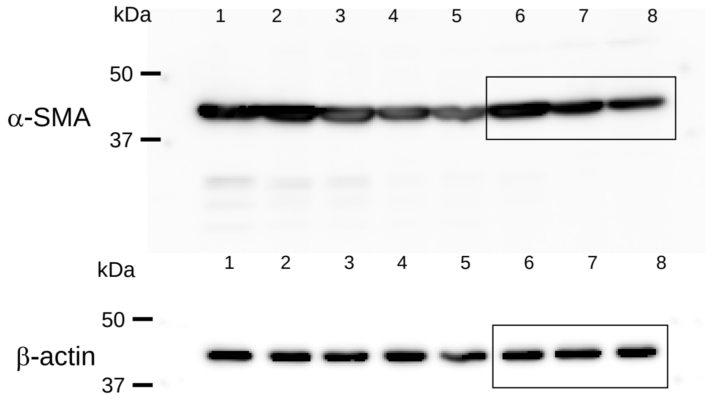

Supplementary Figure S24. Full unedited gel for Supplementary Figure S6c
α–SMA; lane 6, WJ-0 mg/ml; lane 7 WJ-0.1 mg/ml; lane 8, WJ-0.5 mg/ml.
β–actin; lane 6, WJ-0 mg/ml; lane 7 WJ-0.1 mg/ml; lane 8, WJ-0.5 mg/ml.

Supplementary Figure S25

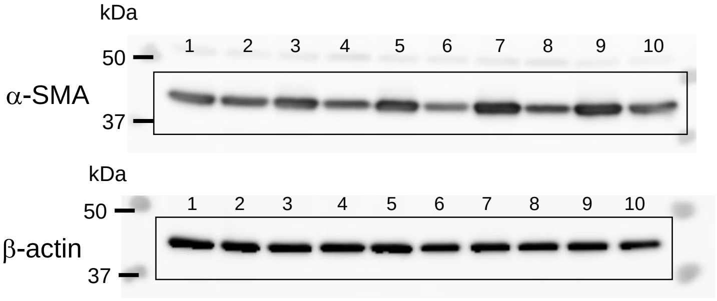

Supplementary Figure S25. Full unedited gel for Supplementary Figure S6d
α–SMA; lane 1, 12h WJ(-); lane 2, 12h WJ(+); lane 3, 24h WJ(-); lane 4, 24h WJ(+); lane 5, 48h WJ(-); lane 6, 48h WJ(+); lane 7, 72h WJ(-); lane 8, 72h WJ(+); lane 9, 96h WJ(-); lane 10, 96h WJ(+).
β–actin; lane 1, 12h WJ(-); lane 2, 12h WJ(+); lane 3, 24h WJ(-); lane 4, 24h WJ(+); lane 5, 48h WJ(-); lane 6, 48h WJ(+); lane 7, 72h WJ(-); lane 8, 72h WJ(+); lane 9, 96h WJ(-); lane 10, 96h WJ(+).
